# Coordinative Ring‐Opening Polymerization of Limonene Carbamate Toward Phosgene‐ and Isocyanate‐Free Polyurethane

**DOI:** 10.1002/anie.202502727

**Published:** 2025-07-23

**Authors:** Jonas Futter, Leon F. Richter, Stefanie Hörl, Moritz Kränzlein, Bernhard Rieger

**Affiliations:** ^1^ Wacker‐Lehrstuhl für Makromolekulare Chemie Catalysis Research Center TUM School of Natural Sciences Technische Universität München Lichtenbergstraße 4 85748 Garching Garching bei München Germany; ^2^ Molecular Catalysis Catalysis Research Center TUM School of Natural Sciences Technische Universität München Lichtenbergstraße 4 85748 Garching Garching bei München Germany

**Keywords:** Isocyanate‐free synthesis, Limonene‐based polymers, Phosgene‐free synthesis, Polyurethane, Ring‐opening polymerization

## Abstract

This study presents a phosgene‐ and isocyanate‐free route for the synthesis of polyurethanes using (*R*)‐limonene as a bio‐based starting material. The synthesis of the cyclic limonene‐based carbamate monomer **LU** is achieved in high yields using dimethyl carbonate as a sustainable, less hazardous phosgene surrogate and is verified by NMR, SC‐XRD, ESI‐MS, GC‐MS, and elemental analysis. Polymerizations were carried out by coordinative ring‐opening polymerization. Sn(Oct)_2_ showed the best catalytic performance, achieving up to 93% conversion with molecular weights up to 16.0 kg mol^−1^ at a polydispersity of 1.5. Detailed mechanistic insights were obtained by kinetic studies, end group determination via ESI‐MS and stoichiometric ninhydrin experiments, *N*–*H* methylation of **LU**, kinetic isotope experiments, and ^119^Sn NMR measurements. The resulting semi‐crystalline polyurethane exhibits promising thermal properties, including a decomposition temperature of 252 °C and a glass transition temperature above 150 °C. Depolymerization in solution was achieved in high yield using the same catalyst. This work describes a coordinative ring‐opening polymerization approach using a terpene‐based carbamate as monomer, an important step toward bio‐based polyurethanes.

## Graphical abstract

This study presents a phosgene‐ and isocyanate‐free route to terpene‐based polyurethanes using (*R*)‐limonene as a bio‐based feedstock. Through ring‐opening polymerization (ROP), limonene carbamate (**LU**) is converted into polyurethane (**PLU**) with high efficiency. Mechanistic insights are provided based on kinetic studies, end group analysis, and kinetic isotope effect experiments. Thermal analyses reveal promising material properties and chemical circularity.

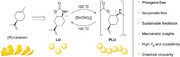

**Abbreviations**
A1: Benzoyl limonene carbamate activator
*m*‐CPBA: *meta*‐Chloroperoxybenzoic acid
DCM: Dichloromethane
*d*‐LU: Deuterated limonene carbamate
DMC: Dimethyl carbonate
DMF: Dimethylformamide
DSC: Differential scanning calorimetry;
ESI‐MS: Electron‐spray Ionization‐mass spectrometry
GC‐MS: Gas chromatography‐mass spectrometry
GPC: Gel permeation chromatography
LU: Limonene carbamate
MALDI‐MS: Matrix‐assisted laser‐desorption ionization mass spectrometry
Me‐LU: Methylated limonene carbamate
NMO: *N*‐Methylmorpholine *N*‐oxide
PLU: Poly limonene carbamate
PMA: Phosphomolybdic acid
PKIE: Primary kinetic isotope effect
pXRD: Powder X‐ray diffraction
ROP: Ring‐opening polymerization
SC‐XRD: Single‐crystal X‐ray diffraction
SKIE: Secondary kinetic isotope effect
TGA: Thermogravimetric analysis
THF: Tetrahydrofuran
TLC: Thin layer chromatography
UV–vis: Ultraviolet‐visible spectroscopy

## Introduction

Since the pioneering work of Otto Bayer on the synthesis of polyurethanes (PU), they have become one of the most widely used and best‐researched materials in the world.^[^ ^1^, ^2^ ^]^ Annual production currently accounts for 5 wt% of global polymer production and is growing steadily.^[^ ^1^, ^3^ ^]^ PUs are extensively deployed in the construction, automotive, biomedical, textile, and various other industries due to their versatile and excellent elongation, strength, and hardness properties.^[^ ^1^, ^2^, ^3^, ^4^, ^5^ ^]^ They are used as foams, adhesives, elastic fibers, and coatings, among other applications.^[^ ^1^, ^3^, ^6^ ^]^

**Scheme 1:**
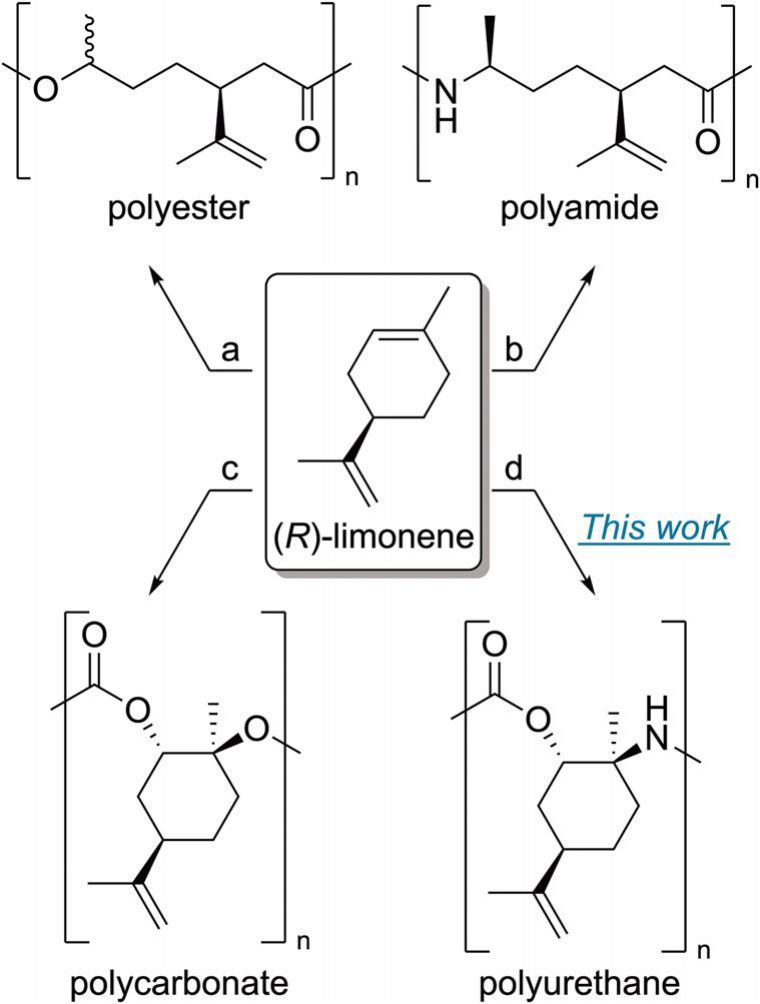
Various (*R*)‐limonene‐based polymers, all produced by ROP. a) Analogous to polycaprolactone, the monomer is obtained by Baeyer–Villiger oxidation and can be obtained from carvone or limonene.^[^ ^29^ ^]^ b) The synthesis route to limonene‐based polyamide is analogous to polycaprolactam.^[^ ^24^ ^]^ c) Bio‐based polycarbonate can be produced by copolymerizing CO_2_ and limonene oxide.^[^ ^30^ ^]^ d) The synthesis of polyurethane derived from (*R*)‐limonene via ROP is presented in this work.

Industrially, PU is still produced using the conventional manufacturing process by polyaddition of isocyanates and polyols. Here, isocyanates and phosgene, which is required for their production, represent an increased hazard potential.^[^ ^7^, ^8^, ^9^ ^]^ In terms of sustainability and safety, new strategies for phosgene‐ and isocyanate‐free PU synthesis have been developed in the last decades.^[^ ^10^, ^11^ ^]^ Most recently, cyclopropenimine‐mediated activation of CO_2_ for PU synthesis using diamines and bis‐electrophiles was introduced. This approach allows CO_2_ utilization but is limited to activated dibromo‐electrophiles.^[^ ^12^ ^]^ Another pathway is represented by the polyaddition of diamines and cyclic dicarbonates, which can be prepared from CO_2_ and epoxides. However, they often require harsh reaction conditions due to their low reactivity.^[^ ^13^, ^14^, ^15^, ^16^, ^17^, ^18^ ^]^ Further isocyanate‐free synthesis routes to PU include the polycondensation of activated linear dicarbonates and diamines or dicarbamates and diols, as well as the ring‐opening polymerization (ROP) of cyclic carbamates. Among the latter, ROP enables precise control over molecular weight and polydispersity under mild reaction conditions, leading to well‐defined polymer structures, and has the advantage that no by‐products are formed during polymerization, albeit the examples of polyurethanes via ROP are still scarce.^[^ ^13^, ^19^, ^20^, ^21^, ^22^, ^23^ ^]^ In contrast, ROP represents a well‐established method for the production of plastics such as polyamides and polyesters and enables the use of renewable raw materials.^[^ ^24^, ^25^, ^26^, ^27^ ^]^ On the way to a sustainable polymer economy, synthesizing bio‐based polymers to replace fossil‐based polymers remains one of the main challenges. Terpenes such as limonene are suitable raw materials for tackling this issue.^[^ ^28^ ^]^ While the synthesis of limonene‐based polyesters, polyamides, and polycarbonates using ROP is already known in the literature, no existing synthesis process exists for polyurethanes (Scheme 1).^[^ ^24^, ^29^, ^30^ ^]^ Herein, we present the phosgene‐ and isocyanate‐free synthesis of limonene carbamate (**LU**) starting from (*R*)‐limonene and its coordinative ROP to **PLU**. The ROP approaches of cyclic carbamates known in the literature to date use either anionic or cationic initiators.^[^ ^13^, ^19^, ^20^, ^21^, ^22^ ^]^ Furthermore, mechanistic insights are provided and the thermal properties of **PLU** are analyzed.

## Results and Discussion

### Monomer Synthesis and Polymerization

**Scheme 2:**
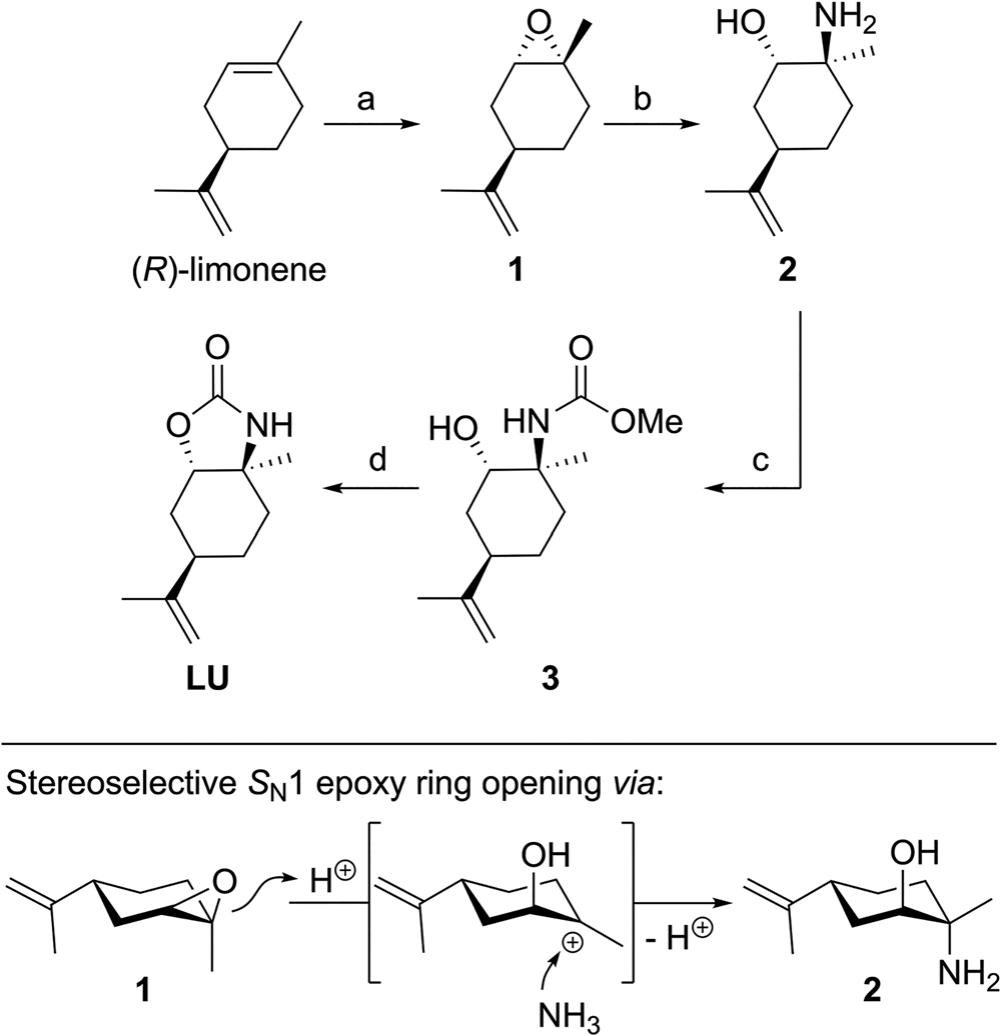
Synthetic route to monomer **LU** starting with (*R*)‐limonene. a) (*R*,*R*)‐Mn(III) Jacobsen catalyst (0.05 equiv), *m*‐CPBA (1.80 equiv) and NMO (3.00 equiv) in DCM at −40 °C for 6 h (68%). b) NH_3_ (5.00 equiv, 25% in water) refluxing for 3 days (94%). c) KO*t*‐Bu (1.05 equiv) and DMC (1.00 equiv) in toluene at 100 °C for 20 h (95%). d) KO*t*‐Bu (1.05 equiv) refluxing in THF for 16 h (83%).

To prepare the monomer **LU** on a large scale, a four‐step synthetic route starting from naturally occurring (*R*)‐limonene was established (Scheme 2). Following the procedure reported by Zhang et al., the naturally occurring terpene was stereoselectively epoxidized using Jacobsen's (*R*,*R*)‐Mn(III) catalyst, *m*‐CPBA, and NMO to afford *cis*‐limonene oxide **1** in 68% yield.^[^ ^31^ ^]^ Upon treatment with aqueous ammonia, epoxide **1** was ring‐opened in a stereoselective *S*_N_1‐type reaction, as shown in Scheme 2, to obtain amino alcohol **2** in 94% yield. Hydroxyl carbamate **3** was prepared in high yield (95%) through aminolysis of dimethyl carbonate (DMC) by amino alcohol **2**. DMC can be considered a sustainable carbonylation reagent as it can be obtained by the reaction of CO_2_ and methanol.^[^ ^32^ ^]^ The ring closure of **3** to obtain the five‐membered monomer **LU** was achieved upon refluxing in THF in the presence of potassium *tert*‐butoxide. After purification, carbamate **LU** was obtained as a colorless solid with an 83% yield. Detailed synthesis procedures and analysis of compounds **1**–**3** and **LU** can be found in the Supporting Information (see Figures S1–S22). Barbera et al. have recently reported a similar method for synthesizing *N*‐substituted cyclic carbamates, starting with a mixture of *cis*‐ and *trans*‐limonene oxide with various primary amines and DMC.^[^ ^23^ ^]^ Compared to this approach, the selective epoxidation of (*R*)‐limonene to *cis*‐limonene oxide **1**, as presented here, allows the formation of a selective regioisomer without tedious separation by column chromatography.

Alternative attempts to convert limonene oxide **1** into **LU** applying a one‐step route using potassium cyanate or the reaction of amino alcohol **2** with CO_2_ in the presence of cerium(IV) oxide did not result in **LU** at all or only in low selectivity (for further details, see Supporting Information). However, refluxing epoxide **1** for 6 days with potassium cyanate in methanol afforded the formation of **3** in 24% yield, determined by GC.

**Figure 1:**
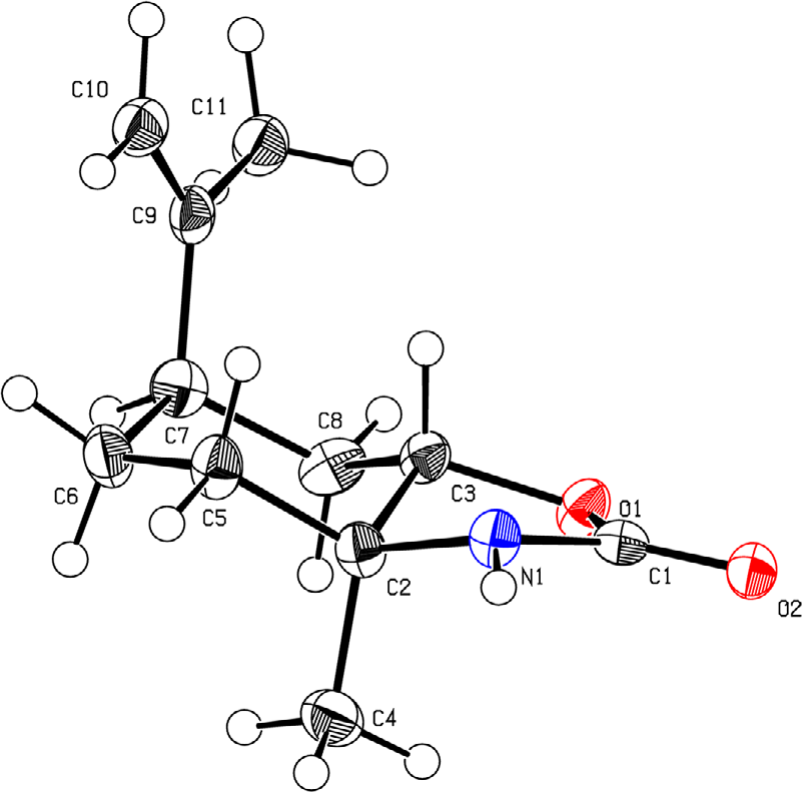
ORTEP‐style representation (50% probability level) of **LU**. Color code: white = hydrogen, grey = carbon, blue = nitrogen, and red = oxygen. Selected dihedral angles (°): N1–C2–C3–O1 39.62(16)°, C1–N1–C2–C3 36.09(18)°, C1–O1–C3–C2 31.73(18)°. The highly strained five‐membered carbamate ring occupies the equatorial position, while the methyl (C4) and isopropenyl (C9–C11) fragments are located in the axial position, standing *trans* to each other. Single crystals suitable for X‐ray crystallography were grown via slow evaporation from a solution of **LU** in ethyl acetate/*n*‐hexane (1:1) at −32 °C. CCDC: 2392925.

The successful synthesis of **LU** was confirmed by elemental analysis and ESI‐MS in addition to NMR spectroscopy. Furthermore, single crystals suitable for X‐ray crystallography were obtained from a solution of **LU** in ethyl acetate/*n*‐hexane (1:1) at −32 °C, which allowed the molecular structure to be determined unequivocally (Figure 1). The isopropenyl (C9–C11) and methyl (C4) moieties are attached to the cyclohexyl core in the axial position, while N1 and O1 of the five‐membered carbamate ring occupy the equatorial positions. As described above, in the synthesis of **LU**, the epoxide opening of **1** proceeds in a stereoselective *S*_N_1‐type reaction, which is confirmed by the fact that N1 is attached to the tertiary carbon C2. This finding is in agreement with the stereoselective ring opening of *cis*‐limonene oxide **1** by sodium azide or primary and secondary amines previously described in the literature.^[^ ^33^, ^34^, ^35^, ^36^ ^]^ The cyclohexyl core structure of **LU** is slightly bent and deviates somewhat from the ideal chair conformation, as evidenced by the bond angles within the six‐membered ring. The bond angles C3–C8–C7 and C5–C6–C7 at 104.99(15)° and 115.37(15)° deviate most strongly from the tetrahedral angle (109.5°). Comparison of the dihedral angles of the five‐membered carbamate ring in **LU** with those of 1,3‐oxazolidin‐2‐one indicates a high ring strain.^[^ ^37^ ^]^ While 1,3‐oxazolidin‐2‐one is approximately planar, the dihedral angles of **LU** for N1–C2–C3–O1 are 39.62(16)°, C1–N1–C2–C3 are 36.09(18)°, and C1–O1–C3–C2 are 31.73(18)°.

**Scheme 3:**
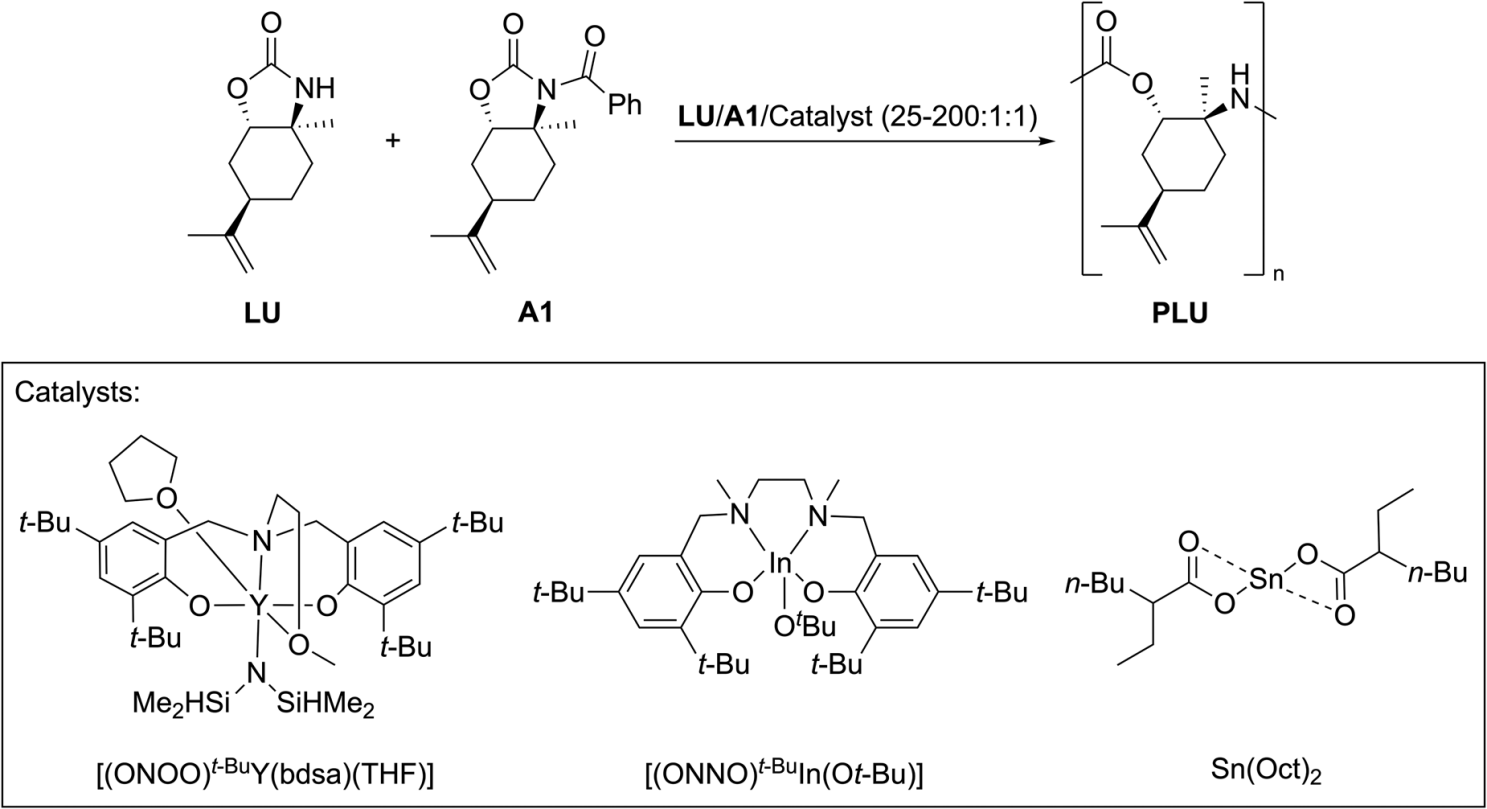
Potential catalysts for the ring‐opening polymerization of **LU**, which were all reported to be productive for either polyesters or polyamides synthesized via ROP.

The axial position of the sterically demanding isopropenyl moiety, the bent cyclohexyl core, and the highly strained carbamate ring serve as a thermodynamic traction to polymerize **LU** in a ring‐opening manner. The decomposition temperature of 224 °C and the melting point of 104 °C of **LU** allow a broad temperature range for bulk and solution polymerization. For anionic ROP of cyclic carbamates, Thomas et al. have proposed a mechanism analogous to polyamides, in which initiation occurs by deprotonation of the monomer followed by addition to an activator. Typically, activators are acylated monomers or acylating agents themselves.^[^ ^13^, ^38^ ^]^ In order to facilitate the polymerization of **LU**, the activator **A1** was synthesized by acylation of **LU** with benzoyl chloride (see Figures S23–S28). The imide moiety in **A1** is intended to increase the electrophilicity of the carbonyl moiety of the carbamate ring, which should alleviate the ROP initiation. Having activator **A1** and monomer **LU** at hand, potential initiators NaH, *n*‐BuLi,^[^ ^13^ ^]^ as well as organometallic catalysts [(ONOO)*^t^* ^‐Bu^Y(bdsa)(THF)],^[^ ^39^ ^]^ [(ONNO)*^t^* ^‐Bu^In(O*t*‐Bu)]^[^ ^40^ ^]^ and Sn(Oct)_2_^[^ ^26^, ^27^, ^41^ ^]^ were selected for initial polymerization approaches (Scheme 3). Since carbamates combine an ester and amide functionality, catalysts with high reactivity for such moieties were chosen for the polymerization, as we expected a similar polymerization mechanism for **LU**. Yttrium tris(*iso*‐propoxide) was reported to polymerize cyclic carbamates before. However, only low molecular weights were obtained.^[^ ^13^ ^]^ Moreover, yttrium tris(*iso*‐propoxide) was applied for several chain growth polymerization systems like *ε*‐caprolactone, lactide, and cyclic carbonates, demonstrating its versatility.^[^ ^42^, ^43^, ^44^ ^]^ Therefore, the yttrium‐based complex [(ONOO)*^t^* ^‐Bu^Y(bdsa)(THF)] was considered a promising catalyst since it is highly active in the ROP of various cyclic esters.^[^ ^25^, ^39^ ^]^ [(ONNO)*^t^* ^‐Bu^In(O*t*‐Bu)] was also used for the ROP of a broad range of esters like *β*‐butyrolactone, *ε*‐caprolactone, and *ε*‐decalactone.^[^ ^40^ ^]^ The catalyst Sn(Oct)_2_ was taken into account; it has been shown to be effective not only in the ROP of polyesters and polyamides but also in copolymerization and industrially on a large scale.^[^ ^26^, ^27^, ^41^ ^]^

For initial polymerization experiments, a **LU**/**A1**/catalyst ratio of 50:1:1 and a reaction time of 20 h were set. An aliquot of the respective reaction solution was taken and quenched with wet deuterated chloroform. The conversion (*X* **_LU_**), average molar weight (*M*_n_), and polydispersity (*Đ*) were analyzed by ^1^H NMR (see Figures S46 and S47) and GPC measurements, respectively. Polymerization in bulk, using NaH as an initiator, led to the decomposition of the monomer **LU** (Table S1, entry 1). Before, NaH was applied as a base in the ring closure of **3** to **LU**, which also led to the decomposition of **LU** over time and the formation of various unselective species. Potential decomposition pathways in the presence of NaH could be either the decarboxylation or the reduction of **LU**. For this reason, NaH was not considered for any further polymerization attempts. The reaction conditions for *n*‐BuLi as initiator were based on the work published by Thomas et al. on the ROP of cyclic carbamates (Table S1, entry 2).^[^ ^13^ ^]^ However, this measure yielded only 5% conversion, and no oligomeric species were obtained. The result could not be improved by varying the temperature or longer reaction times. Similarly, employing crown ether and potassium *tert*‐butoxide to avoid the formation of strong ion pairs did not produce higher *M*_n_ (Table S1, entries 3 and 4). In line with this observation, Barbera et al. recently reported on the anionic ROP of limonene‐based carbamates, which also led to the formation of oligomers with an *M*_n_ of 1.2 kg mol^−1^ only.^[^ ^23^ ^]^ The catalysts [(ONOO)*^t^* ^‐Bu^Y(bdsa)(THF)] and [(ONNO)*^t^* ^‐Bu^In(O*t*‐Bu)] were investigated in THF and toluene, whereby the reaction temperatures were adjusted to the boiling point of the respective solvent. While the yttrium‐based catalyst in toluene achieves a conversion of 43% and an average molar weight *M*_n_ of 1.1 kg mol^−1^, both values decrease to 37% and 0.9 kg mol^−1^, respectively, for polymerization in THF at a reaction temperature of 60 °C (Table S1, entries 5 and 6). Furthermore, the polydispersity *Đ* widens from 1.7 to 2.9, and a bimodal distribution is obtained instead of a monomodal distribution. By increasing the reaction temperature to 100 °C in toluene, *M*_n_ and *Đ* remain almost unchanged, but the conversion decreases to 18%, and a bimodal distribution is obtained. Catalyst degradation at elevated temperatures is a possible explanation for this observation (Table S1, entry 8). When applying the indium‐based catalyst, **LU** remained unmodified, and no conversion was observed (Table S1, entries 9 and 10). Attempts to polymerize **LU** in the absence of **A1** had no notable effect for [(ONOO)*^t^* ^‐Bu^Y(bdsa)(THF)], whereas oligomers with an *M*_n_ of 3.0 kg mol^−1^ were observed by using [(ONNO)*^t^* ^‐Bu^In(O*t*‐Bu)] (Table S1, entries 7 and 11). Possibly, **A1** coordinates as a bidentate ligand (vide infra), thereby inhibiting polymerization.

This drastically changed when switching to Sn(Oct)_2_ as a catalyst. The tin‐based catalyst was applied in THF, toluene, and dioxane at temperatures above 60 °C, as elevated temperatures were reported to be crucial for Sn(Oct)_2_ in the ROP of polyesters and polyamides before.^[^ ^27^, ^45^, ^46^ ^]^ In agreement with this, in THF and toluene no conversion was observed at 60 °C, while increasing the temperature to 80 °C resulted in 73% conversion after 20 h and 91% conversion after 15 h at 100 °C (Table 1, entries 1–5). The *M*_n_ increased from 4.7 to 9.9 kg mol^−1^ with temperature, whereas *Đ* broadened from 1.7 to 1.9. Notably, at 100 °C, a DP of 49 was obtained, matching the monomer‐to‐catalyst ratio very well. A potential explanation for the more efficient **LU** activation via the coordinative method using Sn(Oct)_2_ compared to the anionic initiators could be the lower strength of the ion pair, thereby not inhibiting the propagation step and thus representing a milder approach. In contrast to the yttrium‐ and indium‐based catalysts bearing tetradentate ligands, the metal center of Sn(Oct)_2_ is more readily accessible for monomer coordination due to the larger ionic radius as well as for steric reasons, providing a potential explanation for higher activity.

**Table 1:** Sn(Oct)_2_ catalyzed ROP of **LU** (see Table S1 for screening of further catalysts and initiators).

Entry	Catalyst	**LU**/**A1**/Sn(Oct)_2_	Solvent^a)^	*t*_Pol_ (h)	*T*_Pol_ (°C)	*X*_**LU**_ ^b)^ (%)	*M*_n,GPC_ ^c)^ (kg mol^−1^)	*M*_n,NMR_ ^b)^ (kg mol^−1^)	DP^c)^	*Đ* ^c)^
1	Sn(Oct)_2_	50:1:1	THF	20	60	–	–	–	–	–
2	Sn(Oct)_2_	50:0:1	THF	20	60	–	–	–	–	–
3	Sn(Oct)_2_	50:1:1	Toluene	20	60	–	–	–	–	–
4	Sn(Oct)_2_	50:1:1	Toluene	20	80	73	4.7	4.9	24	1.7
5	Sn(Oct)_2_	50:1:1	Toluene	15	100	91	9.9	10.3	49	1.9
6	Sn(Oct)_2_	50:5:1	Toluene	15	100	90	9.3	9.8	46	1.7
7	Sn(Oct)_2_	50:1:5	Toluene	10	100	93	3.3	10.2	17	2.1
8	Sn(Oct)_2_	25:1:1	Toluene	10	100	89	5.3	5.4	26	1.8
9	Sn(Oct)_2_	100:1:1	Toluene	20	100	90	12.3	12.7	63	1.9
10	Sn(Oct)_2_	200:1:1	Toluene	20	100	81	16.0	16.5	82	1.5
11	Sn(Oct)_2_	50:0:1	Toluene	15	100	72	5.2	–	27	2.9
12	Sn(Oct)_2_	50:1:1	Dioxane	15	100	77	6.1	6.8	30	2.0
13	Sn(Oct)_2_	50:0:1	Dioxane	15	100	73	4.8	–	25	2.4

^a)^ Reaction conditions: [**LU**] =  1 M.

^b)^ Conversion of **LU** (*X* **_LU_**) and number‐average molar mass (*M*_n,NMR_) related to the benzoyl end group of **A1** determined via ^1^H NMR in CDCl_3_ (see Figures S46 and S47).

^c)^ Number‐average molar mass (*M*_n,GPC_), degree of polymerization (DP), and polydispersity index (*Đ *= *M*_w_/*M*_n_) determined via gel permeation chromatography (GPC) in DMF with 2.096 g L^−1^ LiBr added at 30 °C referenced to poly(methylmethacrylate) calibration standards.

Using a fivefold amount of activator **A1** gave a slightly narrower distribution *Đ* of 1.7, while the conversion and *M*_n_ remained comparable (Table 1, entry 6). In contrast, applying a fivefold amount of catalyst decreased *M*_n_ to 3.3 kg mol^−1^ as determined by GPC measurements (Table 1, entry 7). This indicates that Sn(Oct)_2_ initiates the polymerization, and the monomer‐to‐catalyst ratio determines the chain length. The deviation of *M*_n_ determined by ^1^H NMR analysis via the benzoyl end group and the slightly increased polydispersity suggest initiation of the polymer chains occurred partially without activator **A1** (comparison with Table 1, entry 11 vide infra). Lowering the **LU**/**A1**/Sn(Oct)_2_ ratio to 25:1:1 resulted in a conversion of 89% and an *M*_n_ of 5.3 kg mol^−1^ after 10 h (Table 1, entry 8). With a polydispersity *Đ* of 1.8, the DP is in the range of the monomer‐to‐catalyst ratio, and *M*_n_ determined by GPC and NMR are also close to each other. For the **LU**/**A1**/Sn(Oct)_2_ ratio of 100:1:1, the conversion and polydispersity *Đ* remain almost unchanged, whereas the *M*_n_ increased to 12.3 kg mol^−1^ (Table 1, entry 9). In the case of the 200:1:1 ratio, only 81% conversion was achieved, but an even higher *M*_n_ of 16.0 kg mol^−1^ equal to a DP of 82, was obtained after 20 h (Table 1, entry 10). Moreover, in comparison to the polymerization attempts using lower monomer ratios, the polydispersity *Đ* decreased to 1.5.

**Scheme 4:**
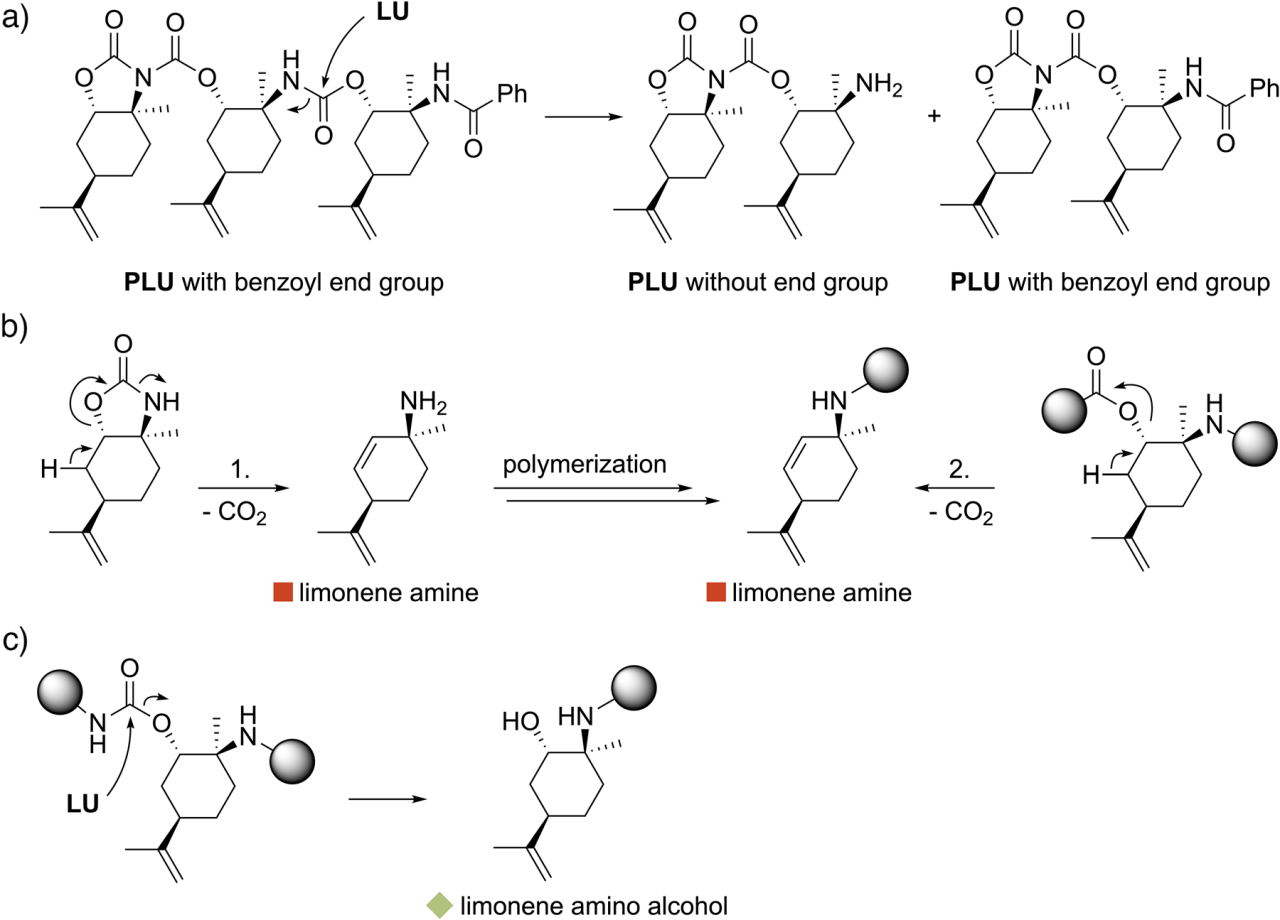
Possible pathways accounting for the end groups of minor mass populations in the ESI‐MS of **PLU** oligomers. a) Transamidation within the polymer backbone of **PLU** leading to two active imide‐type chain ends, whereby the benzoyl end group of **A1** is only present in one. b) CO_2_ elimination of **LU** (1.) resulting in limonene amine with an *m*/*z* of 151.14 (red square, compare to Figure 2b) as initiator in the ROP of **LU**. Acetate elimination followed by decarboxylation within a polymer chain (2.) also leads to the limonene amine fragment. c) Transesterification resulting in a limonene amino alcohol fragment with an *m*/*z* of 169.15 (green square, compare to Figure 2b).

Presumably, side reactions such as intra‐ and intermolecular transamidation, promoted at elevated temperatures, are suppressed until this point, as no complete conversion has yet been reached.^[^ ^45^, ^46^ ^]^ We surmise a nonlinear increase in *M*_n_ and DP with the higher **LU**‐to‐catalyst ratios, as the formed polymers already precipitate out of the reaction solution at a ratio of 100:1. Solubility issues in the ROP of cyclic carbamates have been previously reported by Thomas et al.^[^ ^13^ ^]^ Another reason could be scrambling of the polymer chains by transamidation (see Scheme 4a). Switching to dioxane as a more polar solvent prevented precipitation of the polymers, albeit the conversion of **LU** decreased to 77%, and a lower *M*_n_ of 6.1 kg mol^−1^ was obtained (Table 1, entry 12). As previously reported for the ROP of *ε*‐caprolactone in dioxane with Sn(Oct)_2_, competitive coordination of dioxane to the catalyst could be a possible reason for elongated reaction times and lower conversion compared to the polymerizations in toluene.^[^ ^45^ ^]^ All tin‐catalyzed polymerizations listed in Table 1 show a monomodal distribution, and *Đ* is within the range of ROP‐derived polyesters and polyamides in the presence of Sn(Oct)_2_ except for entries 11 and 13 (see Figure S48 for comparison of entries 9 and 11).^[^ ^26^, ^27^, ^47^ ^]^ Polymerizing **LU** in the absence of **A1** led to a drastic broadening of *Đ* to 2.9 and a reduced *M*_n_ of 5.2 kg mol^−1^ and conversion of 72% (Table 1, entry 11). The polymerization in dioxane resulted in a comparable conversion and *M*_n_ of 73% and 4.8 kg mol^−1^, respectively, and a slightly narrower *Đ* of 2.4 (Table 1, entry 13). These observations could be due to facilitated initiation by the reactive imide moiety when using **A1** as activator, prompting us to analyze the resulting polymers by ESI‐MS for end group analysis to better understand the role of **A1**.

**Figure 2:**
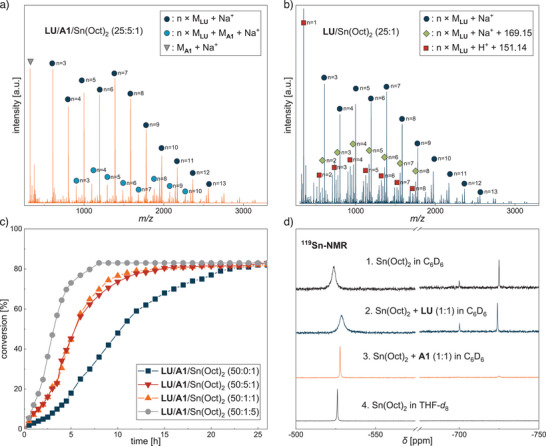
a) ESI‐MS of the crude **LU** oligomers (*M*_n_ = 1.5 kg mol^−1^) using activator **A1** [**LU**/**A1**/Sn(Oct)_2_ 25:5:1] directly from the reaction mixture after 3 h. The mass population with **A1** as the end group (light blue circles) is less pronounced than the oligomer chains consisting only of **LU** (dark blue circles). b) ESI‐MS of the crude **LU** oligomers (*M*_n_ = 1.3 kg mol^−1^) obtained from the polymerization without using an activator [**LU**/Sn(Oct)_2_ 25:1] directly from the reaction mixture after 3 h. **LU** initiates itself (dark blue circles), but compared to a, more side reactions like transesterification (green squares) and CO_2_ elimination (red squares) take place. c) Kinetic investigation of the **LU** polymerization with varying **LU**/**A1**/Sn(Oct)_2_ ratio at 100 °C in deuterated toluene (1 M). Higher catalyst loading leads to a significant acceleration of the polymerization (grey circles). The use of **A1** as an activator (orange triangles) results in faster polymerization compared to without activator (blue squares), whereby no significant influence was observed with higher amounts of **A1** (red triangles). d) ^119^Sn NMR spectra of Sn(Oct)_2_ 1.) pure in deuterated benzene, 2.) with 1.00 equiv **LU** in deuterated benzene, 3.) with 1.00 equiv **A1** in deuterated benzene, and 4.) pure in deuterated THF.

Comparing the ESI‐MS spectra of the crude oligomer samples with and without the activator **A1** (Figure 2a and b, respectively), it is noticeable that the main series of both oligomers solely consists of **LU** repeating units without any initiation group (dark blue circles). This suggests the polymerization proceeds by self‐initiation of **LU** in the absence of **A1**. While the mass population of the benzoyl‐terminated oligomers (light blue circles) in the presence of **A1** is less pronounced, self‐initiation of **LU** is not the only pathway leading to the main series solely consisting of **LU** repeating units. The occurrence of the mass population without the benzoyl end group of **A1** in Figure 2a can also be explained by transamidation. The acyl substitution upon nucleophilic attack of **LU** cleaves the polymer chain, resulting in two active chains terminated by the imide fragment on the one side and on the other side either with or without the benzoyl end group (Scheme 4a). Both polymer chains can continue to grow, explaining the slightly increased polydispersities *Đ* of about 2.0. Furthermore, the transamidation might account for the nonlinear increase in *M*_n_ from 9.9 to 12.3 kg mol^−1^ with an **LU**/**A1**/Sn(Oct)_2_ ratio of 50:1:1 and 100:1:1, respectively (Table 1, entries 12 and 16). With increasing chain length, the probability of chain scission by transamidation instead of chain growth by ring opening also rises. In addition, the ESI‐MS spectra comparison indicates the reduced occurrence of side reactions using **A1**. The facilitated polymerization initiation via the imide moiety of **A1** explains this finding (vide infra). In Figure 2b, the green and red squares mark the series caused by side reactions. Both were not found in the ESI‐MS spectrum using **A1** and can be explained by CO_2_ elimination or transesterification (Figure 2a). Scheme 4b shows two possible pathways for forming the end group with the *m*/*z* of 151.14, which is assigned to limonene amine (red squares). CO_2_ elimination of **LU** (1.) and acetate elimination with subsequent decarboxylation in the polymer chain (2.) are conceivable routes for forming the end group. The end group with the *m*/*z* of 169.15 is assigned to limonene amino alcohol (green squares) and can be explained by transesterification (Scheme 4c). It must be noted that in the MALDI‐MS spectra of the precipitated **PLU** samples of entries 9 and 11 from Table 1, the mass populations caused by CO_2_ elimination and transesterification are more pronounced compared to the series consisting solely of monomer repeating units (see Figure S49). Increased side reactions at higher conversions as the polymerization proceeds can explain this finding.

**Figure 3:**
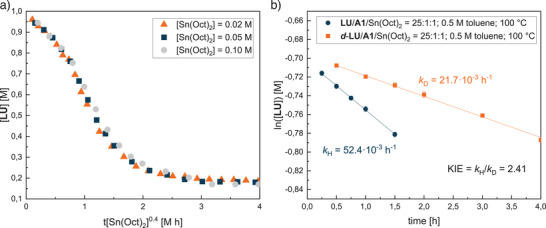
a) Plot of [**LU**] against catalyst t[Sn(Oct)_2_]*^n^*, *n* = 0.4; fitting yields a 0.4^th^ order in catalyst concentration. Reaction conditions: [**LU**] = 1.00 M, [**A1**] = 0.02 M, 100 °C in toluene. b) Plot of *k*_H_ (blue curve) and *k*_D_ (orange curve) observed for the polymerizations of **LU** and ***d***‐**LU** (0.5 M in toluene at 100 °C; **LU**/**A1**/Sn(Oct)_2_ = 25:1:1), respectively, revealing a primary KIE of 2.41.

The ^1^H NMR kinetic studies with varying **LU**/**A1**/Sn(Oct)_2_ ratios demonstrate a further influence of activator **A1** (Figure 2c). While 82–85% conversion was reached for each polymerization attempt after 26 h, the reaction time to reach equilibration differed drastically. The sigmoidal curves indicate a multistage polymerization mechanism wherein the active species is first formed in the initiation phase, and the reaction halts when the equilibrium of polymerization/depolymerization is reached. The slightly lower conversion compared to the results in Table 1 can be explained by the J‐Young NMR tube setup, which influences the heat transfer and prevents stirring. Applying an **LU**/**A1**/Sn(Oct)_2_ ratio of 50:1:1, it takes about 15 h until a conversion of 82% is reached (orange triangles). The reaction order for monomer **LU** was determined via an ln(*v*_0_)–ln([**LU**]) plot using the initial rates for [**LU**] = 0.5, 0.75, and 1.00 M (see Figure S51). At fivefold catalyst loading, the reaction time was almost halved, and the **LU** conversion stopped after around 8 h (grey circles). The plot of [**LU**] against catalyst t[Sn(Oct)_2_]*^n^* suggests a 0.4^th^ reaction order in catalyst concentration, indicating a multistage mechanism with equilibria (see Figure 3a).^[^ ^48^ ^]^ By using a fivefold amount of **A1**, the curve is almost congruent to one equivalent (red triangles). This demonstrates that by increasing the **A1** amount, the kinetics cannot be further accelerated and results in a zero‐order reaction with respect to the activator (see Figure S52). In contrast, polymerization in the absence of **A1** proceeds more slowly (blue squares), and it takes 25 h until equilibration is reached. Again, the facilitated polymerization initiation by the imide moiety of **A1** explains this finding. The combination of ESI‐MS analysis and kinetic studies suggests that **A1** not only accelerates the polymerization but also suppresses side reactions.

To clarify the influence of activator **A1** on Sn(Oct)_2_, stoichiometric ^119^Sn NMR experiments were carried out (Figure 2d). In addition to the broad signal at *δ *= −523.82 ppm, the spectrum of the pure tin catalyst in deuterated benzene shows further signals at *δ *= −699.98 and −724.95 ppm. This can be explained by the low oxidation state and coordination number of Sn(Oct)_2_, which leads to agglomerate formation in nonpolar solvents.^[^ ^49^, ^50^, ^51^ ^]^ Similar shifts were obtained for the 1:1 mixture of **LU** and Sn(Oct)_2_. However, adding activator **A1** equimolar to the tin catalyst, the two upfield‐shifted signals almost completely disappear, and a sharp signal is obtained at *δ *= −527.73 ppm. We assume that **A1** coordinates as a weak ligand via the carbonyl moieties to the tin species, breaking its oligomeric structures and thus making the catalyst more accessible for the coordination of **LU**. To confirm this assumption, pure Sn(Oct)_2_ was measured in deuterated THF as a coordinating solvent, leading to a somewhat comparable spectrum suggesting the coordination of **A1** to Sn(Oct)_2_.

**Figure 4:**
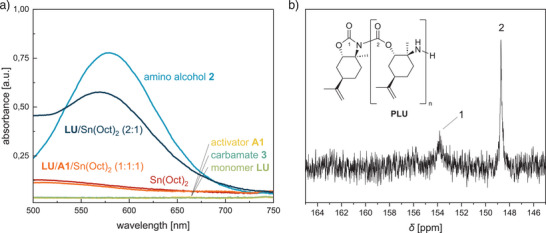
End group and tacticity analysis of **PLU**. a) UV–vis spectra of various **LU**‐derived compounds after being exposed to 2.00 equiv ninhydrin in ethanol. b) The carbonyl region of the ^13^C NMR spectrum shows a weak signal for the imide end group in addition to the urethane signal.

Further stoichiometric experiments with **LU**, **A1**, and Sn(Oct)_2_ were carried out to determine the initiation mechanism of **LU** and the end group of **PLU** (see Figure S53). While the reaction of **LU**/**A1**/Sn(Oct)_2_ (1:1:1) again leads to the formation of an imide unit and a benzoyl end group, analogous to the ROP of polyamides, the self‐initiation of **LU**/Sn(Oct)_2_ (2:1) also results in the formation of an imide unit, albeit with an amine end group. To detect the amine group, the reaction products were quenched with 2.00 equiv ninhydrin in ethanol and subsequently analyzed by UV–vis spectroscopy. Ninhydrin reacts selectively with primary amines, resulting in Ruhemann's purple color of the reaction solution with an absorption maximum of 570–580 nm.^[^ ^52^ ^]^ Only for the ring‐opening product of **LU**/Sn(Oct)_2_ (2:1) and amino alcohol **2** as a reference was an absorption maximum measured in this range (Figure 4a, dark and light blue curves, respectively). This result confirms the amine end group in the absence of **A1** and reveals that the ring opening of **LU** occurs through the cleavage of the amide moiety in the carbamate ring. We suspect the free amine end group contributes to the broadening of the polydispersity via transamidation analogous to the chain scission shown in Scheme 4a, which matches the results in the absence of **A1** (Table 1, entries 11 and 13). To exclude potential interference from urethane units or the catalyst, carbamate **3** (dark green curve), monomer **LU** (light green curve), activator **A1** (yellow curve), and Sn(Oct)_2_ (red curve) were also treated with 2.00 equiv ninhydrin, which did not result in a color change in the solutions. Likewise, no free amine was detected for the ring‐opening product of **LU**/**A1**/Sn(Oct)_2_ (1:1:1), matching the benzoyl end group.

Consequently, **PLU** possesses a benzoyl or amine end group depending on the use of **A1**, which is also confirmed by the ^13^C NMR spectra of the differently initiated polymers. For example, the spectrum of **PLU** without **A1** exhibits only two signals in the carbonyl region at *δ *= 153.81 and 148.72 ppm, which are assigned to the imide end group and the urethane units, respectively (Figure 4b). The correlations in the ^1^H^15^N HMBC spectrum also match these chemical shifts, confirming not only the secondary amines of the **LU** repeating units at *δ *= 110.04 ppm but also the imide and amine end groups at *δ *= 147.75 and 48.56 ppm, respectively (see Figure S45). In contrast, the signals of the benzoyl end group are also detected for the **PLU** sample with **A1** at *δ *= 160.45 ppm (carbonyl moiety) and in the range of *δ *= 111–112 ppm (phenyl moiety) in the ^13^C NMR spectra (see Figure S44). In both cases, no further signals in addition to the urethane repeating unit indicating carbonate or urea linkages are observed. As a result, the ROP of **LU** proceeds selectively via cleavage of the amide moiety and leads to a high tacticity of **PLU**.

To gain a deeper understanding of the chain growth propagation and the interaction of **LU** with the catalyst, the *N*–*H* moiety was blocked by methylation (see Figures S34–S38). As shown in Table S2, attempts to polymerize the monomer **Me**‐**LU** under the same reaction conditions as **LU** failed (see Figures S54 and S55). This suggests an essential role of the *N*–*H* moiety of **LU** by proton transfer and opening the carbamate ring of the following monomer via the amide moiety. This finding matches the stoichiometric experiments with ninhydrin (vide supra).

**LU** was deuterated for kinetic isotope experiments to obtain further information on the proton transfer of the *N*–*H* moiety (see Figures S29–S33). The kinetic isotope effect (KIE = *k*_H_/*k*_D_) was investigated by determining the rate constants *k* of the polymerizations of **LU** and ***d***‐**LU** in toluene (0.5 M). The observed values of *k*_H_ = 52.4 · 10^−3^ h^−1^ and *k*_D_ = 21.7 · 10^−3^ h^−1^ result in a primary KIE of 2.41 (PKIE ≫ 1), suggesting a bond breaking of the *N*–*H* moiety of **LU** (see Figure 3b). A secondary KIE would occur when the internal vibrations of the system are affected (SKIE ∼ 1.1–1.2 or, if inverted, ∼ 0.8–0.9).^[^ ^53^, ^54^, ^55^, ^56^, ^57^ ^]^ This finding further provides a possible explanation for the unsuccessful polymerizations of **Me**‐**LU** and the *N*‐alkylated carbamates previously described by Barbera et al.^[^ ^23^ ^]^

**Scheme 5:**
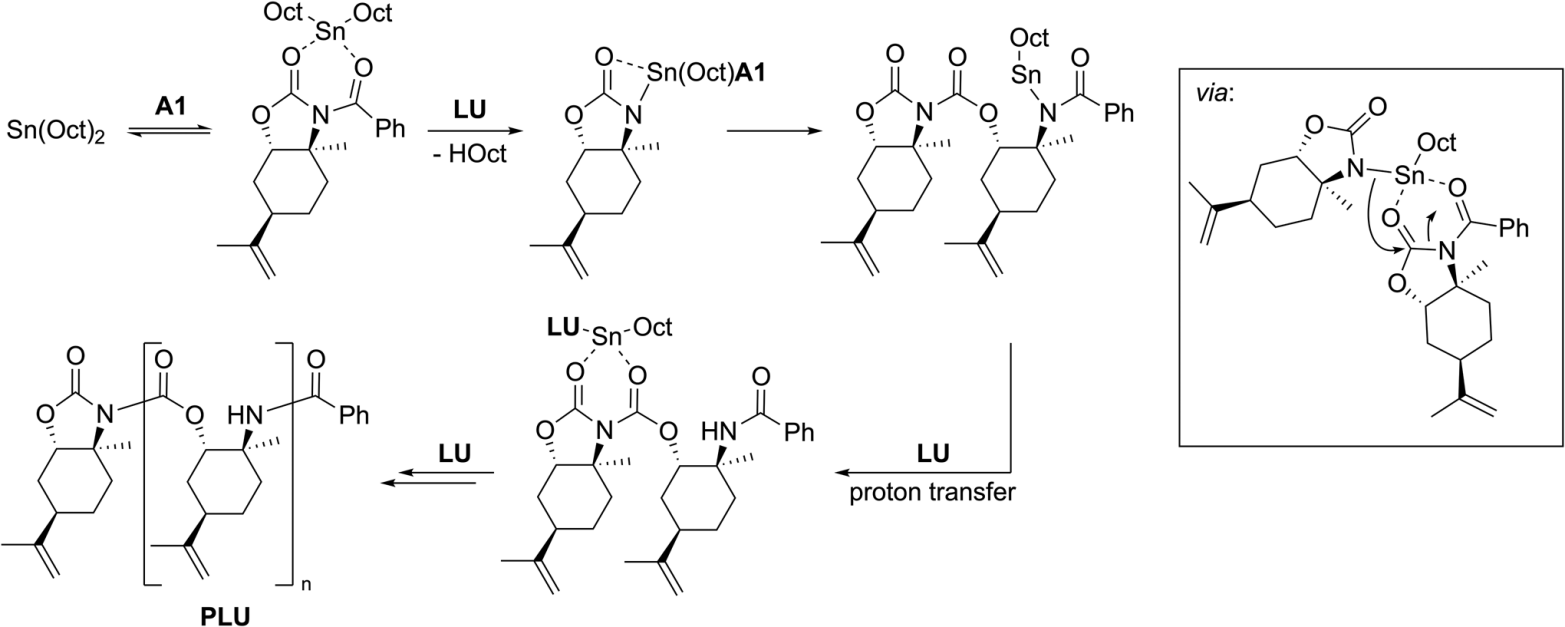
Proposed polymerization mechanism in the presence of **A1** based on experimental data.

Scheme 5 illustrates a plausible mechanism for the initiation and polymerization of **LU** based on the results from ESI‐MS analysis, kinetic studies, ^119^Sn NMR experiments, the end group analysis using ninhydrin, ^13^C NMR spectroscopy, the unsuccessful polymerization attempts of **Me**‐**LU**, and the KIE experiments using ***d***‐**LU**. According to the stoichiometric ^119^Sn NMR and kinetic studies, the polymerization of **LU** in the presence of **A1** is presumably initiated by coordinating the imide moiety to Sn(Oct)_2_. Followed by octoic acid cleavage, **LU** coordinates via the amide moiety to the Sn catalyst. This step is deduced from the polymerizations with varying Sn(Oct)_2_ equivalents, the unsuccessful polymerization of **Me**‐**LU**, and the KIE experiments using **d**‐**LU**. The benzoyl and imide end groups are formed by the ring opening of **A1**, completing the initiation of the polymer chain. We base this step on the analysis of **PLU** by ESI‐MS, ^13^C NMR, and UV–vis spectroscopy of the stoichiometric experiments of **LU**, **A1**, and Sn(Oct)_2_ with ninhydrin. Subsequently, the propagation occurs by repeated ring opening of the imide moiety via the amide moiety of **LU**. The ^13^C NMR measurements of **PLU** also reveal the selective formation of urethane repeating units.

In summary, the presented coordinative ROP of **LU** provides a novel method to access polyurethanes, allowing for higher *M*_n_ than reported for anionic ROP of similar cyclic carbamates before.^[^ ^13^, ^22^ ^]^ Compared to isocyanate‐free step‐growth strategies based on (*R*)‐limonene, such as polyaddition of cyclic dicarbonates and diamines, *M*_n_ values in the same range were obtained.^[^ ^18^, ^58^, ^59^ ^]^

### Chemical Circularity and Thermal Properties of PLU

**Figure 5:**
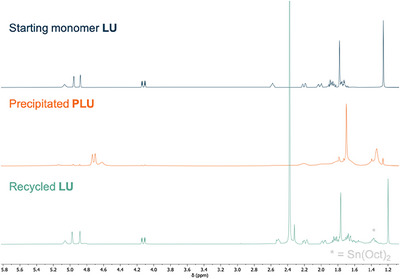
Stacked NMR spectra of starting monomer **LU**, the purified **PLU** sample (*M*_n_ = 5.3 kg mol^−1^), and the crude recovered **LU** in mesitylene after depolymerization at 140 °C using Sn(Oct)_2_ as catalyst.

To address the end‐of‐life properties of **PLU**, its chemical recyclability was investigated in a simple sublimation setup in the presence of 10 mol% ZnCl_2_ or phosphomolybdic acid (PMA) as catalysts due to their effectiveness in depolymerizing polyamides and polyesters.^[^ ^60^ ^]^ While no product could be isolated by heating **PLU** with PMA up to 200 °C and only causing a black coloration of the polymer, traces of **LU** were recovered in a product mixture after 2 h using ZnCl_2_ (see Figures S57 and 58). Compared to the reported chemical recycling of polycarbonates, the formation of the corresponding aziridine under CO_2_ elimination was not observed.^[^ ^61^ ^]^ We surmise an unselective degradation and consequently low yield due to the high required temperature close to the decomposition temperature of **LU** (224 °C). This changed drastically when **PLU** was depolymerized under milder conditions in a 0.1 M mesitylene solution. Using 10 mol% of Sn(Oct)_2_ as a catalyst, **PLU** (*M*_n_ = 5.3 kg mol^−1^) was converted back to **LU** in high purity in almost quantitative yield within 2 h at 140 °C (Figures 5 and S56).

Furthermore, the hydrolytic degradation of **PLU** (*M*_n,0_ = 12.3 kg mol^−1^) in 5 M aqueous potassium hydroxide solution at 70 °C was studied for 7 days and analyzed by GPC. As shown in Figure S59, *M*_n_ decreased to about 90%, interestingly more than previously reported for strictly alternating polyesters under the same reaction conditions by Williams et al.^[^ ^62^ ^]^ The resulting suspension was filtered and washed with chloroform to analyze the low molecular weight degradation products by GC‐MS. Besides monomer **LU** and an unknown isomer thereof, **LU**‐dimers resulting from CO_2_ elimination were detected (see Figure S60).

In many applications polyurethanes are employed due to their resistance to high temperatures; therefore, the thermal properties of the synthesized **PLU** were investigated by thermogravimetric analysis (TGA), differential scanning calorimetry (DSC), rheology, and powder X‐ray diffraction (pXRD).

**Figure 6:**
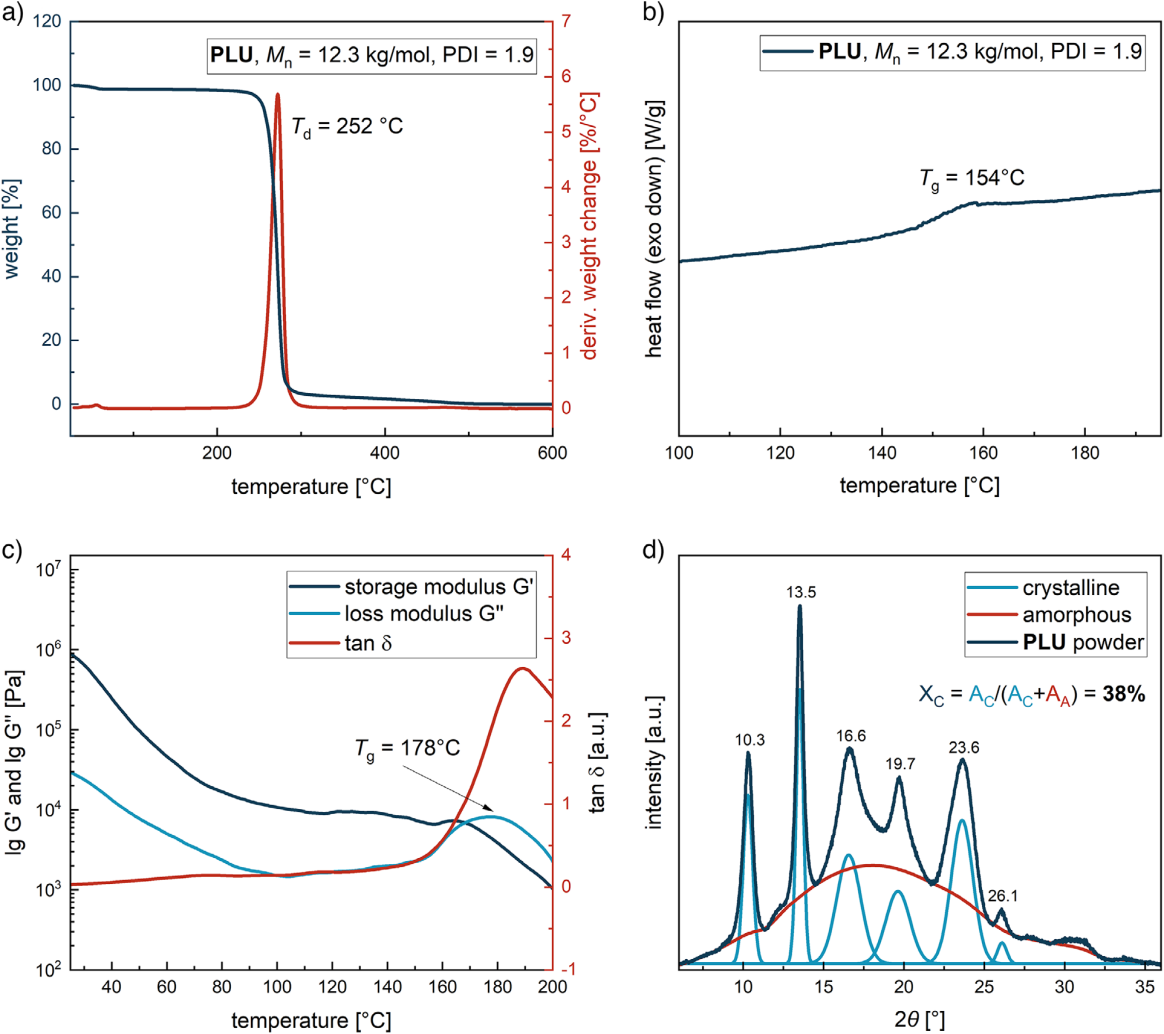
a) TGA thermogram (10 K min^−1^) of **PLU** shows a *T*_d_ of 252 °C. b) DSC thermogram (20 K min^−1^) of the second heating scan for **PLU** indicates a *T*_g_ of 154 °C. Before thermal analyses, **PLU** sample was dried at 80 °C for 40 h under vacuum. c) Rheological thermomechanical profile at fixed frequency of 1.6 Hz and strain of 0.02%, heating rate was set to 1 K min^−1^. *T*_g_ of 178 °C was determined at maximum for loss modulus G’’. d) pXRD profile reveals a crystallinity of 38% by comparing the crystalline and amorphous areas.

As shown by TGA of **PLU**, the onset decomposition temperature at 5% mass loss (*T*_d_) of 252 °C fits into the general decomposition range of urethane groups between 200 and 300 °C (Figure 6a) and compares with conventional polyurethanes.^[^ ^1^, ^63^ ^]^ Other polyurethanes produced by ROP are only stable up to around 200 °C, giving **PLU** a significantly higher *T*_d_ in comparison.^[^ ^13^, ^64^ ^]^ DSC analysis revealed a glass transition temperature (*T*_g_) of 154 °C, that is slightly higher compared to the ring‐opening polyurethane produced by Thomas et al. (150 °C) (Figure 6b).^[^ ^13^ ^]^ In general, the properties of polyurethanes produced by ROP are entirely different from those of conventional polyurethanes owing to the high density of urethane linkages. The industrial polymers produced by polyaddition of isocyanates and polyols have a significantly lower proportion of urethane linkages, and their properties are strongly influenced by the polyol soft segments.^[^ ^1^, ^65^ ^]^

The dynamic thermomechanical properties of **PLU** were studied by applying a rheology temperature sweep (Figure 6c). At approximately 170 °C, the loss modulus G’’ exceeds the storage modulus G’ and reaches its maximum at 178 °C, corresponding to *T*_g_. The values for *T*_g_ determined by DSC and rheology are of the same order of magnitude but show a difference of 24 °C. This phenomenon is frequently reported in the literature and can be explained by the temperature ramp dependence and the frequency dependence of rheology.^[^ ^66^, ^67^ ^]^ However, in both measurements no melting point was detected for **PLU**, a remarkable finding given the high density of urethane functionalities for possible hydrogen bonds. A possible reason for this could be that the melting point of **PLU** is above or in the range of the decomposition temperature. Therefore, **PLU** was analyzed by pXRD (Figure 6d). This revealed a crystalline fraction of the polymer of 38%, giving **PLU** semi‐crystalline properties. The semi‐crystallinity of **PLU** can be attributed to the high density of urethane moieties in the polyurethane backbone, its stereoregularity arising from using the stereoregular monomer **LU**, or both. Attempts to produce dog bone‐shaped specimens for tensile testing by hot press molding failed due to the high brittleness of **PLU**, as the specimens break on removal from the mold. Possibly enhanced mechanical properties can be realized by higher molar masses. In summary, the (*R*)‐limonene‐based **PLU** possesses semi‐crystalline properties with a high *T*_g_ and *T*_d_, making it an interesting material for further investigation. In upcoming studies, **PLU** shall be used as a rigid block for synthesizing polyetherurethanes or polyesterurethanes by ring‐opening copolymerization. Moreover, efforts to transfer the coordinative ROP of cyclic carbamates to 3‐carene or *α*‐pinene‐based monomers are underway to extend the scope of this synthetic strategy toward isocyanate‐ and phosgene‐free polyurethanes.

## Conclusion

In summary, this study presents a phosgene‐ and isocyanate‐free route for synthesizing terpene‐based polyurethanes using (*R*)‐limonene as a bio‐based starting material by catalytic ring‐opening polymerization. Dimethyl carbonate is used as a sustainable carbonylation reagent instead of phosgene. NMR, SC‐XRD, ESI‐MS, GC‐MS, and elemental analysis confirm the successful synthesis of limonene carbamate **LU** and its precursors. Among the initiators and catalysts investigated, Sn(Oct)_2_ proved best suited to polymerize the terpene‐based cyclic carbamate **LU** in a ring‐opening manner via a coordinative mechanism. Sn(Oct)_2_ showed the best performance and achieved up to 93% conversion at 100 °C with a molecular weight *M*_n_ of 9.9 kg mol^−1^ and a degree of polymerization of 49. Applying a higher monomer‐to‐catalyst ratio of 200:1 led to a molecular weight *M*_n_ of 16.0 kg mol^−1^ with a polydispersity *Đ* of 1.5. In addition, kinetic studies, end group analysis via ESI‐MS and stoichiometric ninhydrin experiments, *N*–*H* methylation of **LU**, kinetic isotope experiments, and ^¹¹9^Sn NMR experiments provide mechanistic insights. During the polymerization process, activator **A1** facilitates the polymer chain initiation, leading to higher conversions and fewer side reactions. Depolymerization of **PLU** in solution was achieved in high yield and purity using Sn(Oct)_2_ as catalyst at 140 °C. The thermal properties of **PLU** were investigated using TGA, DSC, rheology, and pXRD, which revealed a decomposition temperature of 252 °C, a glass transition temperature above 150 °C, and a crystallinity of 38%, giving **PLU** semi‐crystalline properties. Overall, this study represents a phosgene‐ and isocyanate‐free approach to producing polyurethanes from renewable resources and lays the groundwork for future developments in bio‐based polyurethanes via ring‐opening polymerization.

## Supporting Information

The Supporting Information (SI) is available free of charge from the Wiley Angewandte Chemie website at DOI: 10.1002/anie.20252727.

Synthetic procedures, polymerization procedures, and ninhydrin experiments. Detailed characterization data (^1^H and ^13^C NMR, DEPT 135 and 2D analysis, elemental analysis, ESI‐MS, GC‐MS, and and TLC).

The authors have cited additional references within the Supporting Information.^[^ ^68^, ^69^, ^70^, ^71^, ^72^, ^73^ ^]^

## Author Contributions

The manuscript was written through contributions from all authors. All authors have given approval to the final version of the manuscript. J.F.: conceptualization, data curation, formal analysis, visualization, writing—review and editing; L.F.R.: crystallography; S.H.: review and editing; M.K.: review and editing; B.R.: funding acquisition, supervision, project administration, resources, writing—review and editing.

## Conflict of Interests

The authors declare no conflict of interest.

## Supporting information



Supporting Information

Supporting Information

## Data Availability

The data that support the findings of this study are available in the Supporting Information of this article.
