# Supplementary material for: Coordinative Ring‐Opening Polymerization of Limonene Carbamate Toward Phosgene‐ and Isocyanate‐Free Polyurethane
Source: Angew Chem Int Ed Engl. 2025 Jul 23;64(33):e202502727. doi: 10.1002/anie.202502727 (PMC12338414; doi:10.1002/anie.202502727)
Supplement: Supplementary file 1 — Supporting Information [file ANIE-64-e202502727-s001.docx]

*Supporting Information for:*

**Coordinative Ring-Opening Polymerization of Limonene Carbamate towards Phosgene- and Isocyanate-Free Polyurethane – Supporting Information**

Jonas Futter^a^, Leon F. Richter^b^, Stefanie Hörl^a^, Moritz Kränzlein^a^, and Bernhard Rieger^a,*^

*^a^* WACKER-Chair of Macromolecular Chemistry, Catalysis Research Center, Department of Chemistry, Technical University of Munich, Lichtenbergstr. 4, 85748 Garching (Germany)

*^b^* Molecular Catalysis, Catalysis Research Center, Department of Chemistry, Technical University of Munich, Lichtenbergstr. 4, 85748 Garching (Germany)

Corresponding Author

*E-mail [rieger@tum.de](mailto:rieger@tum.de) (B.R.).

****Table of Contents****

1. General experimental 2
2. Synthesis procedures 6
3. Polymerization procedure 35
4. Ninhydrin end group analysis 44
5. Methylated LU 45
6. Chemical recyclability and hydrolytic degradation 47
7. References 50

1. General Experimental

All reactions and polymerizations with moisture and air-sensitive reactants were carried out in an M*Braun* LabMaster120 glovebox filled with argon 4.6 from *Westfalen* or using standard *Schlenk* techniques. All glassware was heat-dried before use. All chemicals were purchased from *Sigma-Aldrich*, ABCR, or TCI Europe and used without further purification unless otherwise stated. The catalyst Sn(Oct)_2_ was distilled prior to use. Dichloromethane, tetrahydrofuran, toluene, and *n*-pentane were dried using an M*Braun* SPS-800 solvent purification system and stored over a 3 Å molecular sieve. Dioxane was distilled and stored over a 3 Å molecular sieve.

**Thin layer chromatography (TLC)** was performed with silica pre-coated polyester sheets (*Macherey-Nagel*, 0.20 mm silica 60, F254). The detection of the molecules took place with UV light (*λ* = 254, 365 nm) or aqueous potassium permanganate solution. Column chromatography was conducted with *Merck* silica (230-400 mesh) and the described eluent.

**Nuclear magnetic resonance (NMR)** spectra were recorded on a *Bruker* AV-400HD at 400 MHz (^1^H), 100 MHz (^13^C), and 150 MHz(^119^Sn) at ambient temperature. The chemical shifts are stated in *δ*-units relative to the residual proton or carbon signal of the deuterated solvent [CHCl_3_: *δ* (^1^H) = 7.26 ppm, *δ* (^13^C) = 77.16 ppm; CH_2_Cl_2_: *δ* (^1^H) = 5.32 ppm, *δ* (^13^C) = 53.84 ppm; C_6_H_6_: *δ* (^1^H) = 7.16 ppm, *δ* (^13^C) = 128.06 ppm; toluene-*d*_8_: *δ* (^1^H) = 2.08, 6.97, 7.01 and 7.09 ppm, *δ* (^13^C) = 137.86, 129.26, 128.33, 125.49 and 20.40 ppm]. ^119^Sn NMR chemical shift are stated relative to tetramethyltin. Deuterated solvents were purchased from Sigma-Aldrich or Deutero and dried over 3 Å molecular sieves before use. Spectra interpretation was performed using MestreNova software. The indication of the atoms was done italicizing the respective fragment of the molecule and not according to the IUPAC numbering. Spectral data is provided as follows: chemical shift (multiplicity, integration, coupling constant, assignment). The multiplicity of the signals was assigned as follows: bs - broad singlet, s - singlet, d - doublet, t - triplet, q - quartet, m - multiplet or combination thereof. Coupling constants are stated in Hz and given as averaged values from the experimental data. The assignment of the signals was realized *via* two-dimensional NMR experiments: ^1^H^1^H COSY, ^1^H^13^C HSQC, and DEPT 135 or ^1^H^15^N HMBC.

**Gas-chromatography mass spectrometry (GC-MS)** measurements were performed on an GC-7890B from *Agilent Technologies* equipped with a MSD 59771 mass detector, a 7693 automatic liquid sampler and a G4513A auto injector. Sample separation is done using a HP-5MS UI column (30.0 m length, 0.25 mm diameter, 0.25 μm film) in a temperature range of 60-300 °C followed by mass spectrometry using full scan method in a mass range of 40-500 au. Samples are prepared by dissolving 1 mg/mL in HPLC grade acetonitrile prior to measurement.

**Electron-spray Ionization mass spectrometry (ESI-MS)** was measured on a *Thermo Fisher Scientific* Exactive Plus in positive mode in HPLC acetonitrile. Oligomers were prepared using a 1:25 ratio of catalyst to monomer with the conditions reported in the manuscript and were directly measured from the reaction mixture after stirring for 3 hours without purification.

**Matrix-assisted laser desorption ionization mass spectrometry (MALDI-MS)** was performed on a *Bruker* Daltonics mircoflex. Polymer samples of **PLU** with a concentration of 2 mg/mL were prepared and 0.5 µL of this solution was mixed with 0.5 µL of matrix solution (saturated dithranol in water (+ 0.1 vol% TFA):MeCN = 2:1). Spectra interpretation was performed using *Bruker* FlexAnalysis.

**Elemental analyses (EA)** were carried out by the micro analytical laboratory of the faculty of chemistry at the Technical University of Munich (TUM) using a Vario EL from *Elementar*.

**Gel permeation chromatography (GPC)** was used to determined average molecular weights *M_n_*, *M_w_* and polydispersities (*Đ*) of the polymers with a sample concentration of 2 mg/mL. Measurements were performed on an *Agilent* PL-GPC 50 with an integrated RI unit, two light scattering detectors (15° and 90°) and a differential pressure viscosimeter with two *Agilent* PolarGel M columns. As eluent *N*,*N*-dimethylformamide (with 2.096 g/L lithium bromide added) at 30 °C was used with a flow rate of 1.0 mL/min and the resulting spectra were referenced to poly(methylmethacrylate) calibration standards.

**Fourier-transform infrared spectroscopy (FT-IR)** measurements were recorded on a nitrogen-cooled *Bruker* Vertex 70A spectroscope on an attenuated total reflection module.

**Melting point** and **decomposition temperature** of **LU** were determined in glass capillaries with a *Büchi* Melting Point B-540.

**Ultraviolet–visible (UV-Vis) spectroscopy** measurements were carried out on a *Thermo Fisher* Multiskan FC using a 10 × 10 mm diameter UV quartz cuvette with a screw cap PTFE sealing from *Hellma Analytics*. The temperature (25 (±0.5ºC) was set 10 min before starting the measurement. Ethanol was used as solvent for all measurements.

**Thermogravimetric analysis (TGA)** was performed from 1-2 mg samples on a TGA Q5000 by *TA Instruments*. Samples were heated from room temperature to 700 °C with a heat rate of 10 K/min under argon. Analysis of mass loss and determination of *T*_d,5%_ is done using *TA Analysis* software.

**Differential scanning calorimetry (DSC)** was measured using a DSC Q2000 by *TA Instruments* in exo-down mode. Sample size is about 6-10 mg in non-hermetic aluminum pans in the range of –150 °C to 200 °C. Analysis is performed using *TA Analysis*.

**Rheology** measurements were performed on an MCR 302 rheometer from *Anton Paar* with an upper plate (25.0 mm diameter) as a counterpart and a gap size of 0.50 mm. Polymer samples were heated to the desired temperature through an upper and lower Peltier system using an additional protective hood. Strain-sweeps were performed at 25, 140, and 180 °C at a fixed frequency of 1.6 Hz. Thermomechanical measurements were performed in duplicates at a fixed frequency of 1.6 Hz, and strain of 0.02%, and the heating rate was set to 1 K/min (ASTM-D4065-20 standard). Data was monitored *via* Rheoplus software.

**Powder X-Ray diffraction (pXRD)** was performed using a PANalytical Empyrean diffractometer from *Malvern Panalytical* with a PANalytical PIXcel 1D detector in Bragg-Brentano geometry. Cu K_α_ radiation with a voltage of 45 kV (40 mA intensity) with *λ*1 = 1.5406 Å / *λ*2 = 1.5444 Å [I1/I2 = 0.5] was used for measurements in the 2*θ* of 5-60 ° (step size 0.01 °, measurement time 7 h). The obtained data was analyzed using HighScore software, Cu K_α_ is stripped using the *Rachinger* method and the amorphous background is determined using the *Sonneveld* and *Visser* method with a granularity of 15 and a bending factor of 2 for the subsequent peak fit. The calculation of crystallinity fraction in the region of 6 – 36 ° is done by a peak deconvolution using OriginPro 2020 with one peak for the amorphous backscattering of the polymer and six peaks for the crystalline diffraction peaks. The degree of crystallinity X_C_ is obtained by peak integration and the areas of the amorphous parts A_A_ and crystalline parts A_C_ *via* X_C_ = A_C_ / (A_C_ + A_A_).^[1]^

**Single-crystal X-Ray diffraction (SC-XRD)** data were collected on a *Bruker* D8 Venture single crystal-X-ray diffractometer equipped with a CMOS detector (*Bruker* Photon-100), a TXS rotating anode with Mo K_α_ radiation (*λ* = 0.71073 Å) and a Helios optic using the APEX4 software package. Measurements were performed on single crystals coated with perfluorinated ether. The crystals were fixed on top of a Kapton micro sampler and frozen under a stream of cold nitrogen. A matrix scan was used to determine the initial lattice parameters. Reflections were corrected for Lorentz and polarisation effects, scan speed, and background using SAINT. Absorption corrections including odd and even ordered spherical harmonics were performed using SADABS. Space group assignments were based upon systematic absences, E statistics, and successful refinement of the structures. The structures were solved using SHELXT with the aid of successive difference Fourier maps, and were refined against all data using SHELXL in conjunction with SHELXLE.^[2,3]^ Hydrogen atoms were placed in calculated positions and refined using a riding model, with methylene, aromatic, and other C–H distances of 0.99 Å, 0.95 Å, and 1.00 Å, respectively, and U_iso_(H) = 1.2U_eq(C)_. Non-hydrogen atoms were refined with anisotropic displacement parameters. Full-matrix least-squares refinements were performed by minimizing Σ*w*(*F_c_*^2^ – *F_0_*^2^)^2^ with the SHELXL weighting scheme.^[2]^ Neutral atom scattering factors for all atoms and anomalous dispersion corrections for the non-hydrogen atoms were taken from International Tables for Crystallography.^[4]^ Images of the crystal structures were generated with Platon.^[5]^ CCDC 2392925 contains the supplementary crystallographic data for this paper. These data can be obtained free of charge *via* www.ccdc.cam.ac.uk/data_ request/cif or by emailing data_request@ccdc.cam.ac.uk or by contacting The Cambridge Crystallographic Data Centre, 12 Union Road, Cambridge CB2 1EZ, UK; fax: +44 1223 336033.

| CCDC | 2392925 |
| --- | --- |
| formula | C_11_H_17_NO_2_ |
| formula weight [g mol^-1^] | 195.25 |
| space group | P 41 21 2 |
| *a, b, c* [Å] | 7.5848(3), 7.5848(3), 37.073(3) |
| α, β, γ [°] | 90, 90, 90 |
| *V* [Å^3^] | 2132.8(2) |
| *Z* | 8 |
| *F*(000) | 848 |
| *T* [K] | 100 |
| *D_calc_* [g cm^-3^] | 1.216 |
| *µ* [mm^-1^] | 0.083 |
| R_1_ (*I*>2*σ*(*I*)) | 3.3 |
| *w*R_2_ (all data) | 7.47 |
| Δρ_min_, Δρ_max_ [e Å^-3^]^*^ | -0.139, 0.137 |

**2. SYNTHESIS PROCEDURES**

*cis*-Limonene epoxide **1** was prepared according to an adapted literature procedure.^[6]^

***(1R,4R,6S)-1-methyl-4-(prop-1-en-2-yl)-7-oxabicyclo[4.1.0]heptane*** (**1**):

 (*R*)-Limonene (22.0 g, 162 mmol, 1.00 eq.) was dissolved in DCM (450 mL, 0.36 M), and (*R*,*R*)-Mn(III) *Jacobsen* catalyst (5.08 g, 7.87 mmol, 0.05 eq.) and NMO (56.8 g, 485 mmol, 3.00 eq.) were added. m-CBPA (50.2 g, 291 mmol, 1.80 eq.) dissolved in DCM (450 mL) was added dropwise at –40 °C over 2 hours. The mixture was stirred at –40 °C for 4 hours, and reaction progress was monitored by GC-MS. A saturated solution of sodium bicarbonate (400 mL) was added until no gas formation could be observed. The organic phase was separated from the aqueous phase and washed with water (3 × 300 mL). The organic phase was dried over sodium sulfate, filtered, and the solvent was carefully removed by rotary evaporation without vacuum at 50 °C. The crude product was purified via column chromatography using *n*-pentane/diethyl ether (40:1) as eluent, yielding cis-(R)-limonene oxide **1** (8.19 g, 53.8 mmol, 68%) as a yellow liquid.

TLC: R_f_ = 0.23 (silica, *n*-pentane/diethyl ether 50:1) [KMnO_4_]

^1^H NMR (400 MHz, CDCl_3_) *δ* (ppm) = 4.72 (d, 1H, ^2^*J*_HH_ = 1.5 Hz, H‑10), 4.67 (d, 1H, ^2^*J*_HH_ = 1.5 Hz, H‑10), 3.06 (s, 1H, H‑1), 2.17‑2.08 (m, 2H, H‑5, H‑6), 1.87‑1.83 (m, 2H, H‑3), 1.69 (s, 3H, H‑9), 1.68‑1.64 (m, 1H, H‑6), 1.57‑1.50 (m, 1H, H‑4), 1.31 (s, 3H, H‑7), 1.28‑1.15 (m, 1H, H‑4).

^13^C{^1^H} NMR (100 MHz, CDCl_3_) *δ* (ppm) = 149.19 (C‑8), 109.17 (C‑10), 60.69 (C‑1), 57.50 (C‑2), 36.35 (C‑5), 30.87 (C‑6), 28.77 (C‑3), 26.07 (C‑4), 24.44 (C‑7), 21.24 (C‑9).

Elemental analysis: calc. for C_10_H_16_O: C, 78.90; H, 10.59; O, 10.51; found: C, 78.54; H, 10.40.

ESI-MS: m/z = calc. for [C_10_H_16_O]^+^: 152.1201 ([M]^+^); found 152.1203.

GC-MS: t_R_ = 7.362 min, m/z = 152.1 ([M]^+^), 137.1 ([M-CH_3_]^+^).

**
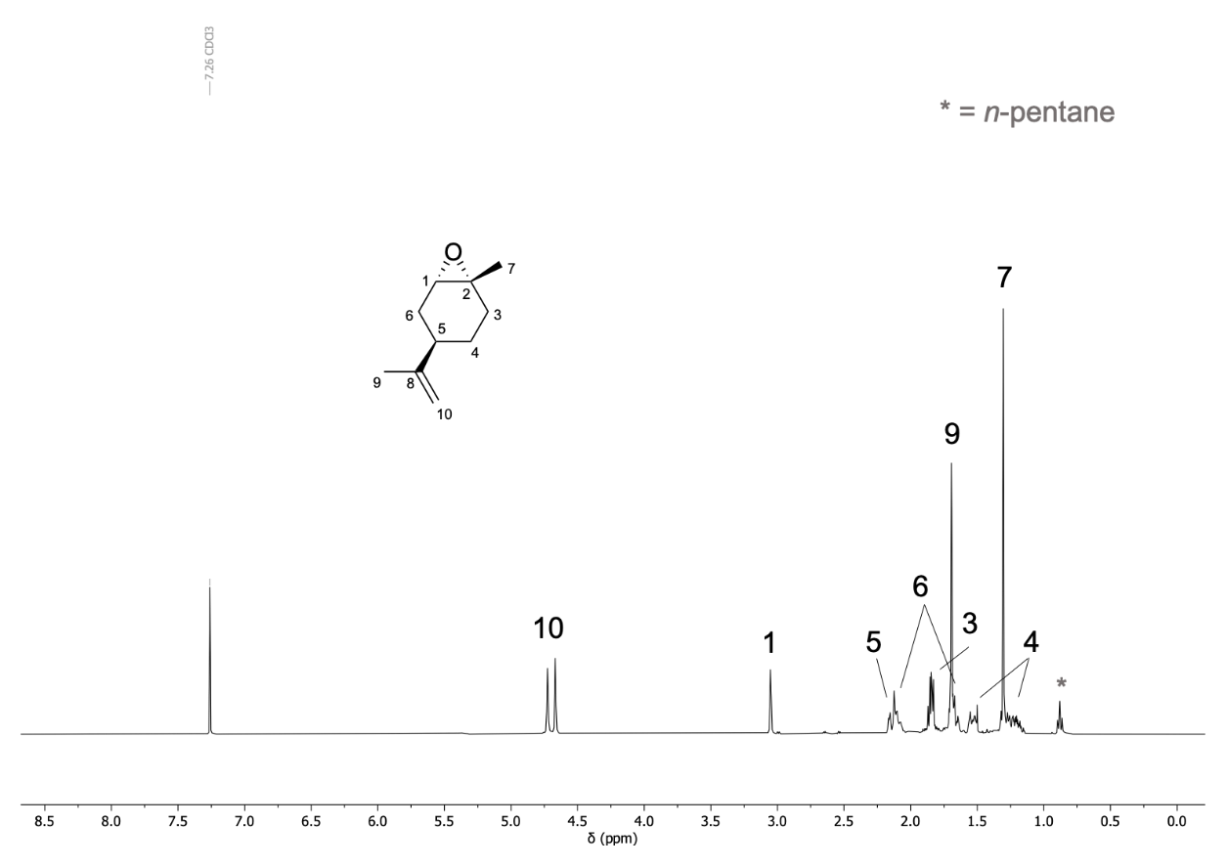
**

**Figure S1.** ^1^H NMR spectrum of *cis*-limonene epoxide **1** in CDCl_3_.

**
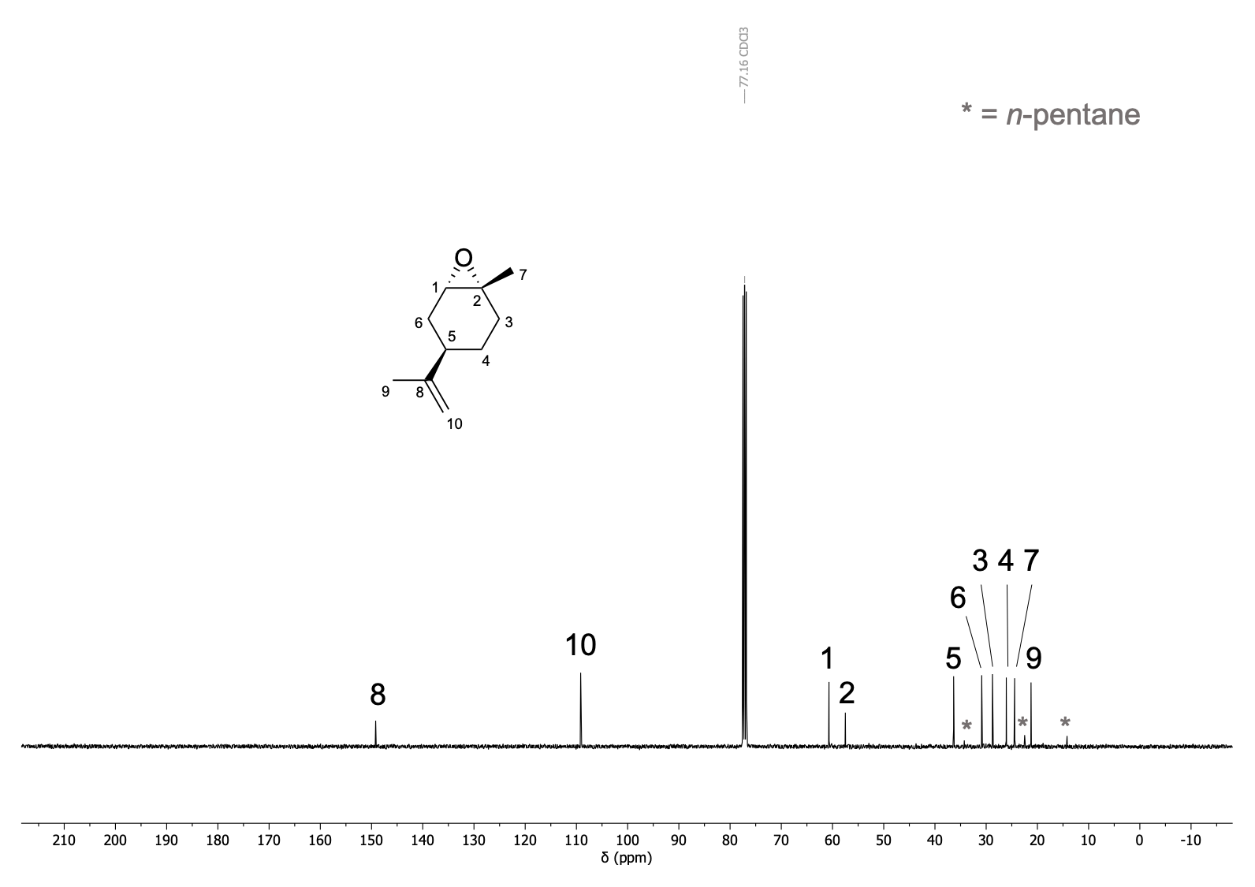
**

**Figure S2.** ^13^C{^1^H} NMR spectrum of *cis*-limonene epoxide **1** in CDCl_3_.


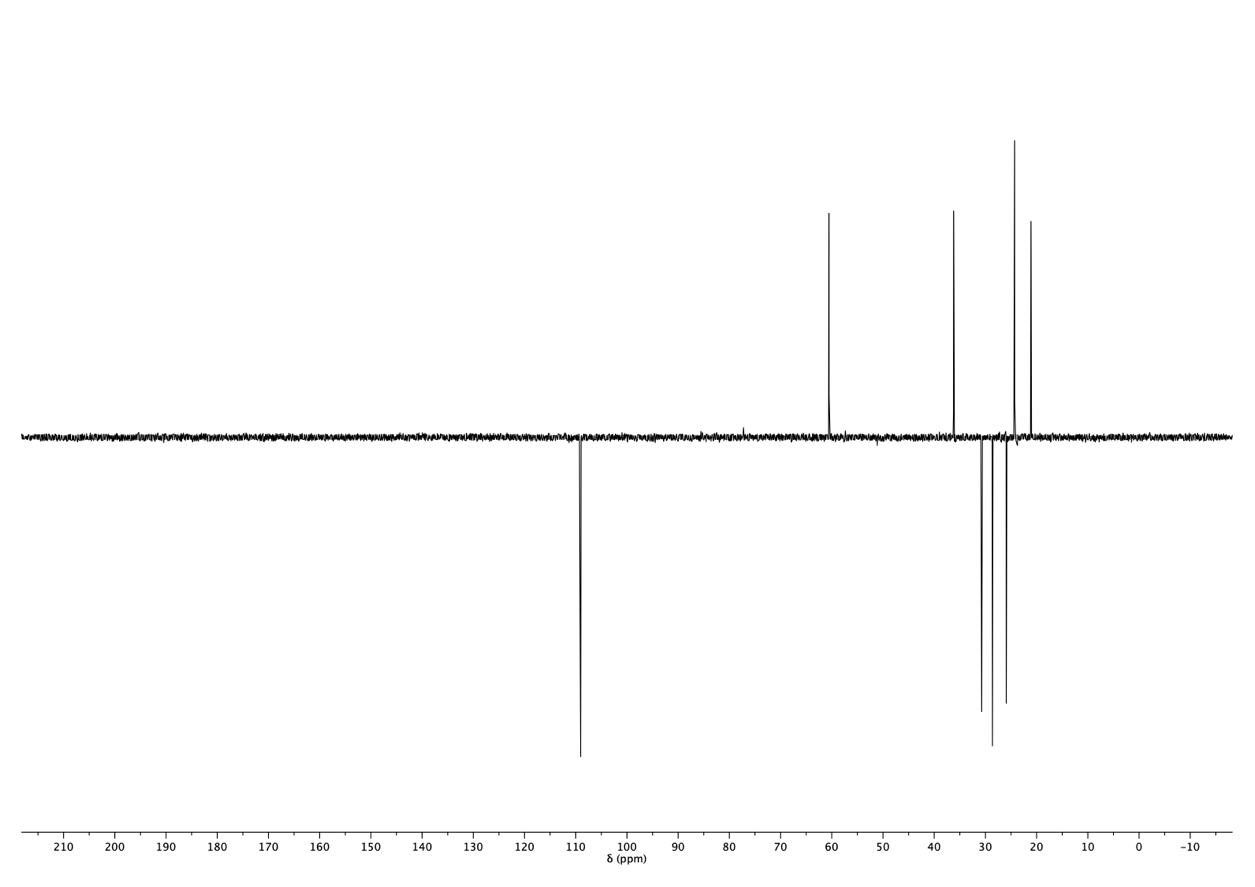


**Figure S3.** DEPT 135 spectrum of *cis*-limonene epoxide **1** in CDCl_3_.

**
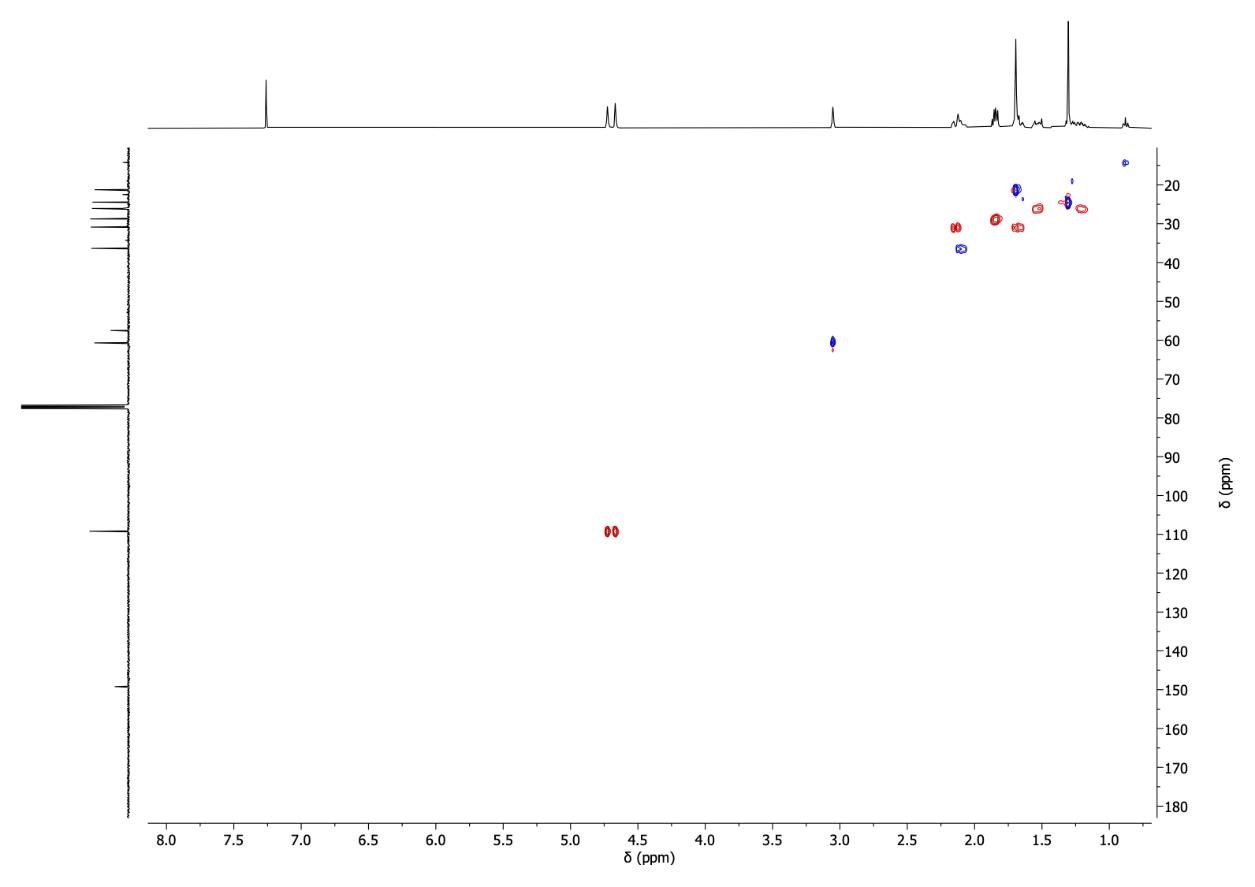
**

**Figure S4.** ^1^H^13^C HSQC spectrum of *cis*-limonene epoxide **1** in CDCl_3_.

**
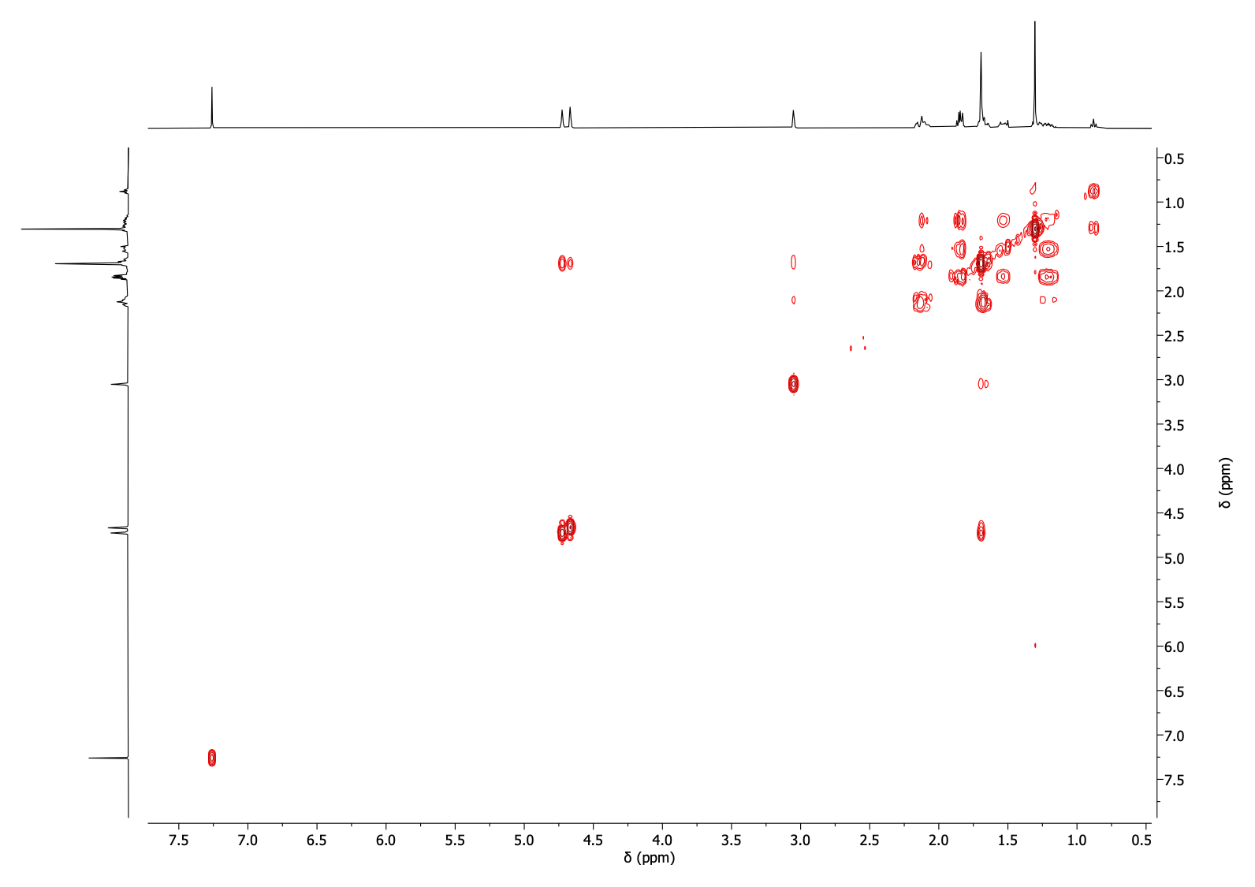
**

**Figure S5.** ^1^H^1^H COSY spectrum of *cis*-limonene epoxide **1** in CDCl_3_.

***(1S,2S,5R)-2-amino-2-methyl-5-(prop-1-en-2-yl)cyclohexan-1-ol*** (**2**):

**cis-(R)-limonene oxide **1** (2.5 g, 16.4 mmol, 1.00 eq.) was placed in a pressure tube and aqueous ammonia (6.20 mL, 25% NH_3_ in water, 82.1 mmol, 5.00 eq.) was added. The pressure tube was sealed and the reaction was stirred at 100 °C for 3 days. After cooling to room temperature, water removed under reduced pressure, resulting in a yellow solid. The crude product was, was dissolved in DCM (50 mL), dried over anhydrous sodium sulfate, filtered, concentrated under reduced pressure, and purified *via* sublimation (80 °C at 10^–5^ mbar), yielding amino alcohol **2** (2.62 g, 15.5 mmol, 94%) as colorless crystals.

TLC: R_f_ = 0.10 (silica, DCM/methanol 9:1) [KMnO_4_]

^1^H NMR (400 MHz, CDCl_3_) *δ* (ppm) = 4.74 (s, 2H, H‑10), 3.55 (t, 1H, ^3^*J*_HH_ = 3.8 Hz, H‑1), 2.28 (td, 1H, ^3^*J*_HH_ = 10.3 Hz, 5.1 Hz, H‑5), 1.92‑1.81 (m, 4H, H‑6, N‑H_2_, O‑H), 1.73 (s, 3H, H‑9), 1.73‑1.63 (m, 2H, H‑3, H‑6), 1.66‑1.56 (m, 1H, H‑4), 1.55‑1.48 (m, 1H, H‑4), 1.44‑1.37 (m, 1H, H‑3), 1.15 (s, 3H, H‑7).

^13^C{^1^H} NMR (100 MHz, CDCl_3_) *δ* (ppm) = 149.14 (C‑8), 109.26 (C‑10), 74.84 (C‑1), 52.01 (C‑2), 37.81 (C‑5), 34.62 (C‑3), 33.75 (C‑6), 26.30 (C‑7), 26.28 (C‑4), 21.49 (C‑9).

Elemental analysis: calc. for C_10_H_19_NO: C, 70.96; H, 11.31; N, 8.28; O, 9.45; found: C, 70.35; H, 11.46; N, 8.05.

ESI-MS: m/z = calc. for [C_10_H_19_NO]^+^: 169.1466 ([M]^+^); found 170.1544 ([M+H]^+^).

GC-MS: t_R_ = 9.350 min, m/z = 169.1 ([M]^+^), 154.1 ([M-CH_3_]^+^).

**
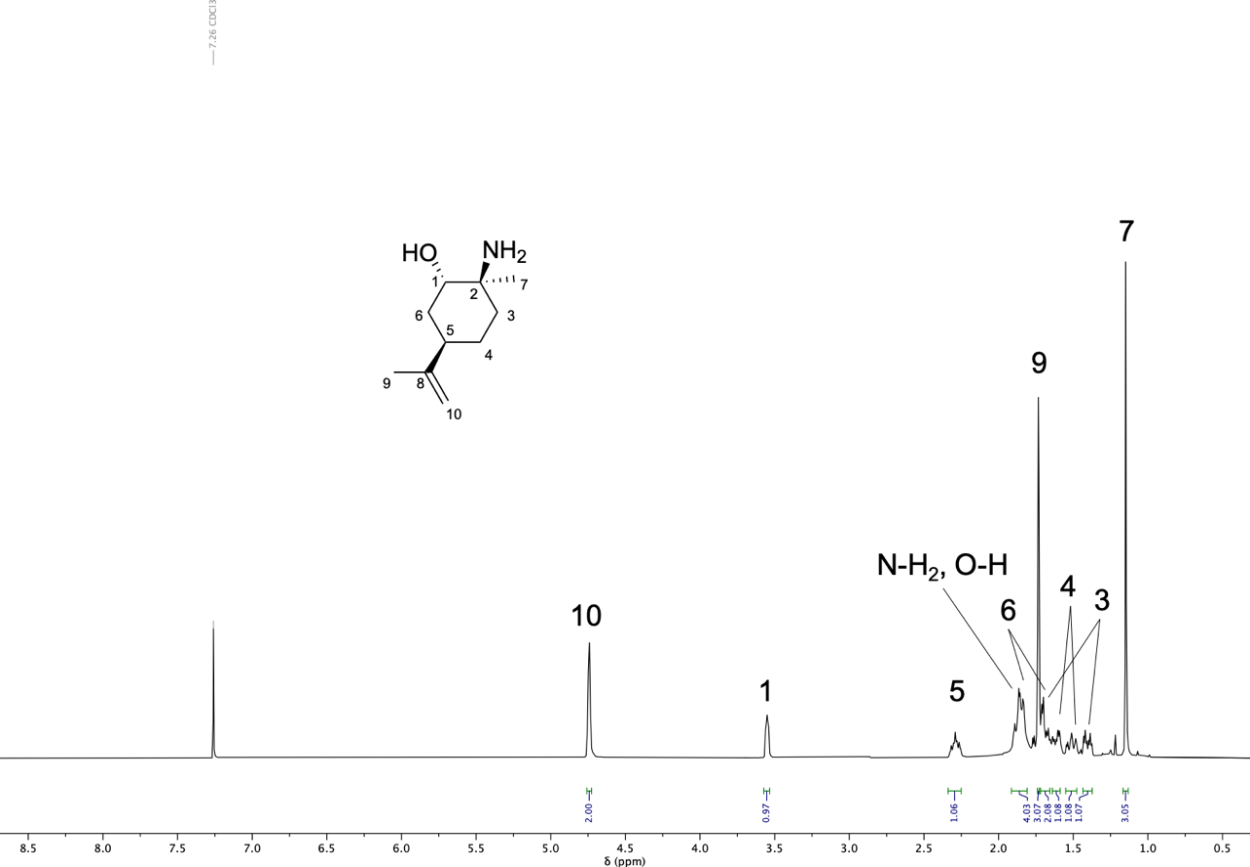
**

**Figure S6.** ^1^H NMR spectrum of amino alcohol **2** in CDCl_3_.

**
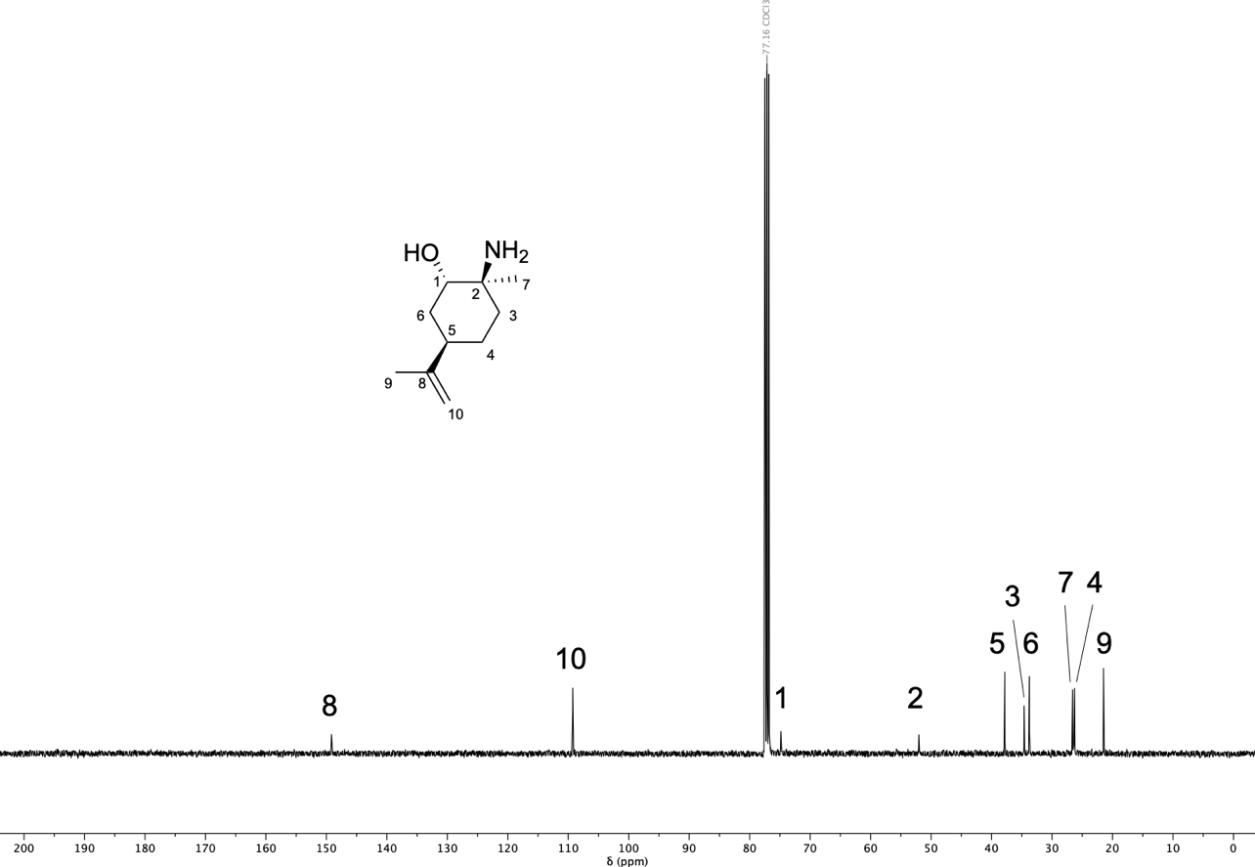
**

**Figure S7.** ^13^C{^1^H} NMR spectrum of amino alcohol **2** in CDCl_3_.


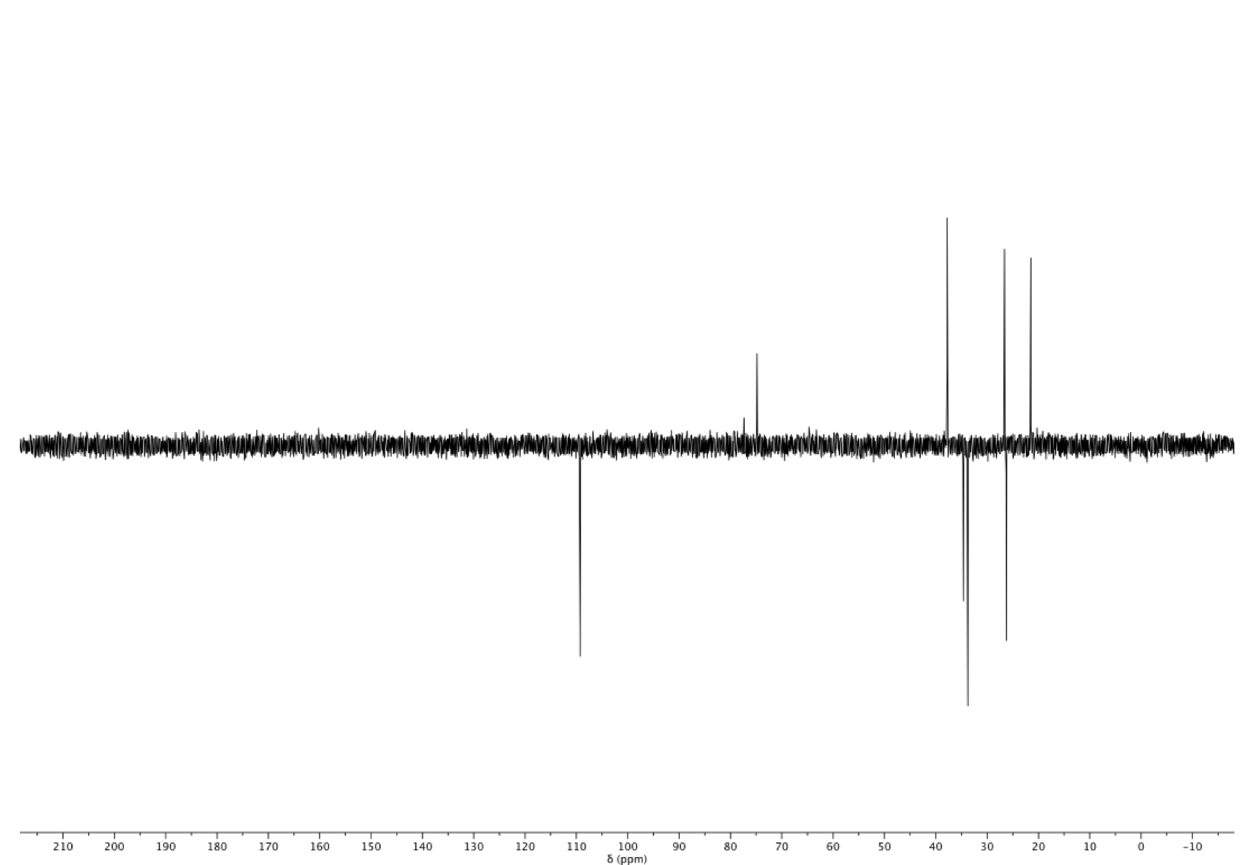


**Figure S8.** DEPT 135 spectrum of amino alcohol **2** in CDCl_3_.


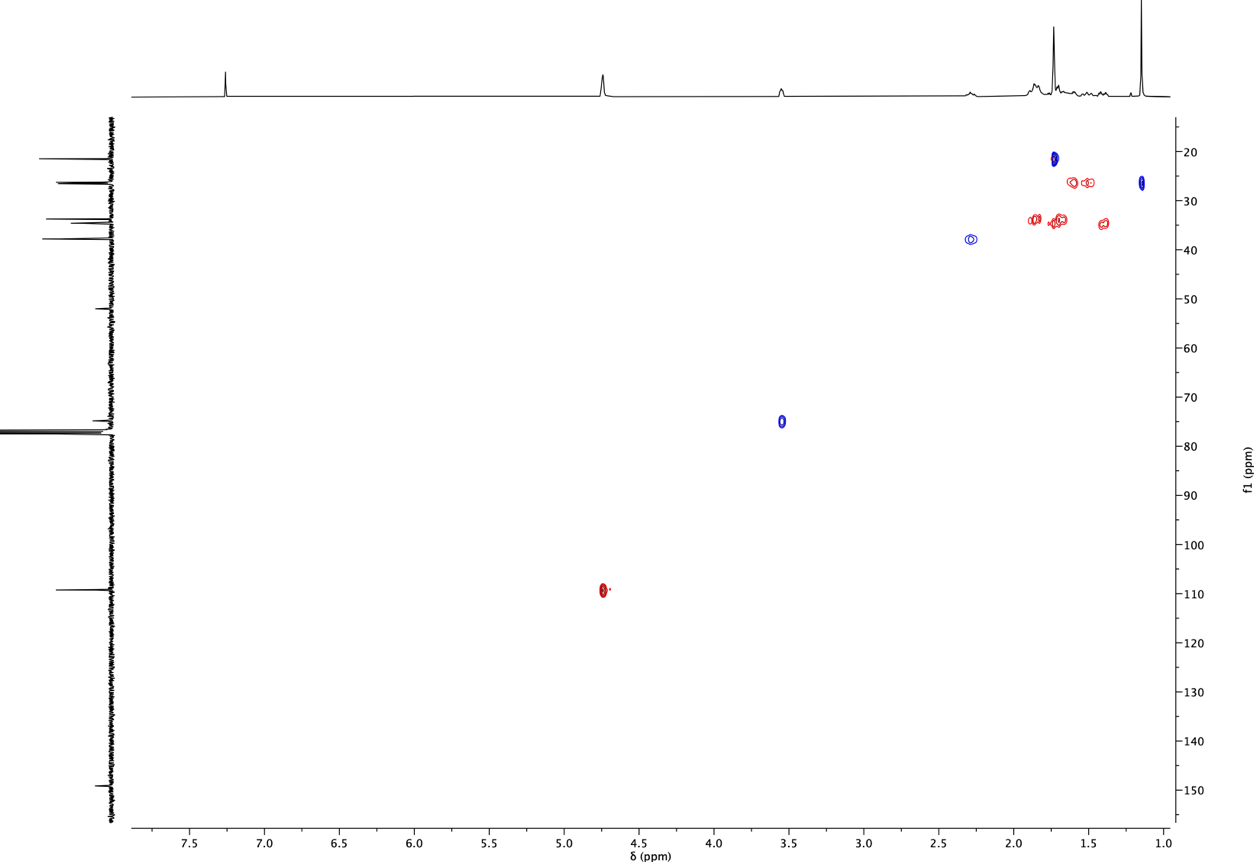


**Figure S9.** ^1^H^13^C HSQC spectrum of amino alcohol **2** in CDCl_3_.


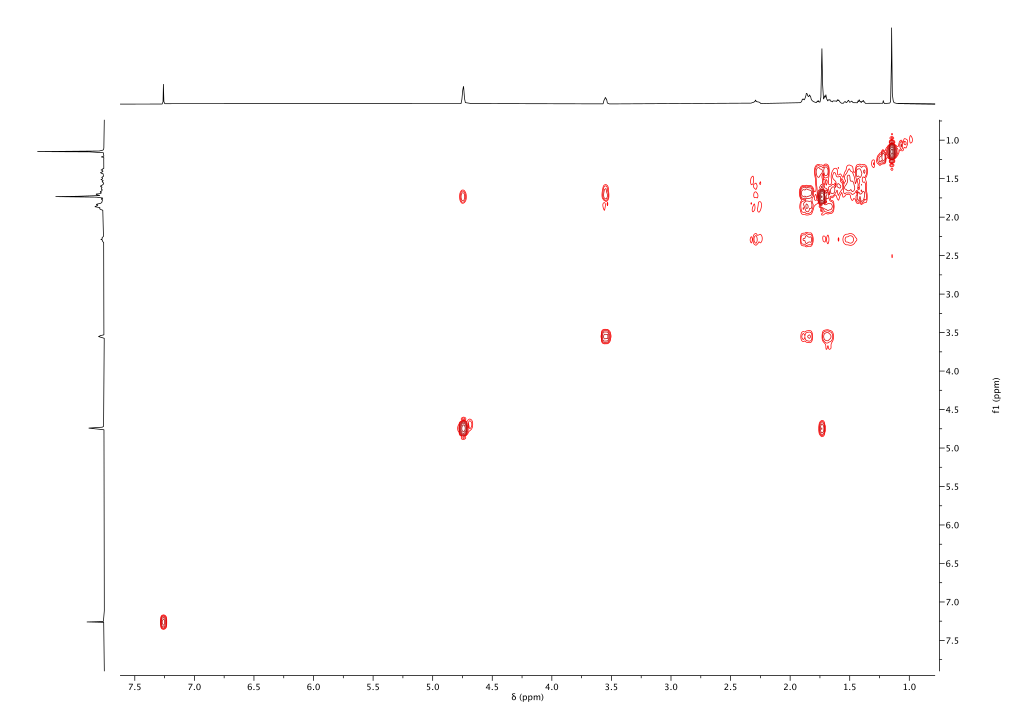


**Figure S10.** ^1^H^1^H COSY spectrum of amino alcohol **2** in CDCl_3_.


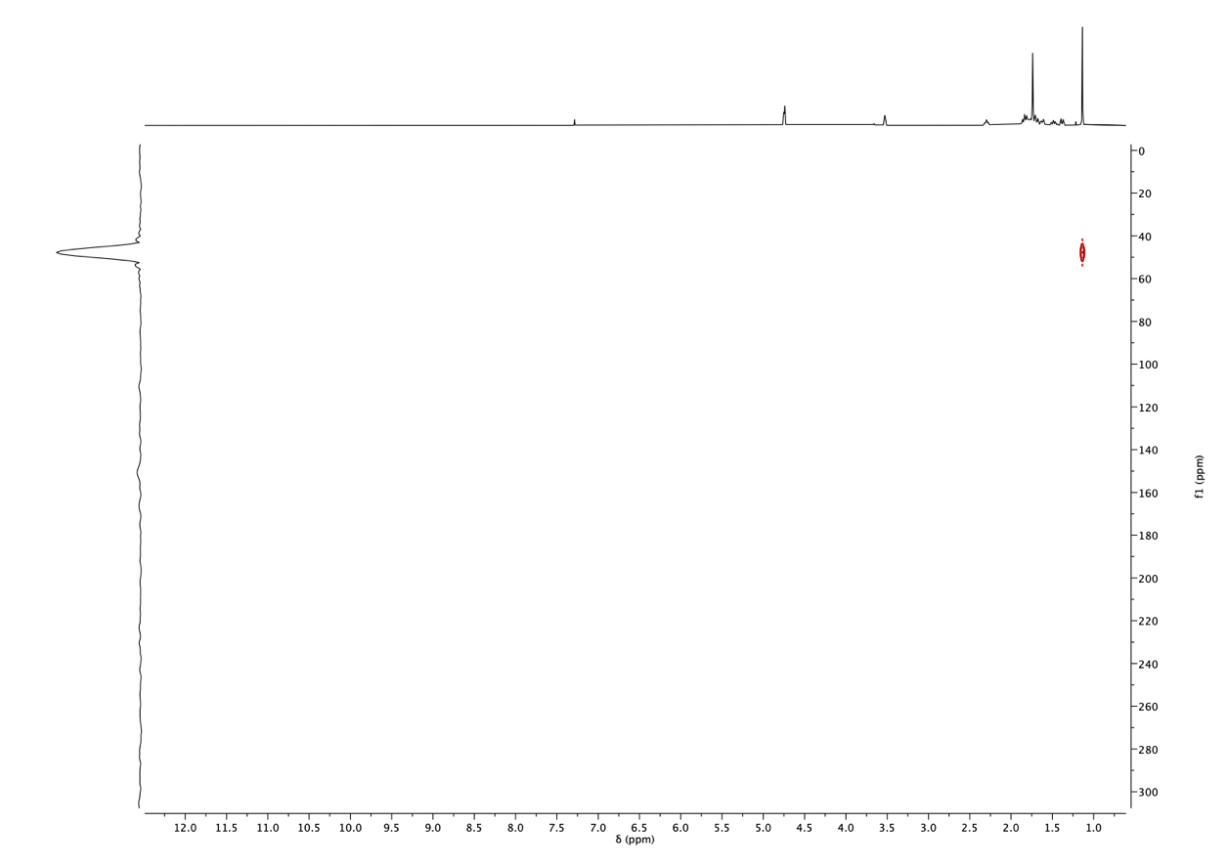


**Figure S11.** ^1^H^15^N HMBC spectrum of amino alcohol **2** in CDCl_3_.

***Methyl ((1S,4R)-2-hydroxy-1-methyl-4-(prop-1-en-2-yl)cyclohexyl)carbamate*** (**3**):

Amino alcohol **2** (5.00 g, 29.5 mmol, 1.00 eq.) was dissolved in toluene (100 mL, 0.30 M) and potassium *tert*-butoxide (3.48 g, 31.0 mmol, 1.05 eq.) was slowly added. After stirring for 30 minutes dimethyl carbonate (2.66 g, 29.5 mmol, 1.00 eq.) was added and the mixture was heated to 100 °C for 20 hours. Toluene was removed under reduced pressure and water (30 mL) was added. The aqueous solution was extracted with DCM (3 × 30 mL). The combined organic layers were dried over anhydrous sodium sulfate, filtered and evaporated under vacuum. The crude product was purified *via* flash column chromatography on silica gel using diethyl ether/*n*-pentane (3:2) as eluent to give ethyl ((1*S*,4*R*)-2-hydroxy-1-methyl-4-(prop-1-en-2-yl)cyclohexyl) carbamate (**3**) (6.36 g, 28.0 mmol, 95%)

TLC: R_f_ = 0.40 (silica, diethyl ether/*n*-pentane 3:2) [KMnO_4_]

^1^H NMR (400 MHz, CDCl_3_) *δ* (ppm) = 4.74 (d, ^2^*J*_HH_ = 11.5 Hz, 2H, H‑11), 4.59 (s, 1H, N‑H), 4.21 (s, 1H, H‑1), 3.62 (s, 3H, H‑12), 2.48 (s, 1H, O‑H), 2.39‑2.27 (m, 1H, H‑6), 1.83‑1.77 (m, 1H, H‑7), 1.76‑1.74 (m, 1H, H‑4), 1.72 (s, 3H, H‑10), 1.71‑1.68 (m, 1H, H‑7), 1.67‑1.64 (m, 1H, H‑4), 1.62‑1.58 (m, 1H, H‑5), 1.48‑1.42 (m, 1H, H‑5), 1.39 (s, 3H, H‑8).

^13^C{^1^H} NMR (100 MHz, CDCl_3_) *δ* (ppm) = 155.93 (C‑2), 148.80 (C‑9), 109.40 (C‑11), 70.49 (C‑1), 55.46 (C‑3), 51.92 (C‑12), 37.23 (C‑6), 33.62 (C‑7), 32.66 (C‑4), 25.72 (C‑5), 21.98 (C‑8), 21.51 (C‑10).

Elemental analysis: calc. for C_12_H_21_NO_3_: C, 63.41; H, 9.31; N, 6.16; O, 21.12; found: C, 63.46; H, 9.32; N, 6.21.

ESI-MS: m/z = calc. for [C_12_H_21_NO_3_]^+^: 227.1521 ([M]^+^); found 227.1525.

GC-MS: t_R_ = 12.408 min, m/z = 227.1 ([M]^+^), 209.1 ([M-H_2_O]^+^).

**
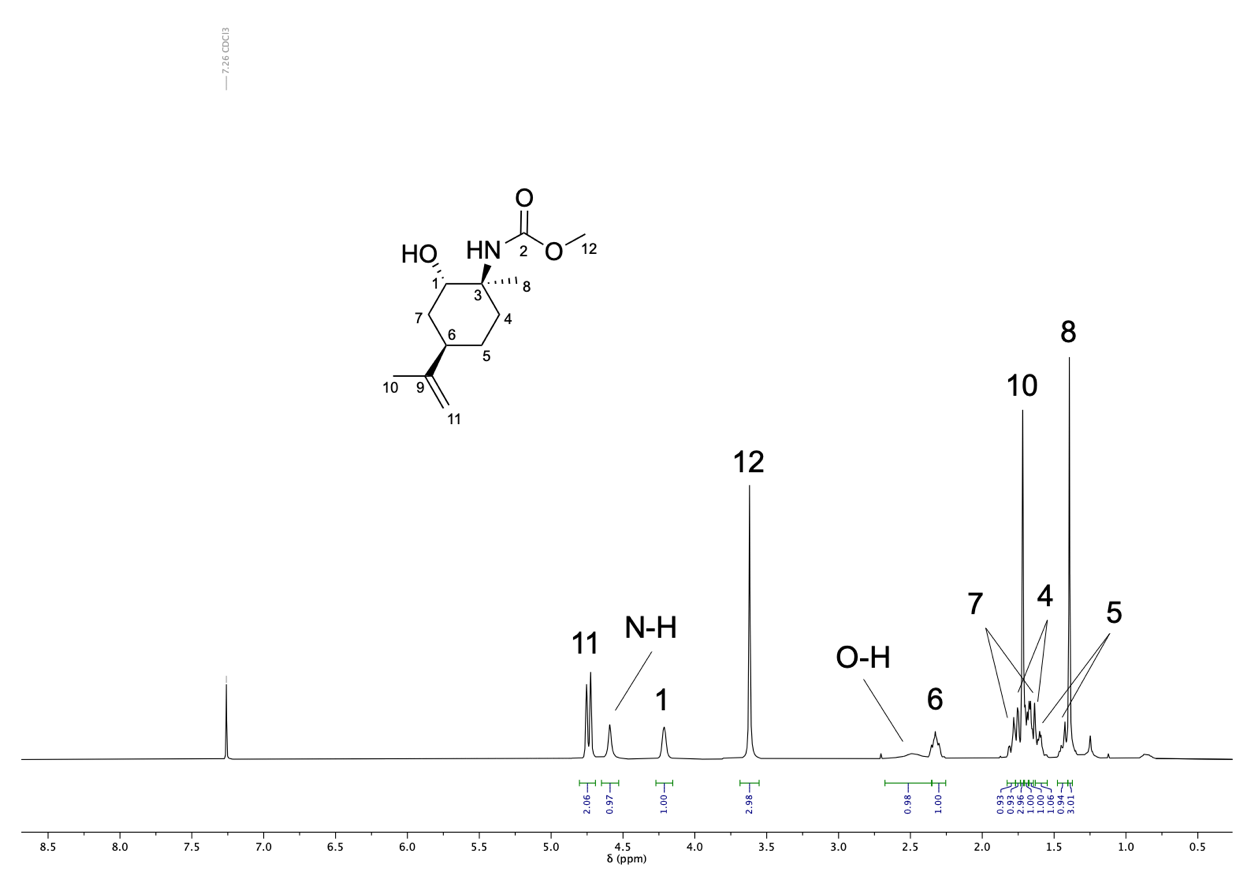
**

**Figure S12.** ^1^H NMR spectrum of carbamate **3** in CDCl_3_.


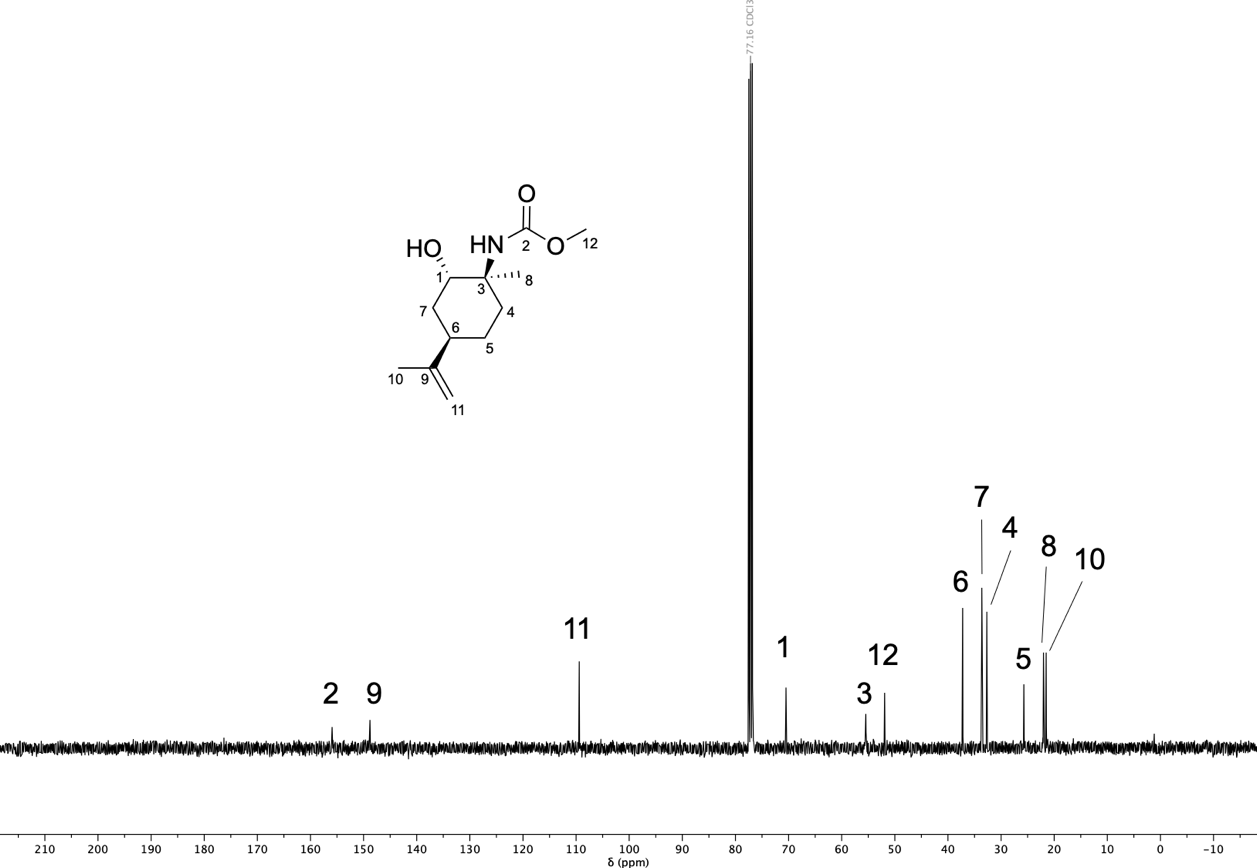


**Figure S13.** ^13^C{^1^H} NMR spectrum of carbamate **3** in CDCl_3_.


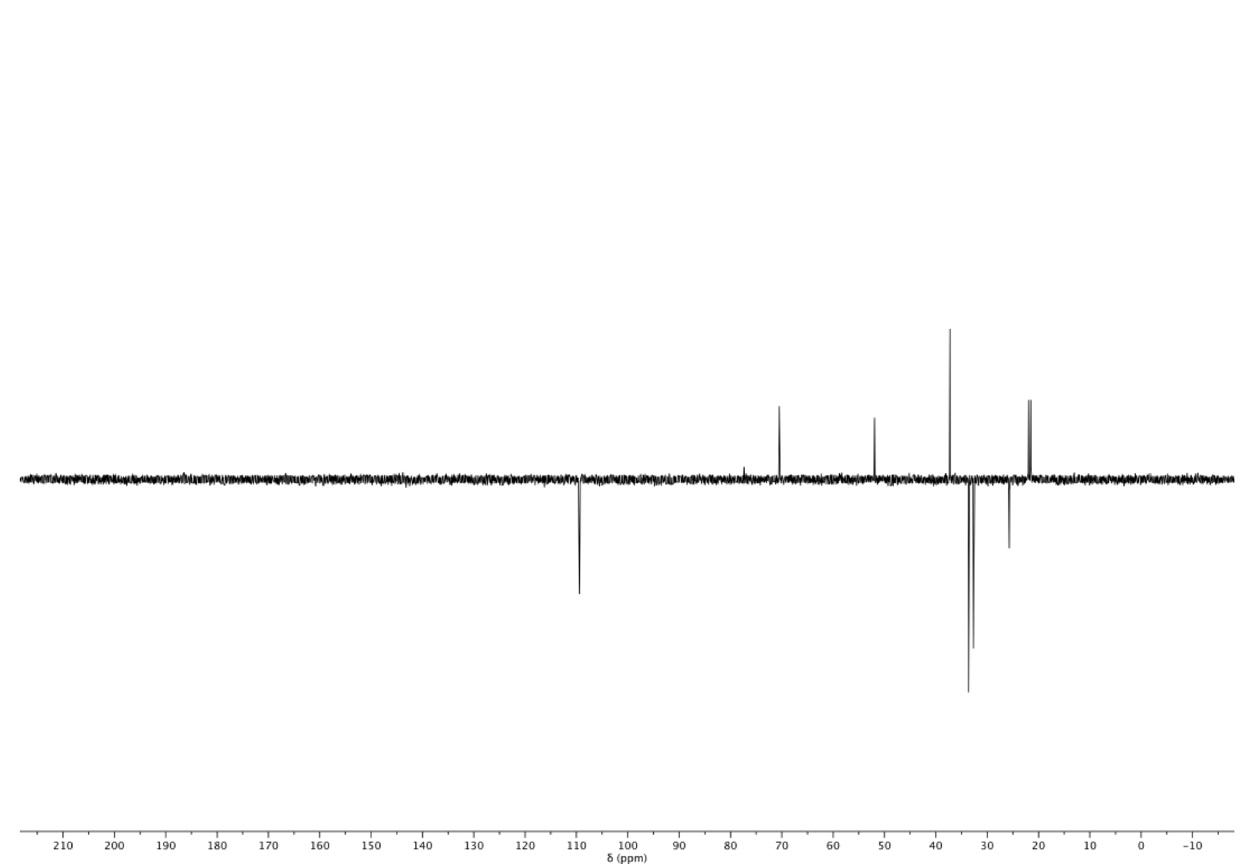


**Figure S14.** DEPT 135 spectrum of carbamate **3** in CDCl_3_.

**Figure S15.** ^1^H^13^C HSQC spectrum of carbamate **3** in CDCl_3_.

**Figure S16.** ^1^H^1^H COSY spectrum of carbamate **3** in CDCl_3_.

***(3aS,6R,7aS)-3a-methyl-6-(prop-1-en-2-yl)hexahydrobenzo[d]oxazol-2(3H)-one*** (**LU**):

A solution of hydroxyl carbamate **3** (5.00 g, 22.0 mmol, 1.00 eq.) in THF (110 mL, 0.20 M) was cooled to 0 °C, and potassium *tert*-butoxide (2.84 g, 25.3 mmol, 1.15 eq.) was added. After stirring for 1 hour , the mixture was refluxed for 16 hours. THF was removed by rotary evaporation and water (40 mL) was added. The aqueous solution was extracted with DCM (3 × 40 mL). The combined organic layers were dried over anhydrous sodium sulfate, filtered, and evaporated under vacuum. The crude product was purified *via* flash column chromatography on silica gel using diethyl ether/*n*-pentane (3:2) as eluent. The resulting crude product was sublimed at 10^-5^ mbar and 80 °C to get pure (3a*S*,6*R*,7a*S*)-3a-methyl-6-(prop-1-en-2-yl)hexahydrobenzo[*d*]oxazol-2(3*H*)-one (**LU**) (3.57 g, 18.3 mmol, 83%) as a colorless solid.

TLC: R_f_ = 0.56 (silica, diethyl ether) [KMnO_4_]

^1^H NMR (400 MHz, CDCl_3_) *δ* (ppm) = 5.14 (bs, 1H, N‑H), 4.95 (q, 1H, ^2^*J*_HH_ = 1.5 Hz, ^4^*J*_HH_ = 1.3 Hz, H‑11), 4.87 (d, 1H, ^2^*J*_HH_ = 1.5 Hz, H‑11), 4.12 (dd, 1H, ^3^*J*_HH_ = 13.2 Hz, 3.3 Hz, H‑1), 2.57 (t, 1H, ^3^*J*_HH_ = 6.5 Hz, H‑6), 2.20 (ddq, 1H, ^3^*J*_HH_ = 13.2 Hz, 3.3 Hz, 1.5 Hz, H‑7), 2.07‑1.95 (m, 1H, H‑5), 1.94‑1.88 (m, 1H, H‑4), 1.87‑1.82 (m, 1H, H‑7), 1.78 (s, 3H, H‑10), 1.77‑1.74 (m, 1H, H‑4), 1.74‑1.71 (m, 1H, H‑5), 1.25 (s, 3H, H‑8).

^13^C{^1^H} NMR (100 MHz, CDCl_3_) *δ* (ppm) = 160.55 (C‑2), 146.39 (C‑9), 111.69 (C‑11), 81.31 (C‑1), 60.47 (C‑3), 38.93 (C‑6), 32.55 (C‑4), 25.18 (C‑7), 24.46 (C‑5), 22.82 (C‑10), 18.42 (C‑8).

Elemental analysis: calc. for C_11_H_17_NO_2_: C, 67.66; H, 8.78; N, 7.17; O, 16.39; found: C, 67.66; H, 8.90; N, 7.09.

ESI-MS: m/z = calc. for [C_11_H_17_NO_2_]^+^: 195.1259 ([M]^+^); found 195.1259.

GC-MS: t_R_ = 13.473 min, m/z = 195.1 ([M]^+^), 180.1 ([M-CH_3_]^+^).

**
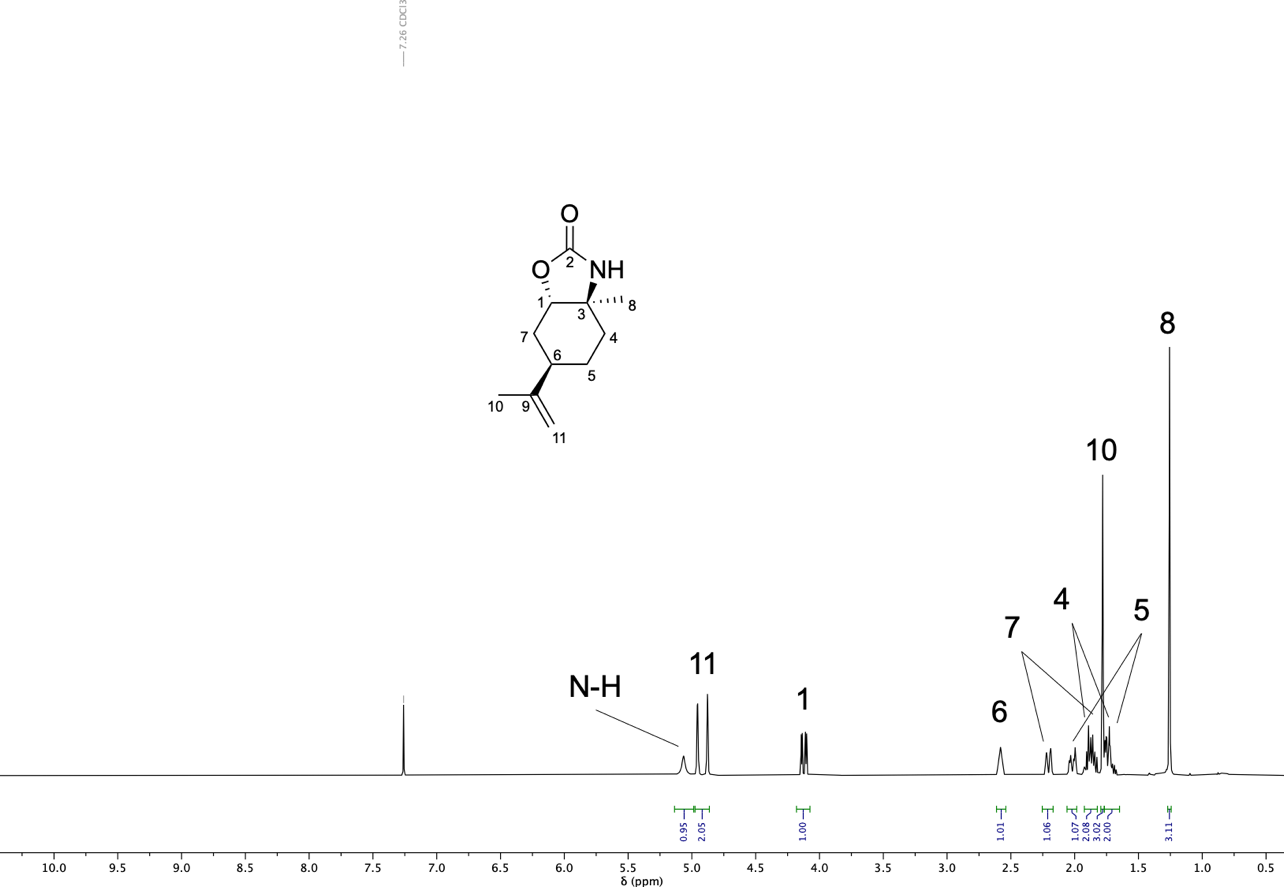
**

**Figure S17.** ^1^H NMR spectrum of monomer **LU** in CDCl_3_.


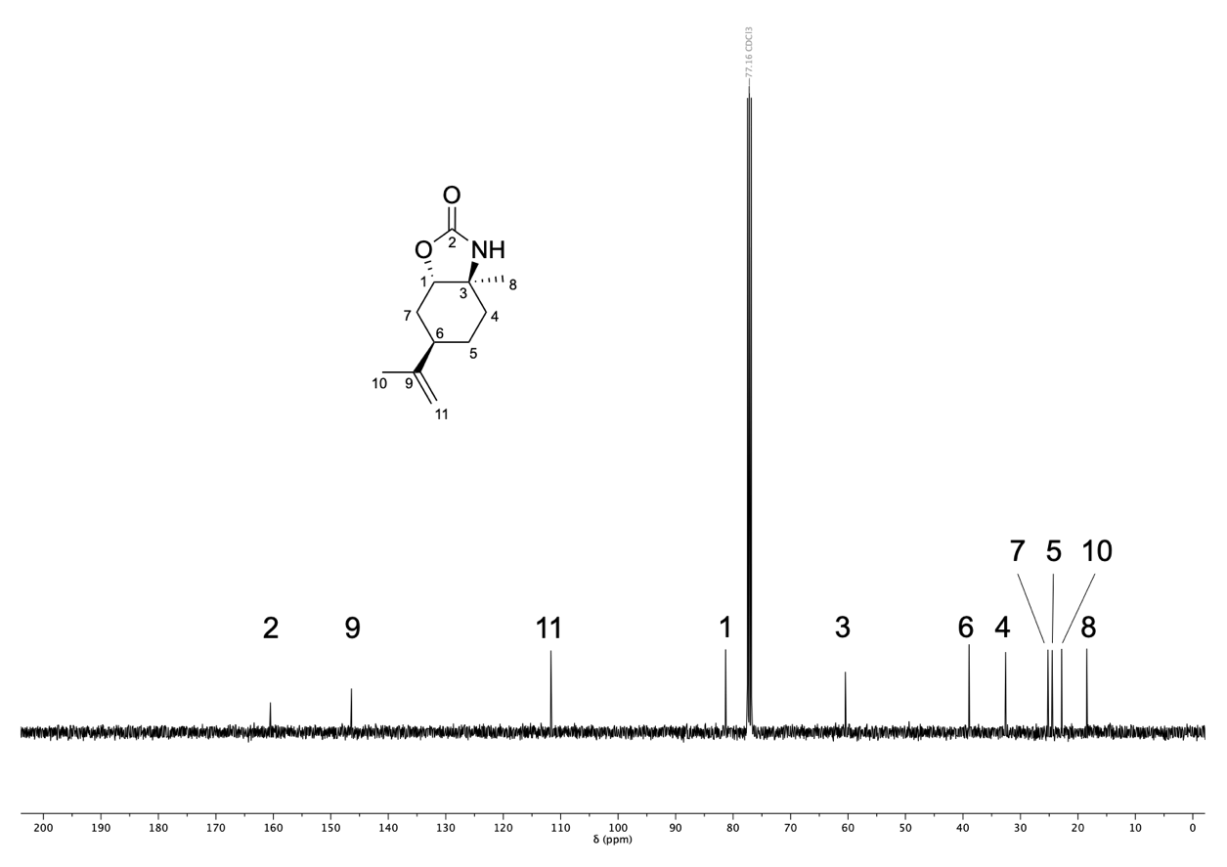


**Figure S18.** ^13^C{^1^H} NMR spectrum of monomer **LU** in CDCl_3_.


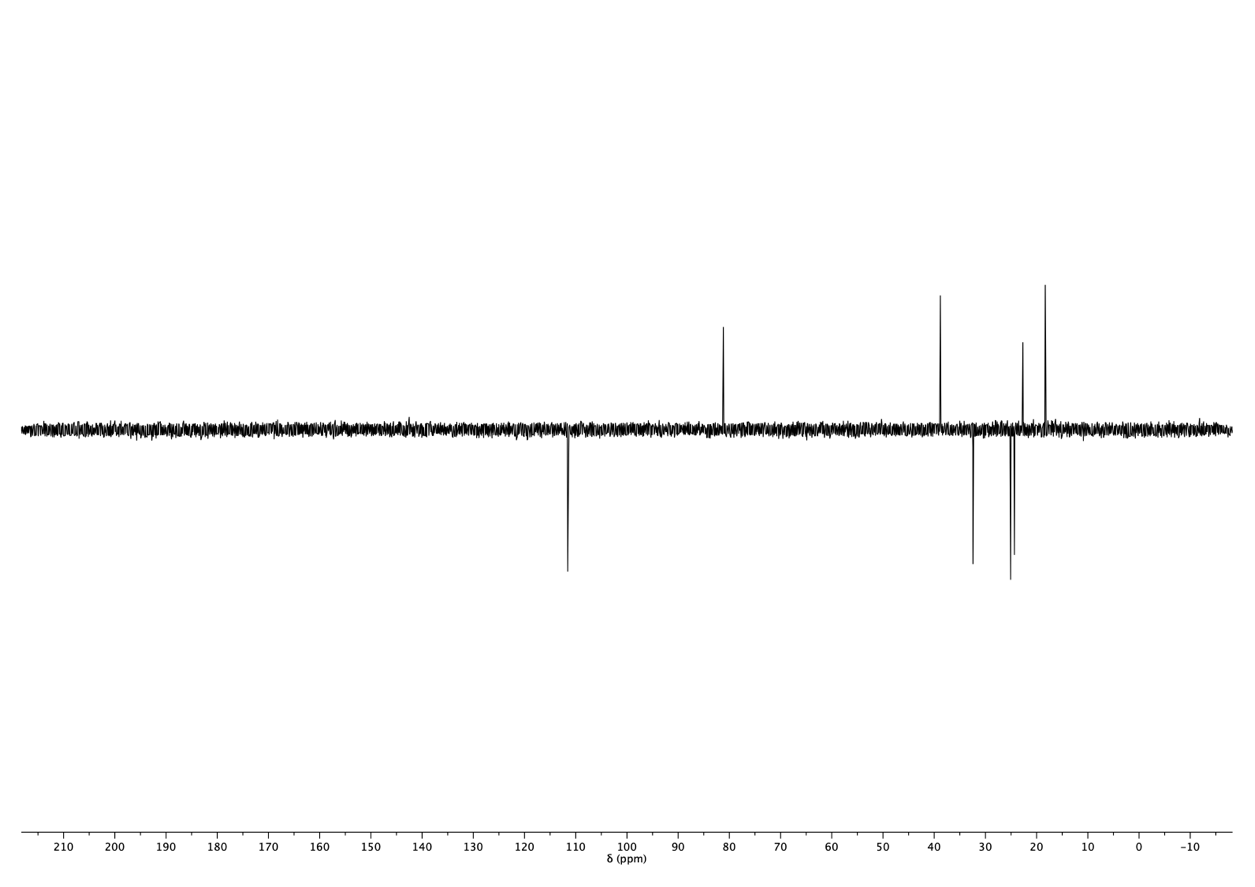


**Figure S19.** DEPT 135 spectrum of monomer **LU** in CDCl_3_.

**Figure S20.** ^1^H^13^C HSQC spectrum of monomer **LU** in CDCl_3_.

**Figure S21.** ^1^H^1^H COSY spectrum of monomer **LU** in CDCl_3_.


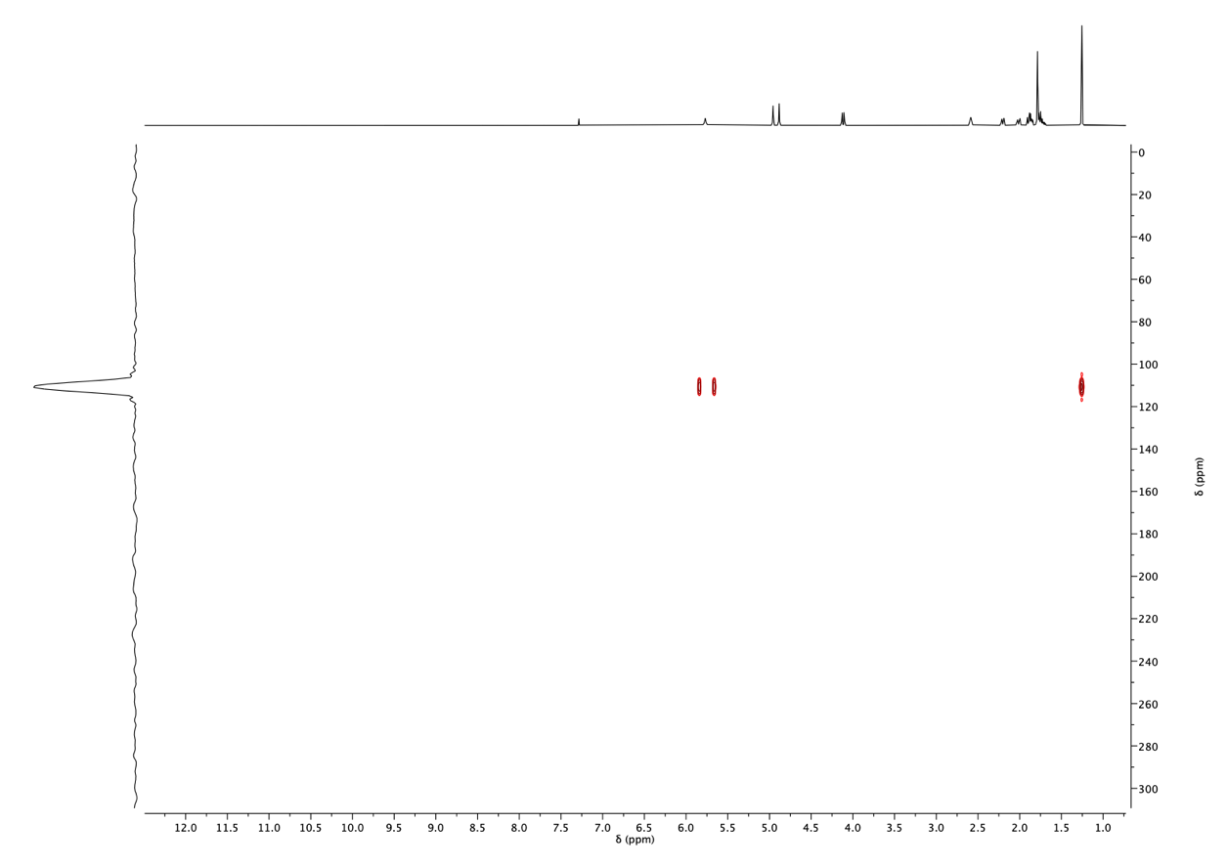


**Figure S22.** ^1^H^15^N HMBC spectrum of monomer **LU** in CDCl_3_.

Alternative attempts to produce LU in a shorter synthesis pathway were realized by reacting amino alcohol 2 with CO_2_ and by reacting epoxide 1 with potassium cyanate. However, none of the synthesis yielded the desired product. Both procedures are described in the following:

*cis*-(*R*)-Limonene epoxide **1** (1.00 g, 6.57 mmol, 1.00 eq.) was dissolved in methanol (3 mL, 2.20 M), and potassium cyanate (1.07 g, 13.1 mmol, 2.00 eq.), and ammonium chloride (351 mg, 6.57 mmol, 1.00 eq.) were added. After refluxing for 6 days, the mixture was cooled to room temperature and analyzed by GC. Only low conversion was observed, and carbamate **3** was obtained in 24% instead of monomer **LU**. The procedure was adapted to a protocol by *Palmieri et al.*.^[7]^

In an 100 mL autoclave, amino alcohol **2** (1.00 g, 5.91 mmol, 1.00 eq.) was dissolved in acetonitrile (28 mL, 0.20 M) and CeO_2_ (102 mg, 591 µmol, 0.10 eq.) was added. The mixture was pressurized with 50 bar CO_2_ and heated to 170 °C for 20 hours. After cooling to room temperature, GC analysis revealed no conversion. The procedure was adapted from *Tomishige et al.*.^[8]^

*(3aS,6R,7aS)-3-benzoyl-3a-methyl-6-(prop-1-en-2-yl)hexahydrobenzo[d]oxazol-2(3H)-one* (A1): *n*-Butyl lithium (2.50 M in *n*-hexane, 360 μL, 901 μmol, 1.10 eq.) was added to a stirred solution of (3a*S*,6*R*,7a*S*)-3a-methyl-6-(prop-1-en-2-yl)hexahydro-benzo[*d*]oxazol-2(3*H*)-one (LU) (160 mg, 819 μmol, 1.00 eq.) in THF (3 mL, 0.27 M) at –78 °C. After 30 minutes benzoyl chloride (138 mg, 983 μmol, 1.20 eq.) was slowly added. The mixture was stirred for 30 minutes and then slowly heated to room temperature. After 16 hours THF was removed by rotary evaporation and water (5 mL) was added. The aqueous solution was extracted with DCM (3 × 5 mL). The combined organic layers were washed with brine (5 mL) dried over anhydrous sodium sulfate, filtered and evaporated under vacuum. The crude product was purified *via* flash column chromatography on silica gel using *n*‑pentane/ethyl acetate (4:1) as eluent. Recrystallization in *n*‑hexane/ethyl acetate (1:1) gave pure (3a*S*,6*S*,7a*S*)-3-benzoyl-3a-methyl-6-(prop-1-en-2-yl)hexahydrobenzo[*d*]oxazol-2(3*H*)-one (A1) (202 mg, 675 μmol, 82%) as a colorless solid.

TLC: R_f_ = 0.56 (silica, *n*-pentane/ethyl acetate 4:1) [UV]

^1^H NMR (400 MHz, CDCl_3_) *δ* (ppm) = 7.68‑7.65 (m, 2H, H‑14, H‑18), 7.56 (ddt, 1H, ^3^*J*_HH_ = 8.5 Hz, 7.0 Hz, ^4^*J*_HH_ = 1.5 Hz, H‑16), 7.44 (dd, 2H, ^3^*J*_HH_ = 8.5 Hz, 7.0 Hz, H‑15, H‑17), 5.04 (q, 1H, ^2^*J*_HH_ = 1.4 Hz, H‑11), 4.95 (s, 1H, H‑11), 4.26 (dd, 1H, ^3^*J*_HH_ = 13.2 Hz, 3.5 Hz, H‑1), 2.66 (t, 1H, ^3^*J*_HH_ = 6.1 Hz, H‑6), 2.58‑2.48 (m, 1H, H‑4), 2.30 (ddt, 1H, ^3^*J*_HH_ = 13.2 Hz, 3.5 Hz, H‑7), 2.13 (dt, 1H, ^3^*J*_HH_ = 14.5 Hz, 2.1 Hz, H‑5), 2.01 (dd, 1H, ^3^*J*_HH_ = 13.2 Hz, 6.1 Hz, H‑7), 1.98‑1.93 (m, 1H, H‑4), 1.92‑1.87 (m, 1H, H‑5), 1.85 (s, 3H, H‑10), 1.57 (s, 3H, H‑8).

^13^C{^1^H} NMR (100 MHz, CDCl_3_) *δ* (ppm) = 170.27 (C‑12), 154.58 (C‑2), 146.05 (C‑9), 133.90 (C‑13), 132.58 (C‑16), 129.13 (C‑14, C‑18), 128.12 (C‑15, C‑17), 111.97 (C‑11), 80.71 (C‑1), 65.63 (C‑3), 38.53 (C‑6), 30.95 (C‑4), 24.66 (C‑7), 24.07 (C‑5), 22.79 (C‑10), 14.69 (C‑8).

Elemental analysis: calc. for C_18_H_21_NO_3_: C, 72.22; H, 7.07; N, 4.68; O, 16.03; found: C, 72.34; H, 6.98; N, 4.72.

ESI-MS: m/z = calc. for [C_18_H_21_NO_3_]^+^: 299.1599 ([M+H]^+^); found 300.1592.

GC-MS: t_R_ = 17.278 min, m/z = 299.2 ([M]^+^).


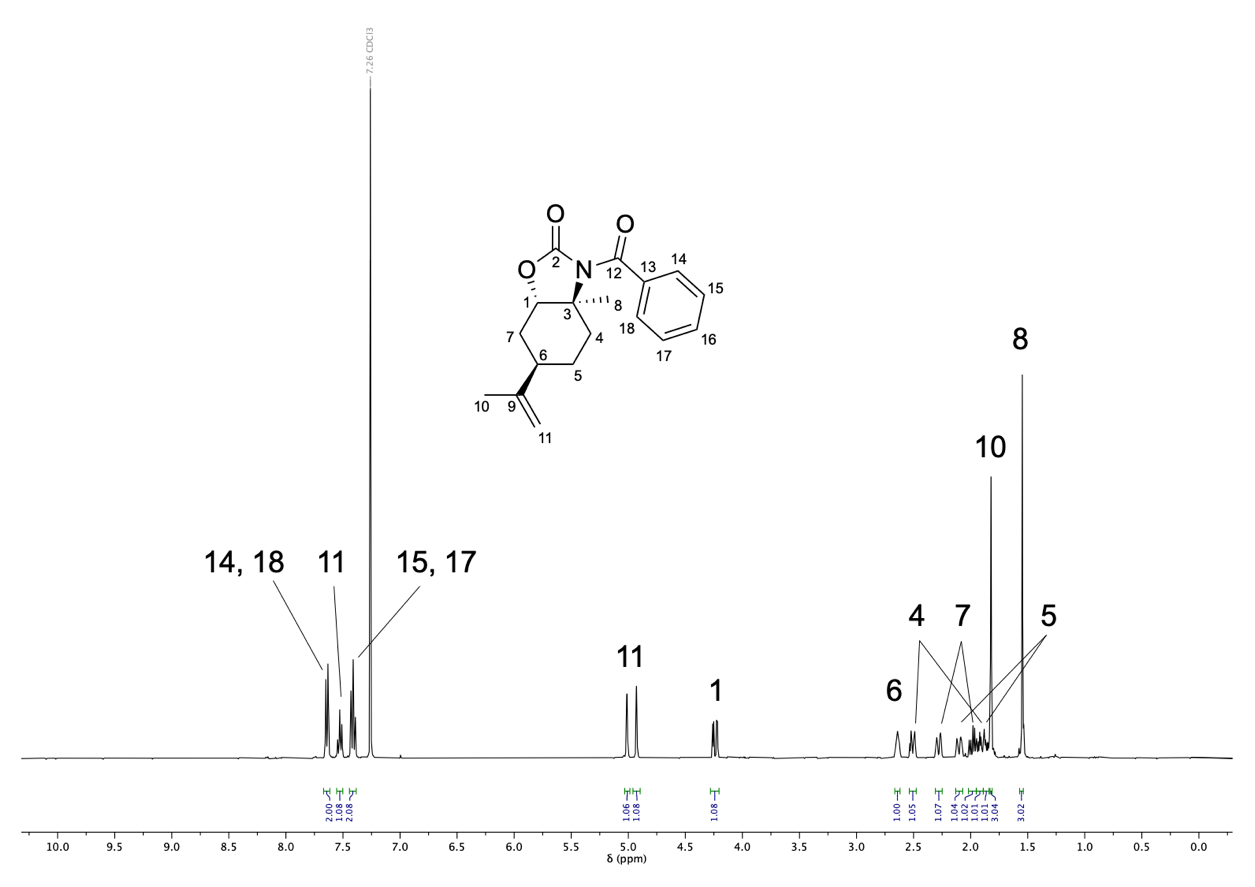


**Figure S23.** ^1^H NMR spectrum of activator **A1** in CDCl_3_.


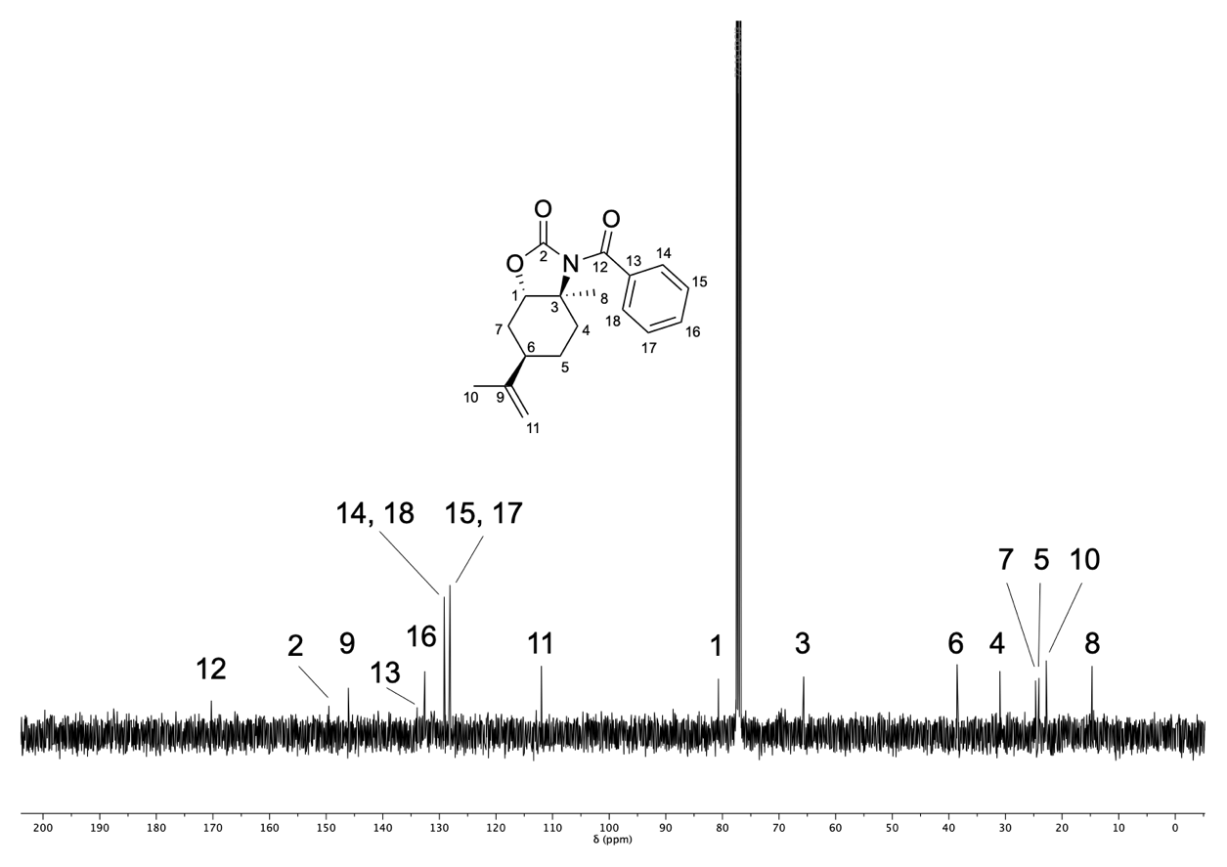


**Figure S24.** ^13^C{^1^H} NMR spectrum of activator **A1** in CDCl_3_.


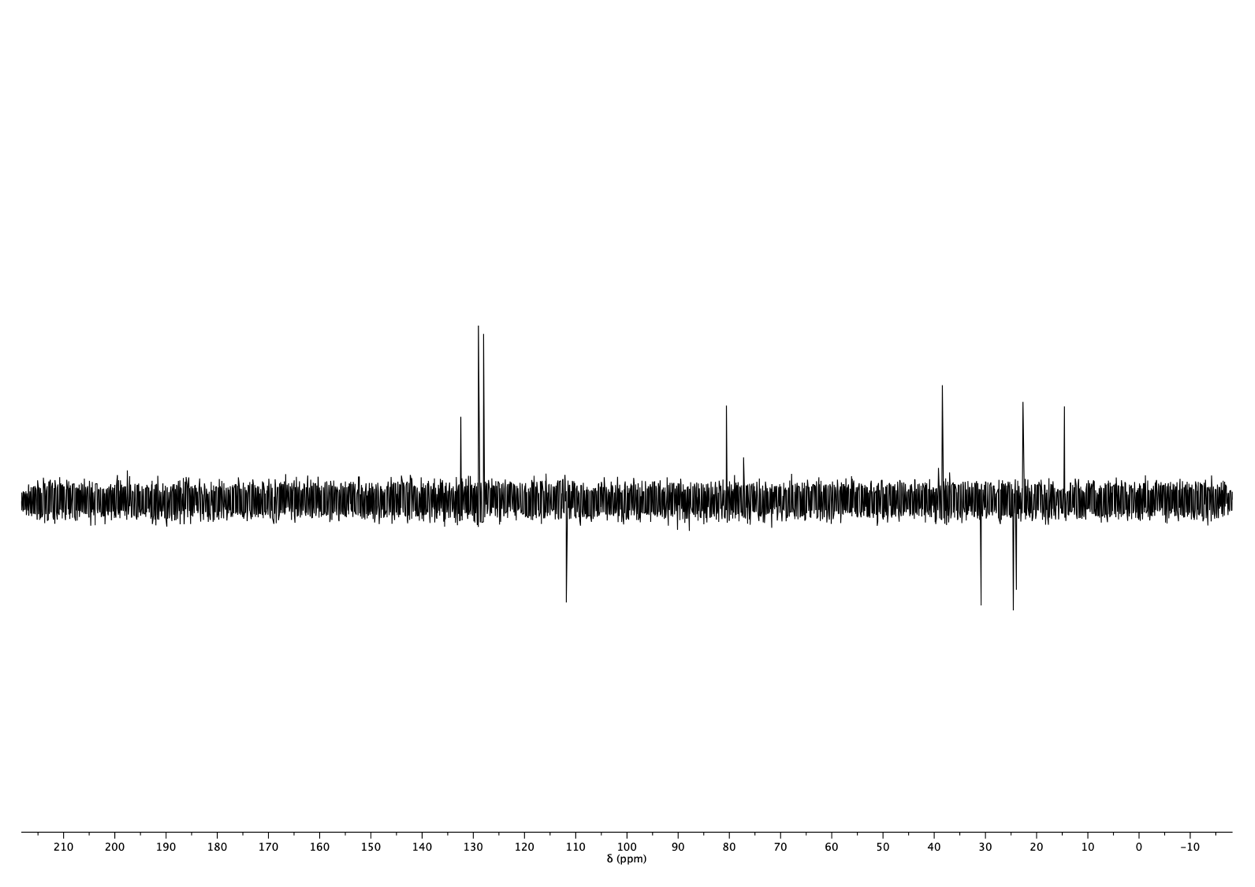


**Figure S25.** DEPT 135 spectrum of activator **A1** in CDCl_3_.


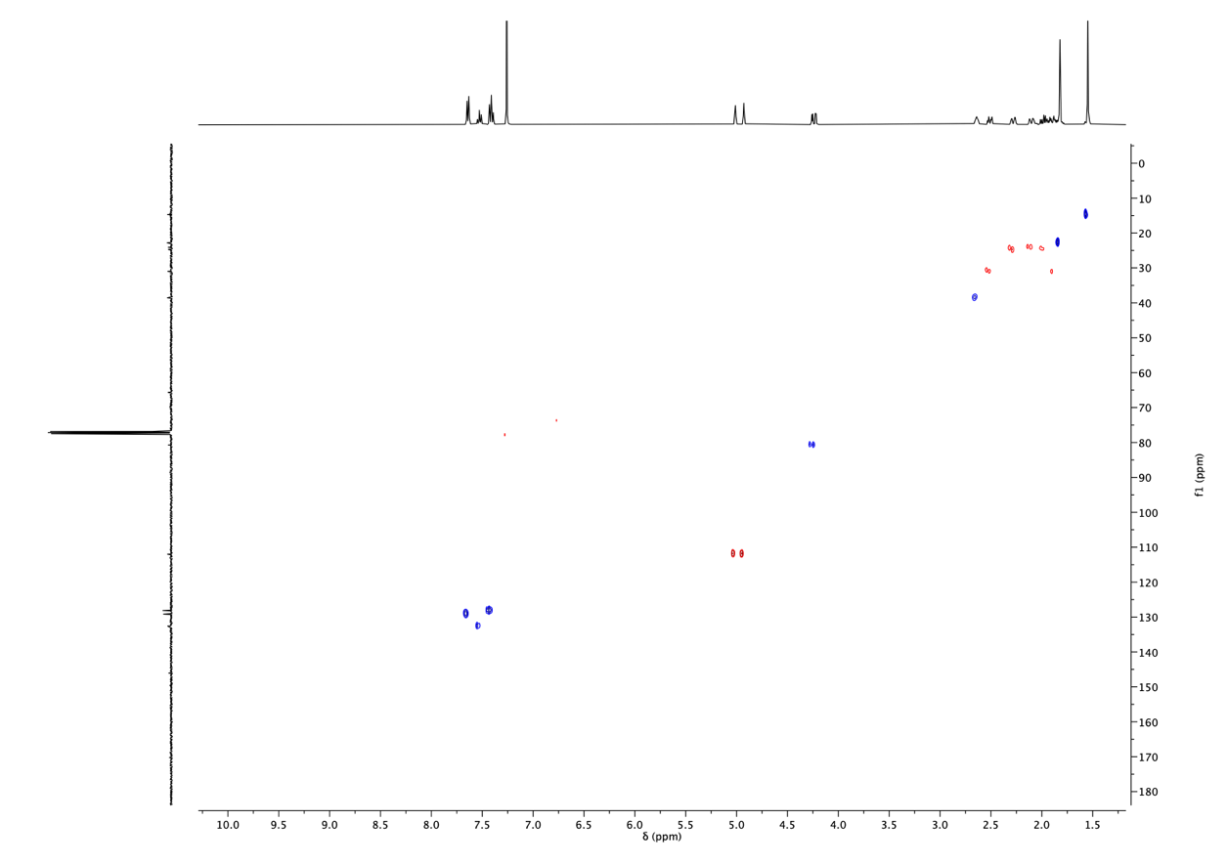


**Figure S26.** ^1^H^13^C HSQC spectrum of activator **A1** in CDCl_3_.

**
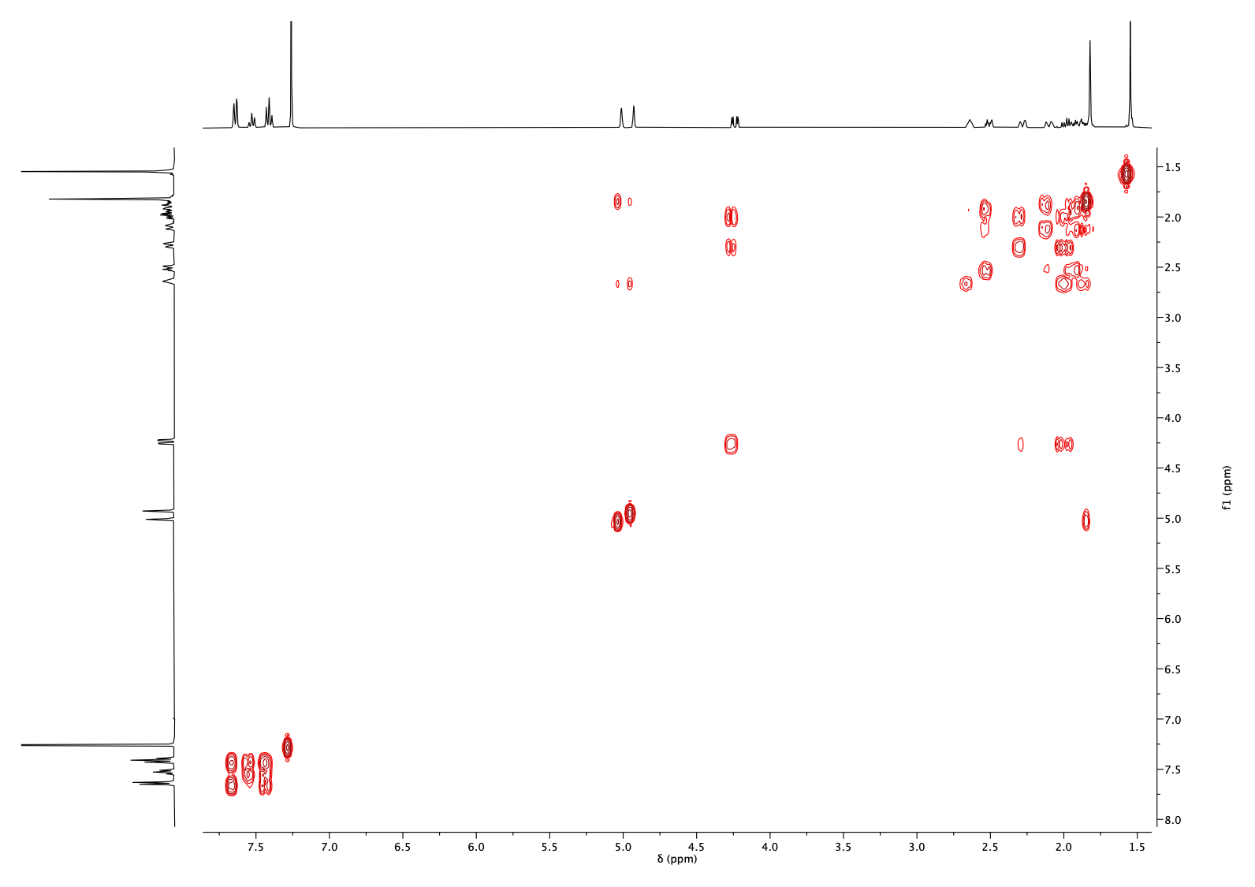
**

**Figure S27.** ^1^H^1^H COSY spectrum of activator **A1** in CDCl_3_.


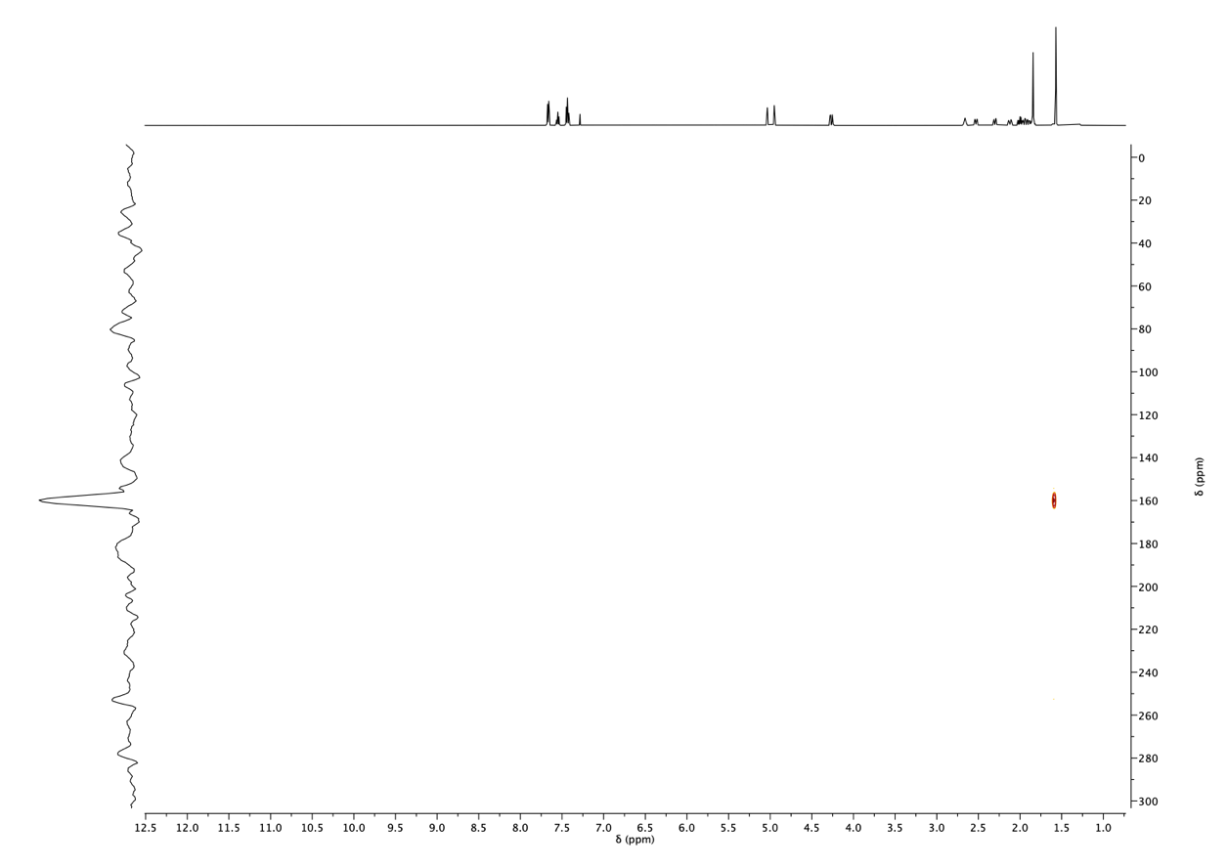


**Figure S28.** ^1^H^15^N HMBC spectrum of activator **A1** in CDCl_3_.

***(3aS,6R,7aS)-3a-methyl-6-(prop-1-en-2-yl)hexahydrobenzo[d]oxazol-2(3H)-one-3-d*** (***d***-**LU**):

*n*-Butyl lithium (2.50 M in *n*-hexane, 789 µL, 1.97 mmol, 1.10 eq.) was added to a stirred solution of (3aS,6*R*,7a*S*)-3a-methyl-6-(prop-1-en-2-yl)hexahydrobenzo[*d*]oxazol-2(3*H*)-one (**LU**) (350 mg, 1.79 mmol, 1.00 eq.) in THF (5 mL, 0.35 M) at –78 °C. After 45 minutes D_2_O (3.60 g, 180 mmol, 100 eq.) was added. The mixture was slowly heated to room temperature and stirred for 20 minutes. THF was removed by rotary evaporation, and the aqueous solution was extracted with DCM (3 × 5 mL). The combined organic layers were dried over anhydrous sodium sulfate, filtered and evaporated under vacuum. This whole procedure was repeated twice to ensure high deuteration. The resulting crude product was sublimed at 10^-5^ mbar and 80 °C to get pure (3a*S*,6*R*,7a*S*)-3a-methyl-6-(prop-1-en-2-yl)hexahydrobenzo[*d*]oxazol-2(3*H*)-one-3-*d* (***d***-**LU**) (332 mg, 1.69 mmol, 94%) as a colorless solid.

^1^H NMR (400 MHz, CD_2_Cl_2_) *δ* (ppm) = 4.95 (q, 1H, ^2^*J*_HH_ = 1.7 Hz, ^4^*J*_HH_ = 1.5 Hz, H‑11), 4.89 (d, 1H, ^2^*J*_HH_ = 1.5 Hz, H‑11), 4.09 (dd, 1H, ^3^*J*_HH_ = 13.5 Hz, 3.5 Hz, H‑1), 2.58 (t, 1H, ^3^*J*_HH_ = 6.8 Hz, H‑6), 2.21‑2.15 (m, 1H, H‑7), 2.04‑1.96 (m, 1H, H‑5), 1.91‑1.82 (m, 2H, H‑4, H‑7), 1.79 (s, 3H, H‑10), 1.77‑1.74 (m, 1H, H‑4), 1.74‑1.71 (m, 1H, H‑5), 1.23 (s, 3H, H‑8).

^13^C{^1^H} NMR (100 MHz, CD_2_Cl_2_) *δ* (ppm) = 160.47 (C‑2), 147.13 (C‑9), 111.47 (C‑11), 81.47 (C‑1), 60.47 (C‑3), 39.28 (C‑6), 32.83 (C‑4), 25.42 (C‑7), 24.67 (C‑5), 22.81 (C‑10), 18.34 (C‑8).


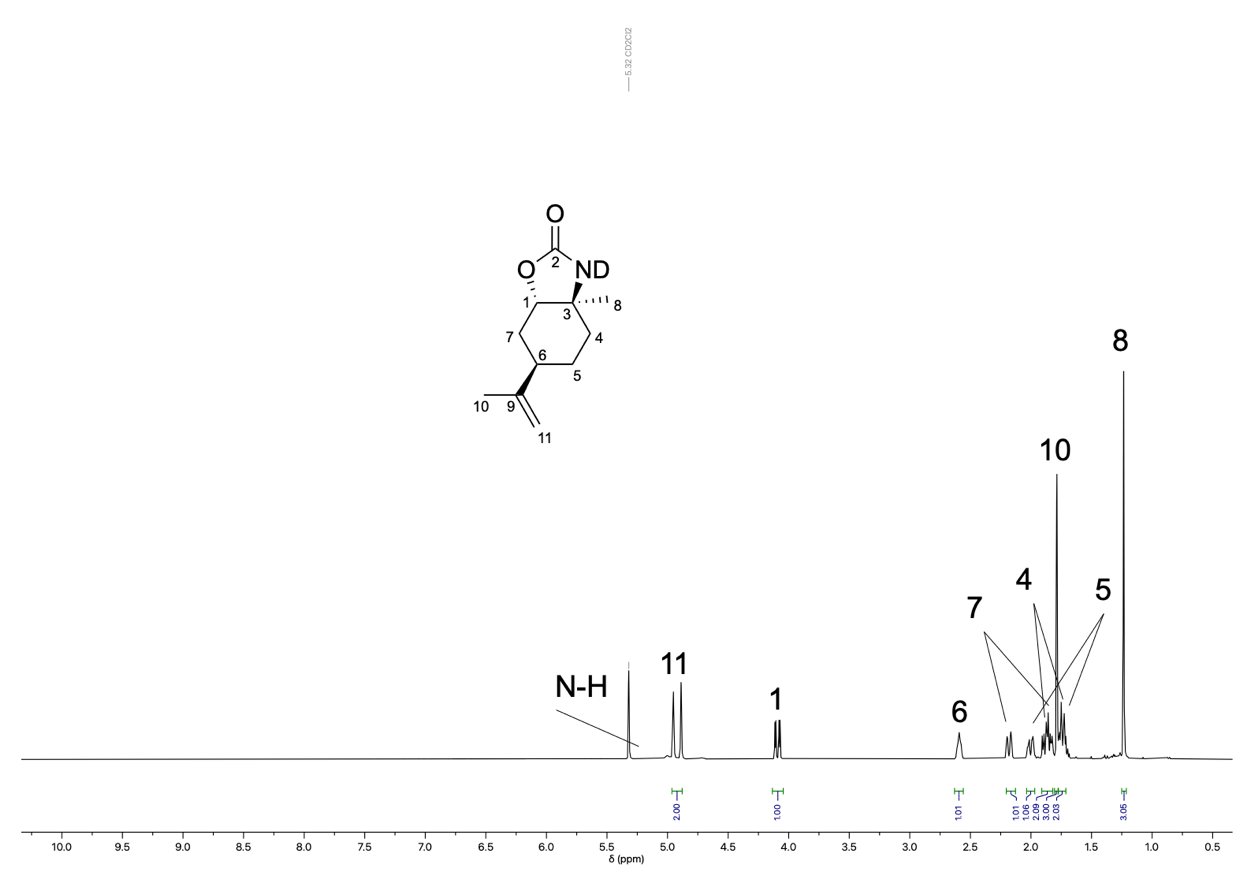


**Figure S29.** ^1^H NMR spectrum of monomer ***d***-**LU** in CD_2_Cl_2_.


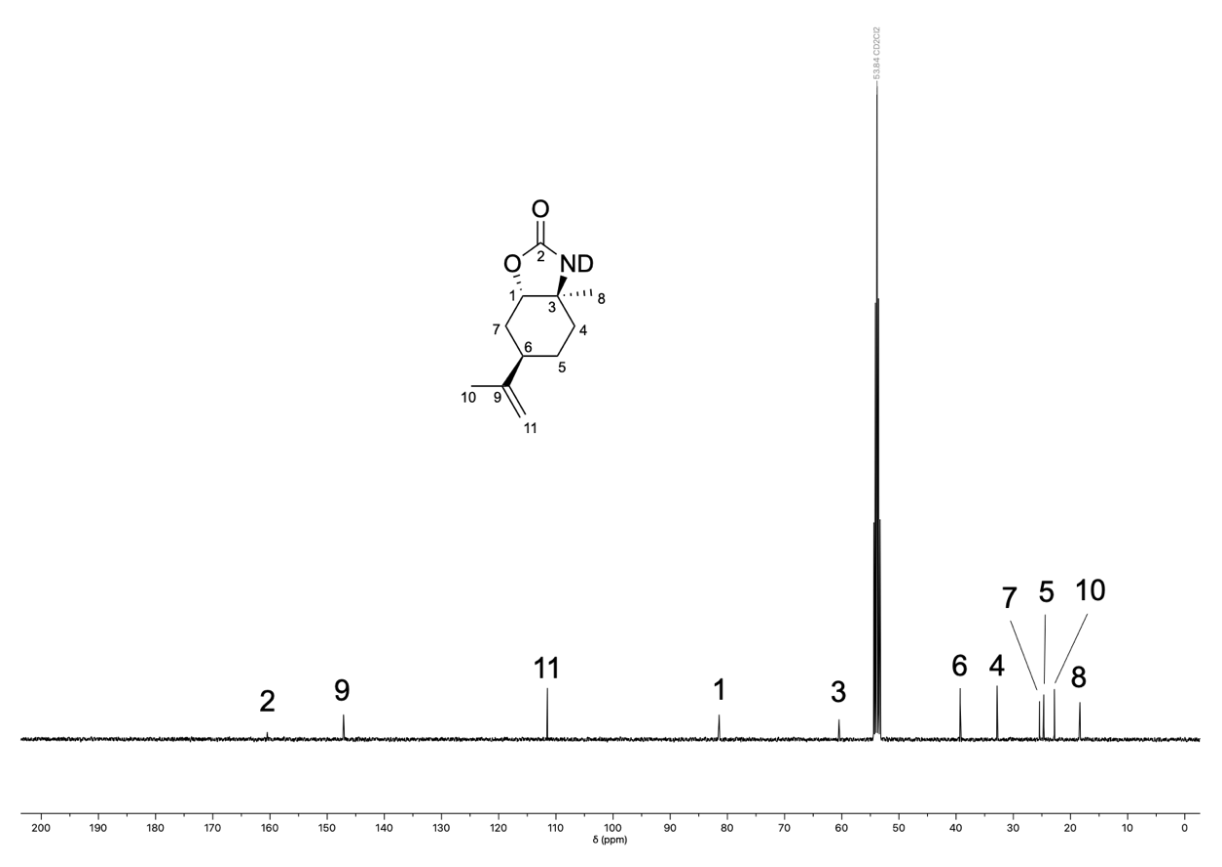


**Figure S30.** ^13^C{^1^H} NMR spectrum of monomer ***d***-**LU** in CD_2_Cl_2_.


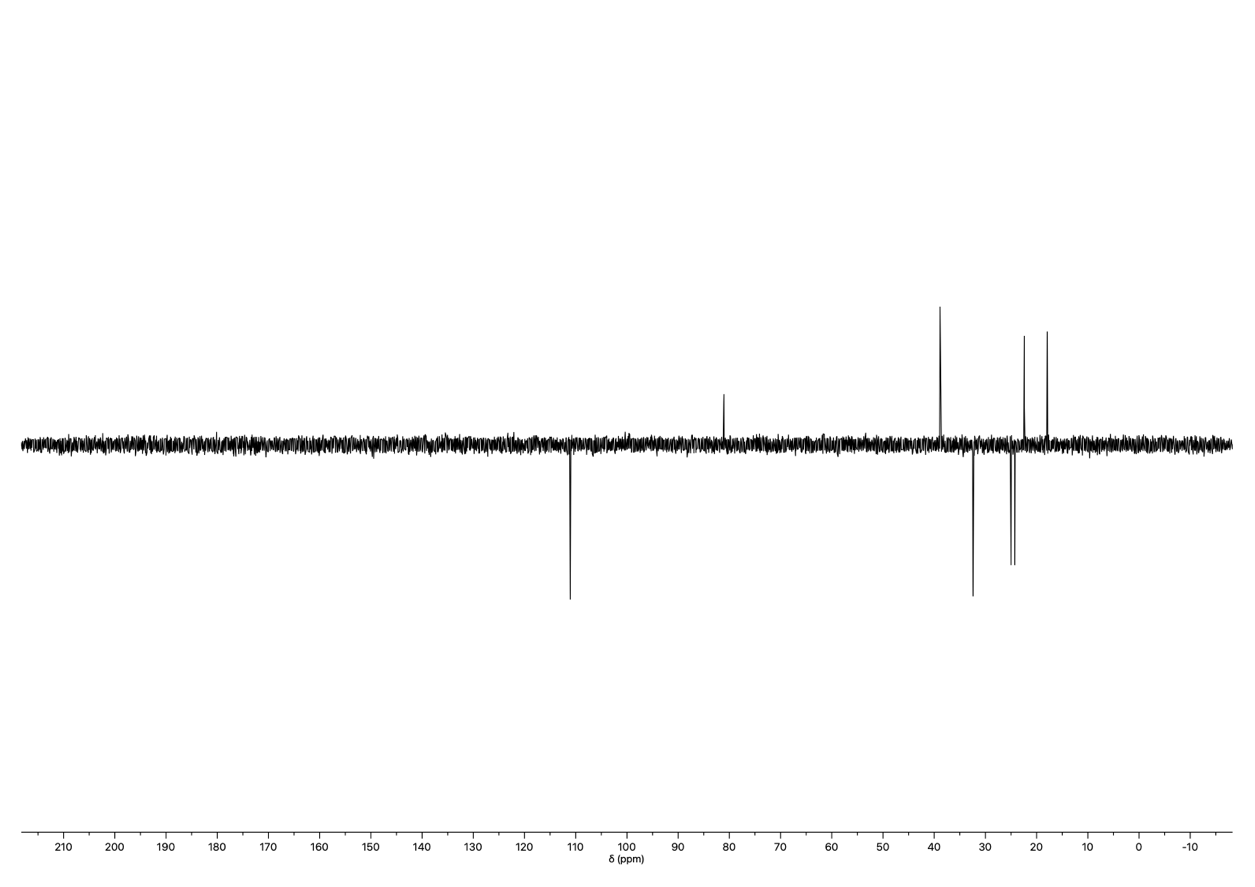


**Figure S31.** DEPT 135 spectrum of monomer ***d***-**LU** in CD_2_Cl_2_.


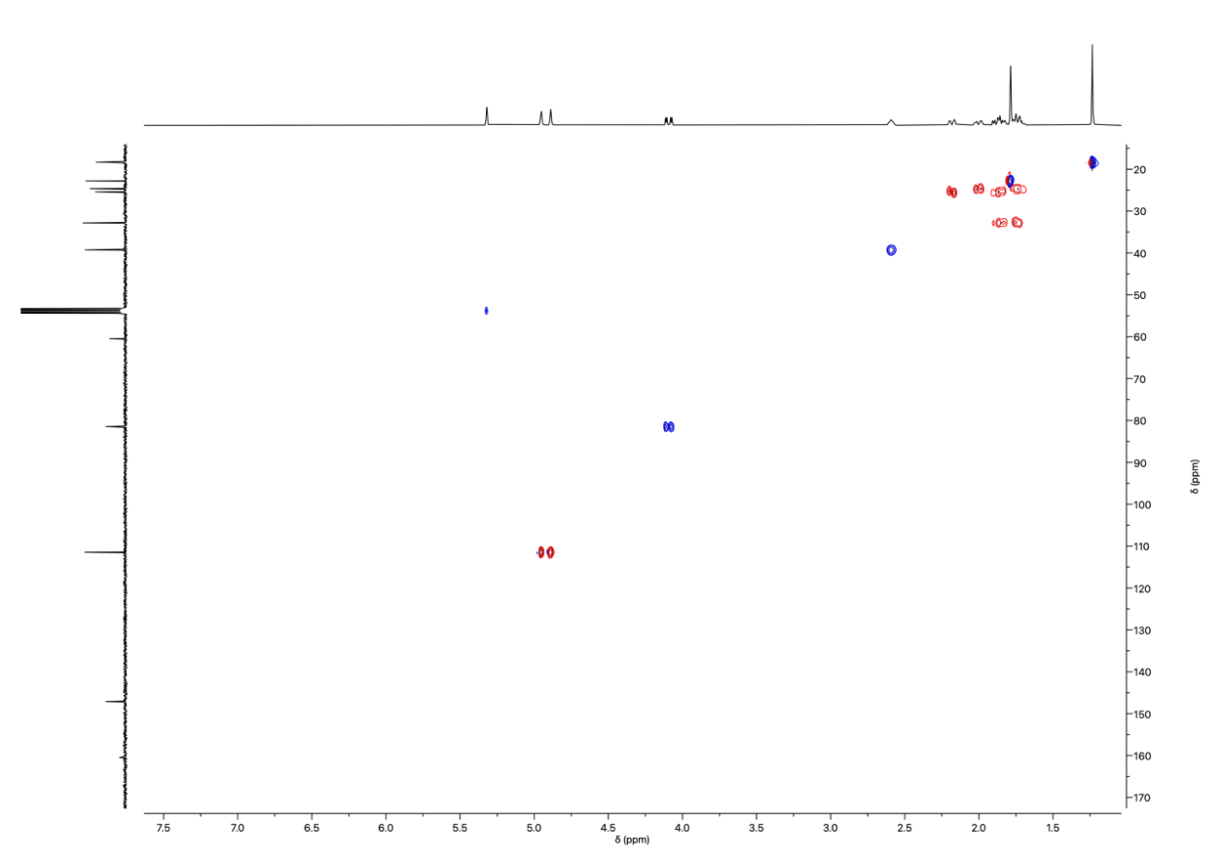


**Figure S32.** ^1^H^13^C HSQC spectrum of monomer ***d***-**LU** in CD_2_Cl_2_.


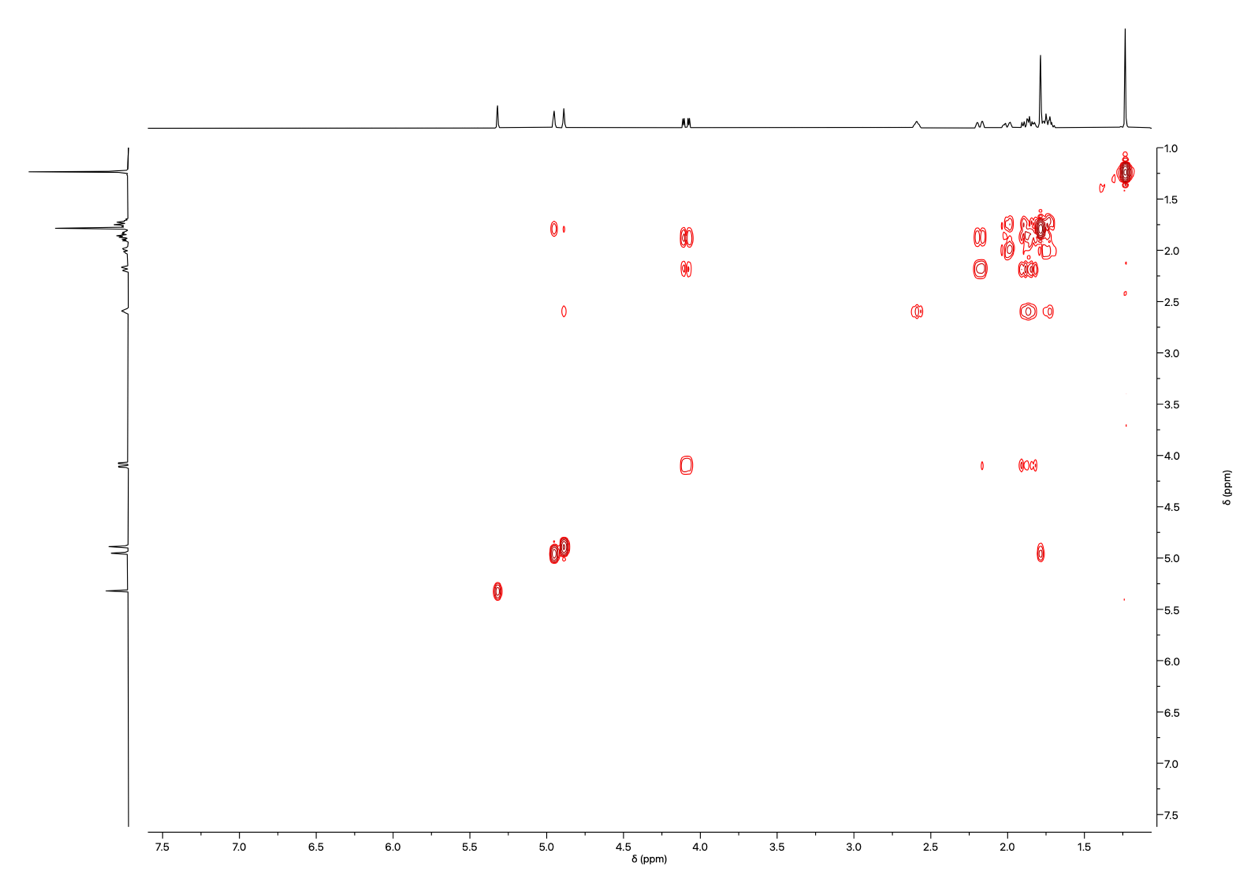


**Figure S33.** ^1^H^1^H COSY spectrum of monomer ***d***-**LU** in CD_2_Cl_2_.

*(3aS,6R,7aS)-3,3a-dimethyl-6-(prop-1-en-2-yl)hexahydrobenzo[d]oxazol-2(3H)-one* (Me-LU):

*n*-Butyl lithium (2.50 M in *n*-hexane, 2.25 mL, 5.63 mmol, 1.10 eq.) was added to a stirred solution of (3a*S*,6*R*,7a*S*)-3a-methyl-6-(prop-1-en-2-yl)hexahydrobenzo[*d*]oxazol-2(3*H*)-one (LU) (1.00 g, 5.12 mmol, 1.00 eq.) in THF (15 mL, 0.34 M) at –78 °C. After 30 minutes iodomethane (800 mg, 5.63 mmol, 1.10 eq.) was slowly added. The mixture was stirred for 30 minutes and then slowly heated to room temperature. After 16 hours THF was removed by rotary evaporation and water (25 mL) was added. The aqueous solution was extracted with DCM (3 × 25 mL). The combined organic layers were washed with brine (25 mL) dried over anhydrous sodium sulfate, filtered and evaporated under vacuum. The crude product was purified *via* flash column chromatography on silica gel using *n*-pentane/ethyl acetate (2:3) as eluent. (3a*S*,6*R*,7a*S*)-3,3a-dimethyl-6-(prop-1-en-2-yl)hexahydro-benzo[*d*]oxazol-2(3*H*)-one
(Me-LU) (556 mg, 2.66 mmol, 52%) was obtained as a colorless solid.

TLC: R_f_ = 0.47 (silica, *n*-pentane/ethyl acetate 3:2) [KMnO_4_]

^1^H NMR (400 MHz, CDCl_3_) *δ* (ppm) = 4.96‑4.93 (m, 1H, H‑11), 4.86 (s, 1H, H‑11), 3.96 (dd, 1H, ^3^*J*_HH_ = 13.2 Hz, 3.5 Hz, H‑1), 2.71 (s, 3H, H‑12), 2.60 (s, 1H, H‑6), 2.19 (ddd, 1H, ^3^*J*_HH_ = 13.2 Hz, 3.5 Hz, ^2^*J*_HH_ = 1.9 Hz, H‑7), 2.08‑1.99 (m, 1H, H‑5), 1.89 (td, 1H, ^3^*J*_HH_ = 13.2 Hz, 6.2 Hz, H‑7), 1.78 (s, 3H, H‑10), 1.77‑1.69 (m, 3H, 2 × H‑4, H-5), 1.13 (s, 3H, H‑8).

^13^C{^1^H} NMR (100 MHz, CDCl_3_) *δ* (ppm) = 159.37 (C‑2), 146.53 (C‑9), 111.58 (C‑11), 79.88 (C‑1), 62.63 (C‑3), 39.11 (C‑6), 31.16 (C‑4), 26.35 (C‑12), 25.07 (C‑7), 24.11 (C‑5), 22.83 (C‑10) , 13.04 (C‑8).

Elemental analysis: calc. for C_12_H_19_NO_2_: C, 68.87; H, 9.15; N, 6.69; O, 15.29; found: C, 68.70; H, 9.07; N, 6.73.

ESI-MS: m/z = calc. for [C_12_H_19_NO_2_]^+^: 209.1415 ([M]^+^); found 209.1418.

GC-MS: t_R_ = 13.518 min, m/z = 209.1 ([M]^+^), 194.1 ([M-CH_3_]^+^).

**
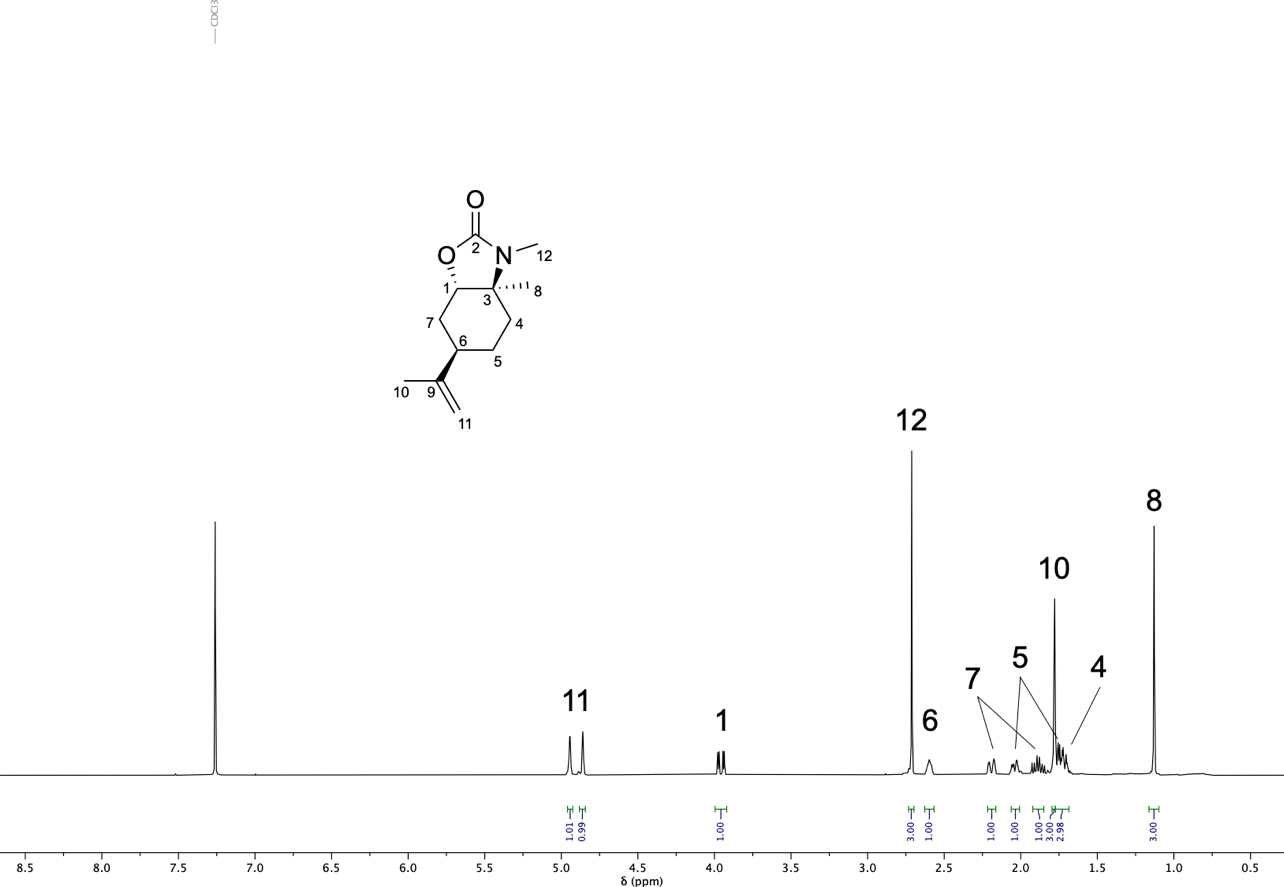
**

**Figure S34.** ^1^H NMR spectrum of monomer **Me-LU** in CDCl_3_.

**
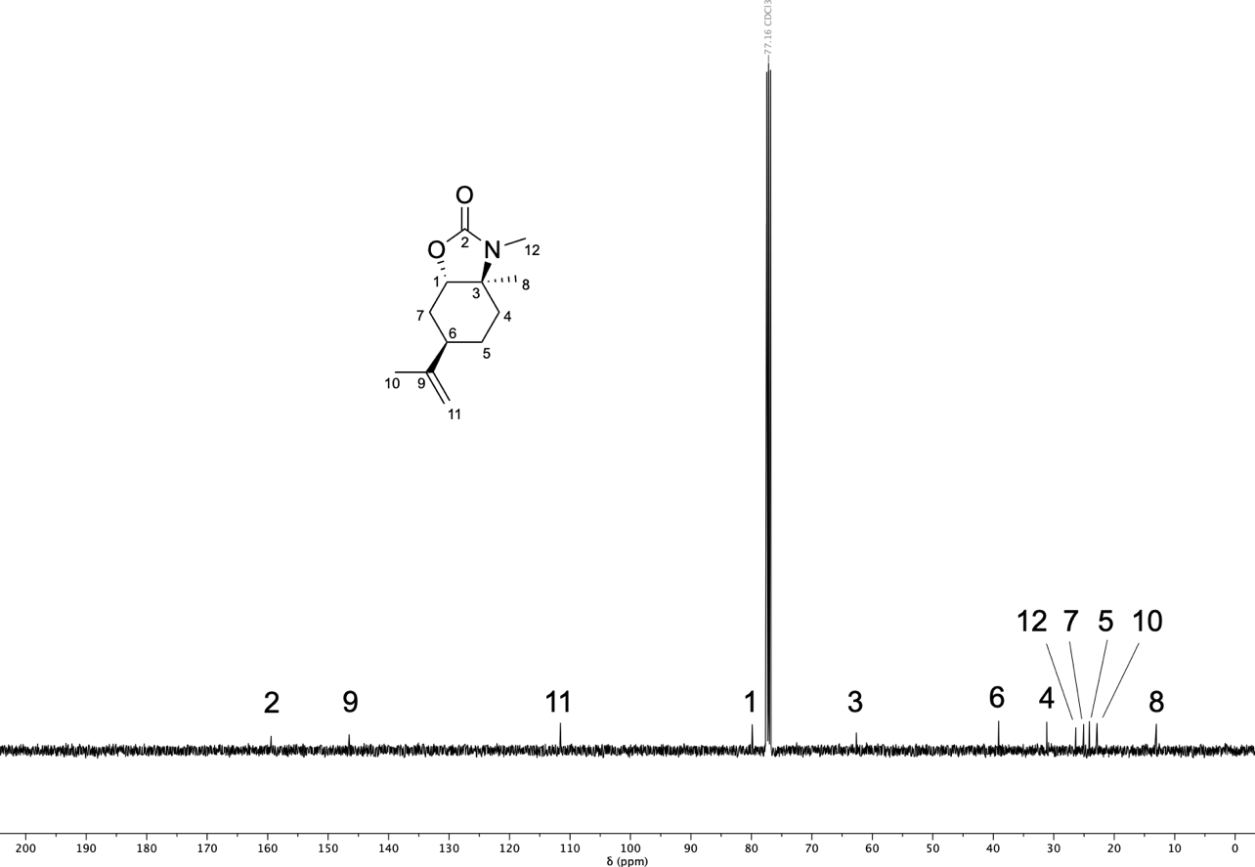
**

**Figure S35.** ^13^C{^1^H} NMR spectrum of monomer **Me-LU** in CDCl_3_.


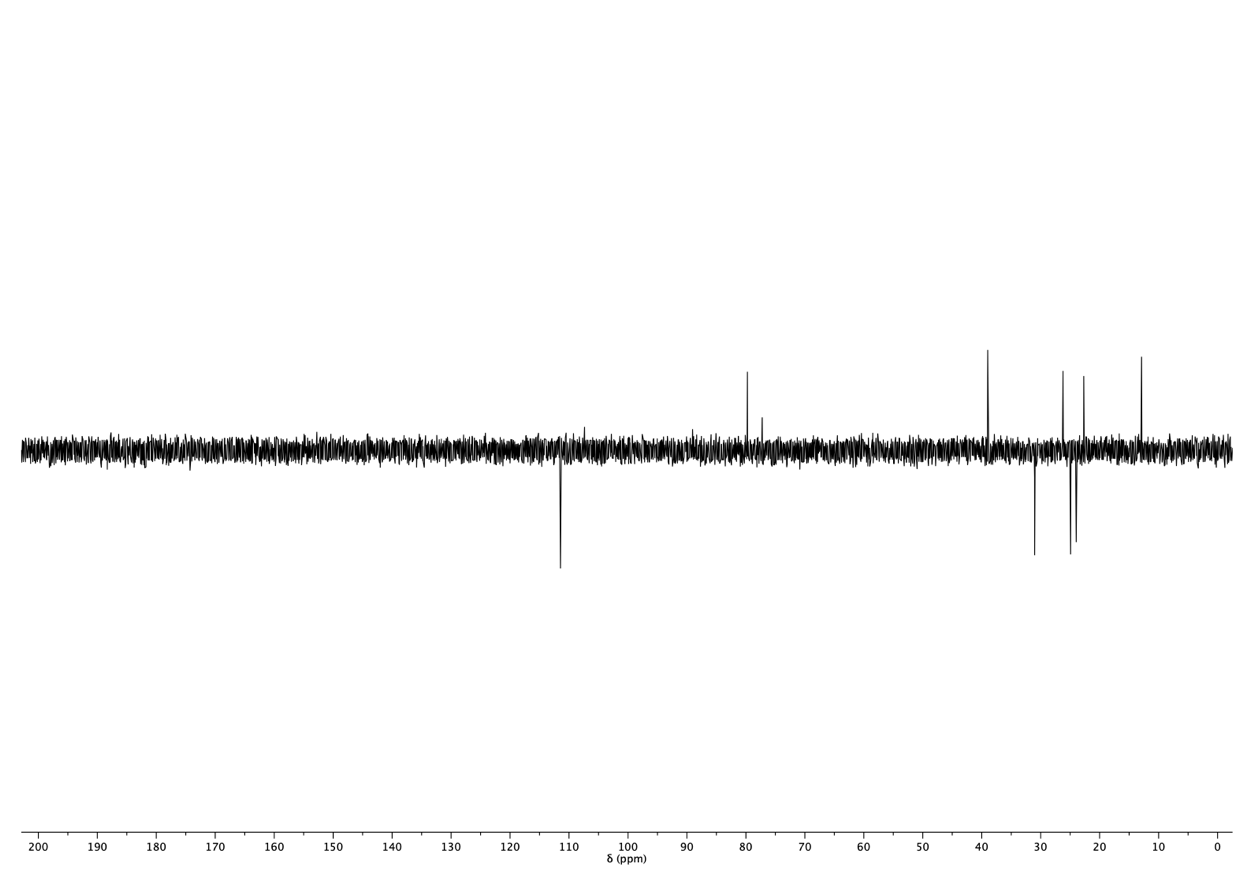


**Figure S36.** DEPT 135 spectrum of monomer **Me-LU** in CDCl_3_.

**Figure S37.** ^1^H^13^C HSQC spectrum of monomer **Me-LU** in CDCl_3_.

**
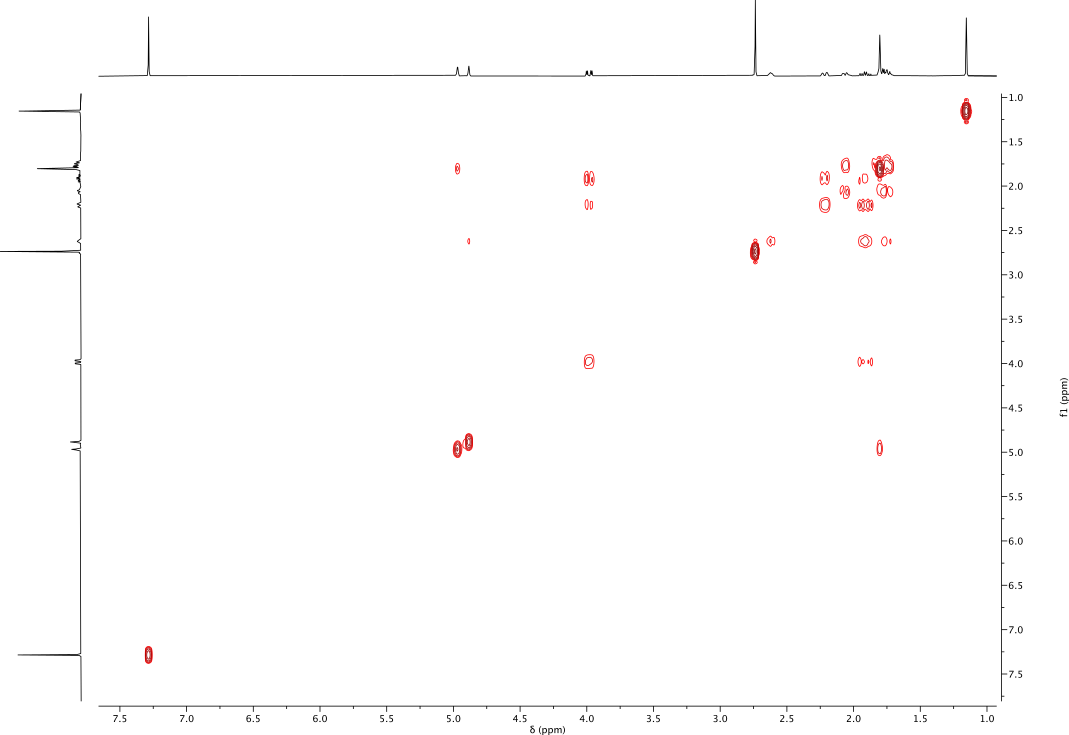
**

**Figure S38.** ^1^H^1^H COSY spectrum of monomer **Me-LU** in CDCl_3_.

3****. Polymerization OF LU****


**All polymerizations were carried out in a glove box under argon atmosphere. At room temperature LU (50.0 mg, 256 µmol, 1.00 eq.) was placed in a crimp vial equipped with a stirring bar and stock solutions of activator A1 and catalyst were added to reach the desired equivalents and a concentration of 1 M monomer in THF or toluene (256 µL). The vial was closed and placed in a preheated aluminum block.** Polymerizations were performed at a given temperature for a certain time (for detailed information see Table 1) and quenched by addition of wet CDCl_3_. **The polymers were precipitated twice form THF with *n*-pentane, separated *via* centrifugation and dried under vacuum at 80 °C for 48 hours.**

^1^H NMR (400 MHz, CDCl_3_) *δ* (ppm) = 5.23 (bs, 1H, N‑H), 4.71 (d, 2H, ^2^*J*_HH_ = 14.1 Hz, H‑11), 4.58 (s, 1H, H‑1), 2.21 (s, 1H, H‑6), 1.97 (s, 1H, H‑5), 1.88‑1.74 (m, 2H, H‑4, H‑5), 1.69 (s, 3H, H‑10), 1.67‑1.53 (m, 2H, H‑4, H‑7), 1.43‑1.25 (m, 4H, H‑7, H‑8).

^13^C{^1^H} NMR (100 MHz, CDCl_3_) *δ* (ppm) = 148.72 (C‑2), 143.67 (C‑9), 109.30 (C‑11), 72.21 (C‑1), 54.37 (C‑3), 38.01 (C‑6), 31.18 (C‑4), 25.91 (C‑7), 22.77 (C‑5), 21.22 (C‑8, C‑10).

**Table S1.** Screening of potential organometallic initiators or catalysts for the ROP of **LU**.

| Entry | Catalyst | **LU**/**A1**/Cat | Solvent^[a]^ | *t*_Pol_ | *T*_Pol_ | *X*_LU_^[b]^ | *M*_n,GPC_^[c]^ | *M*_n,NMR_^[b]^ | DP^[c]^ | *Đ*^[c]^ |
| --- | --- | --- | --- | --- | --- | --- | --- | --- | --- | --- |
|  |  |  |  | [h] | [°C] | [%] | [kg/mol] | [kg/mol] |  |  |
| 1 | NaH | 50:1:1 | - | 20 | 100 | degrdn. | - | - | - | - |
| 2 | *n*-BuLi | 50:1:1 | THF | 20 | r.t. | 5 | - | - | - | - |
| 3 | *n*-BuLi / 12-crown-4-ether (1:2) | 50:1:1 | THF | 20 | r.t. | 29 | 0.5 | - | - | 1.4 |
| 4 | KO*t*-Bu | 50:1:1 | THF | 20 | r.t. | 12 | 0.4 | - | - | 1.2 |
| 5^[d]^ | [(ONOO)*^t^*^-Bu^Y(bdsa)(THF)] | 50:1:1 | THF | 20 | 60 | 37 | 0.9 | 1.4 | 3 | 2.9 |
| 6 | [(ONOO)*^t^*^-Bu^Y(bdsa)(THF)] | 50:1:1 | Toluene | 20 | 60 | 43 | 1.1 | 1.4 | 4 | 1.7 |
| 7^[d]^ | [(ONOO)*^t^*^-Bu^Y(bdsa)(THF)] | 50:0:1 | Toluene | 20 | 60 | 45 | 0.8 | - | 4 | 1.5 |
| 8^[d]^ | [(ONOO)*^t^*^-Bu^Y(bdsa)(THF)] | 50:1:1 | Toluene | 20 | 100 | 18 | 1.1 | 1.5 | 4 | 2.1 |
| 9 | [(ONNO)*^t^*^-Bu^In(O*t*-Bu)] | 50:1:1 | THF | 20 | 60 | - | - | - | - | - |
| 10 | [(ONNO)*^t^*^-Bu^In(O*t*-Bu)] | 50:1:1 | Toluene | 20 | 100 | - | - | - | - | - |
| 11 | [(ONNO)*^t^*^-Bu^In(O*t*-Bu)] | 50:0:1 | Toluene | 20 | 100 | 71 | 3.0 | - | 15 | 1.7 |
| 12 | - | 50:0:0 | Toluene | 20 | 100 | - | - | - | - | - |
| 13 | - | 50:1:0 | Toluene | 20 | 100 | - | - | - | - | - |
| ^[a]^ Reaction conditions: [**LU**] = 1 M except for entry 1. ^[b]^ Conversion of **LU** (X_Lu_) and number average molar mass (*M*_n,NMR_) related to the benzoyl end group of **A1** determined *via* ^1^H NMR in CDCl_3_ (see Figure S37-S38). ^[c]^ Number-average molar mass (*M*_n,GPC_), degree of polymerization (DP), and polydispersity index (*Đ* = *M*_w_/*M*_n_) determined *via* gel permeation chromatography (GPC) in DMF with 2.096 g/L LiBr added at 30 °C referenced to poly(methylmethacrylate) calibration standards. ^[d]^ Bimodal distribution. | | | | | | | | | | |


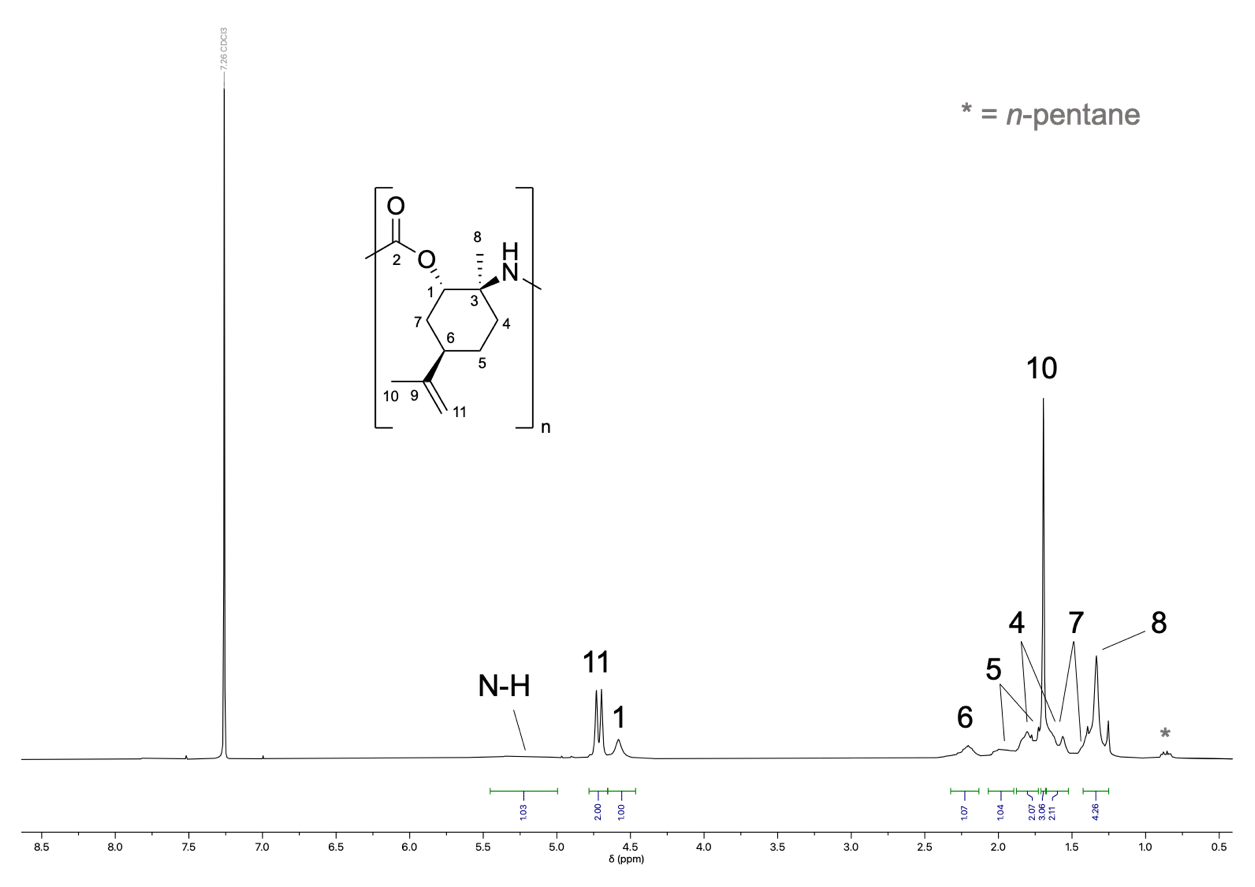


**Figure S39.** ^1^H NMR spectrum of polymer **PLU** in CDCl_3_.


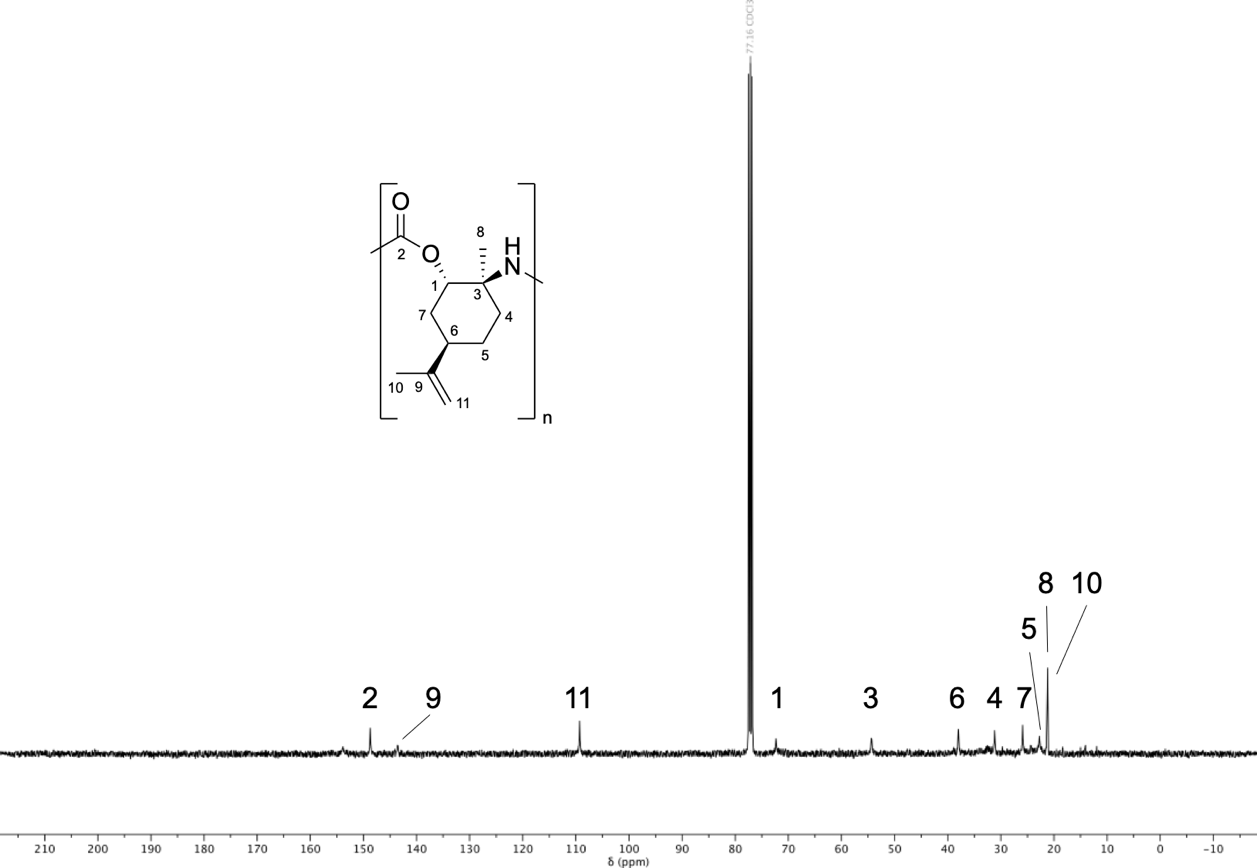


**Figure S40.** ^13^C{^1^H} NMR spectrum of polymer **PLU** (prepared without **A1**) in CDCl_3_.


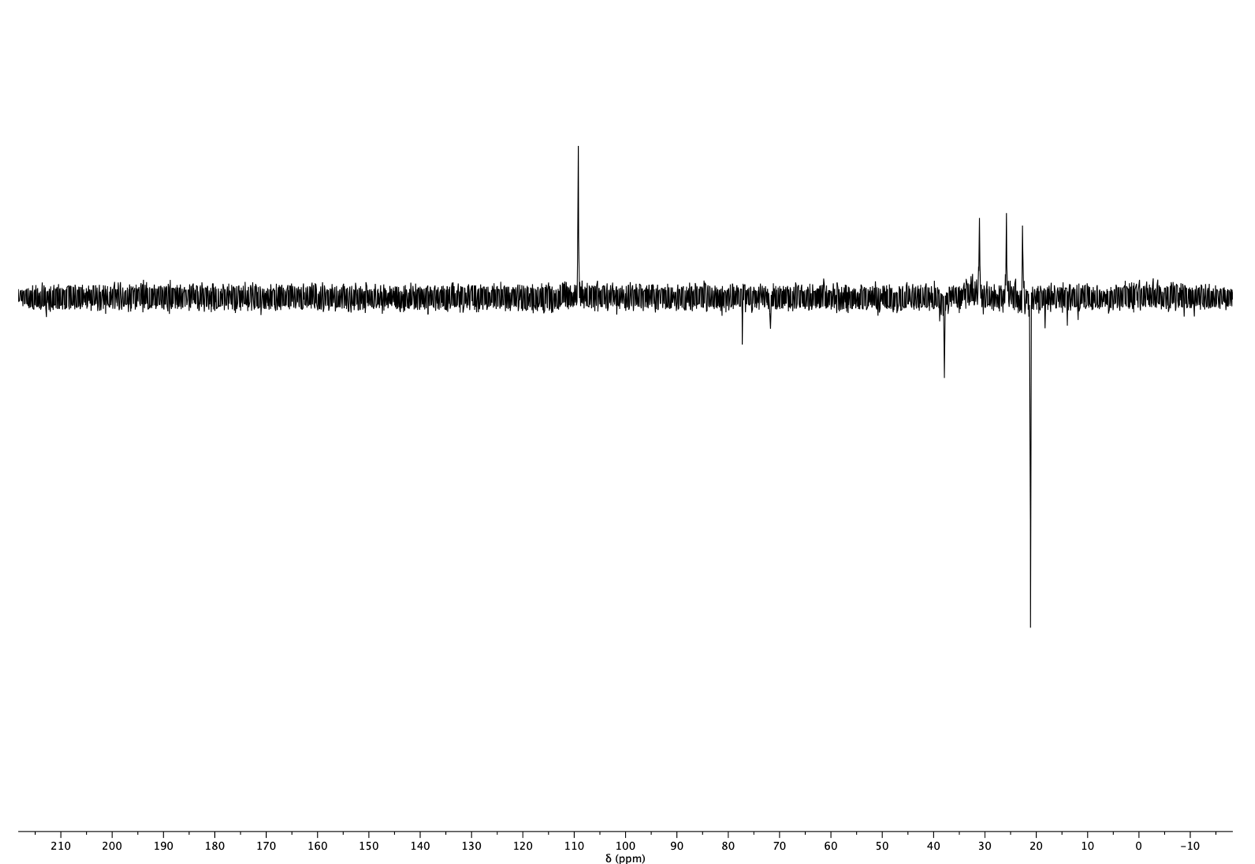


**Figure S41.** DEPT 135 spectrum of polymer **PLU** in CDCl_3_.


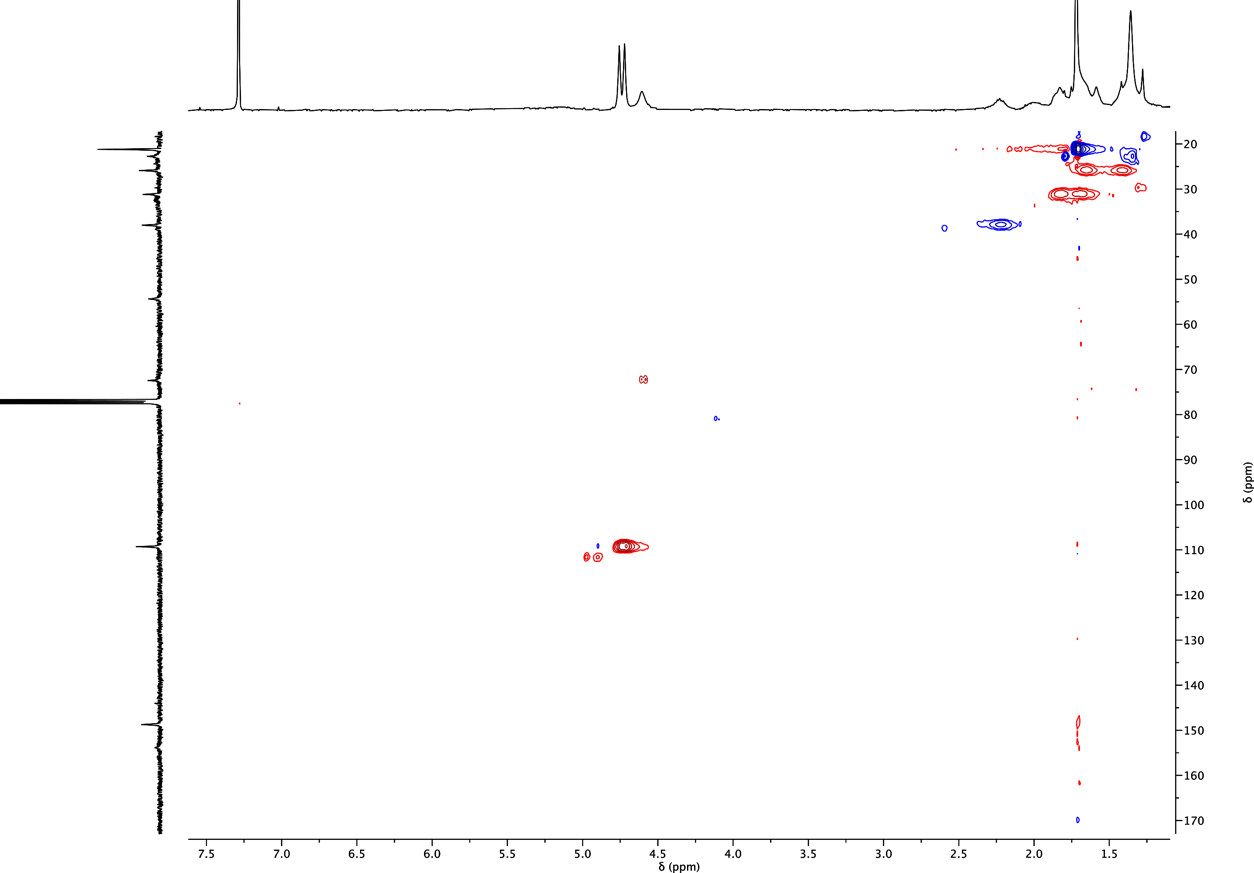


**Figure S42.** ^1^H^13^C HSQC spectrum of polymer **PLU** in CDCl_3_.


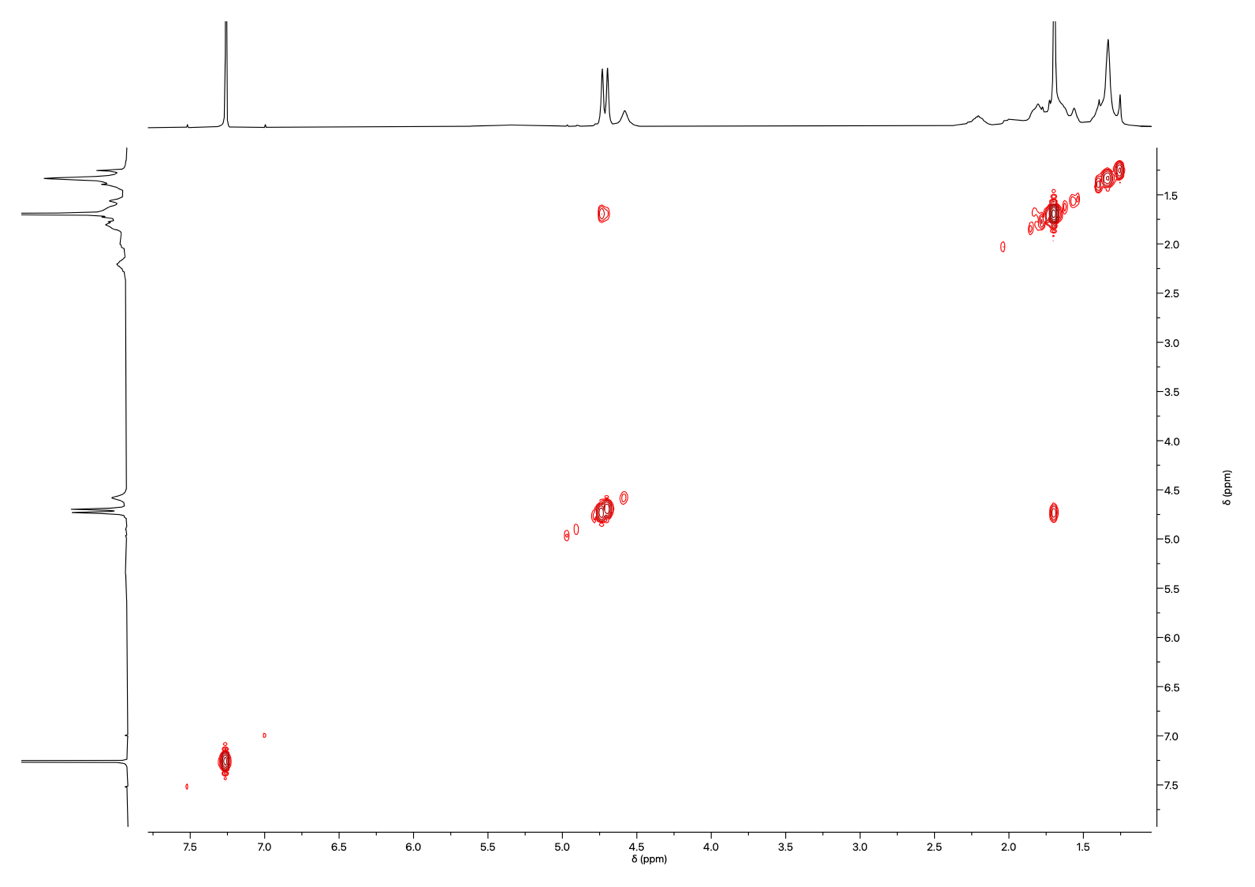


**Figure S43.** 1H1H COSY spectrum of polymer **PLU** in CDCl_3_.


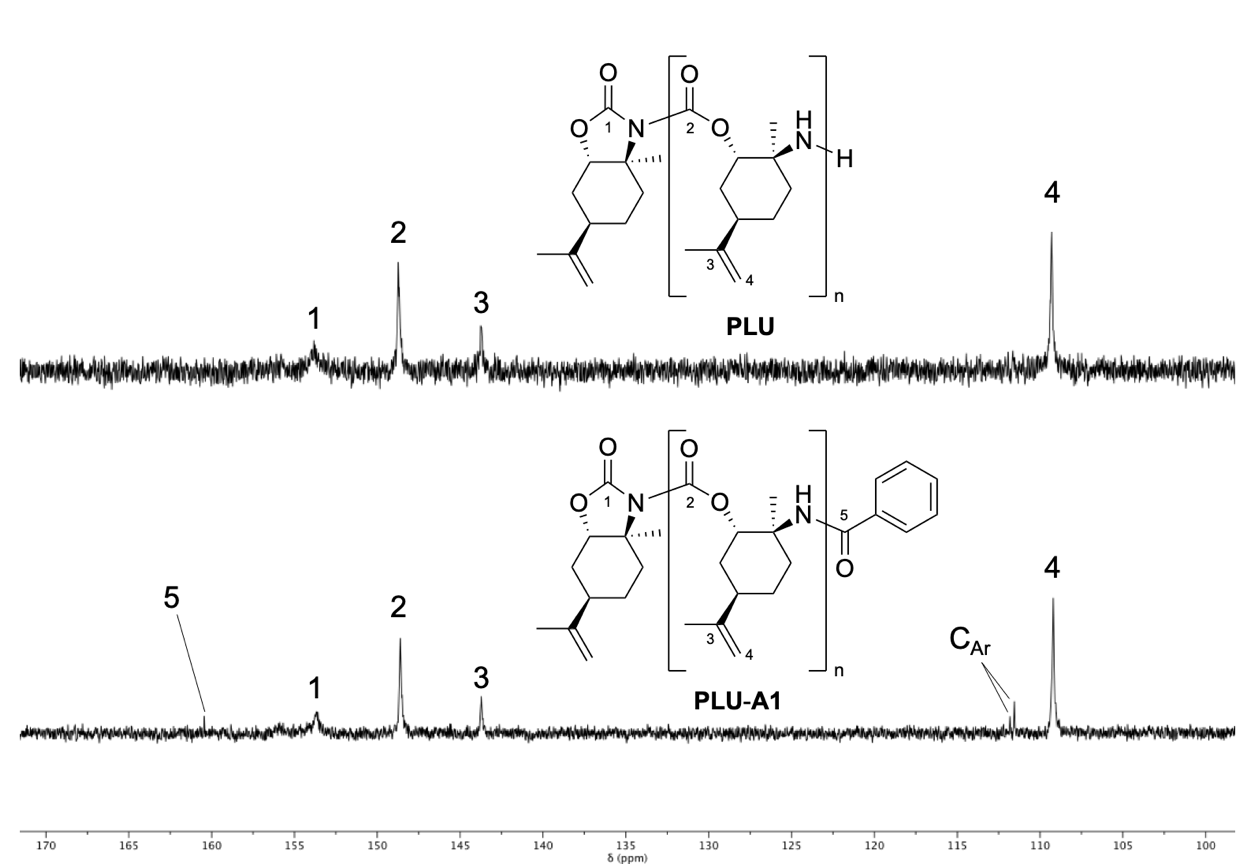


**Figure S44.** ^13^C{^1^H} NMR spectra comparison of polymer **PLU** prepared with **A1** (bottom) and without **A1** (top) in CDCl_3_.

**Figure S45.** ^1^H^15^N HMBC spectrum of polymer **PLU** prepared without **A1** in CDCl_3_.


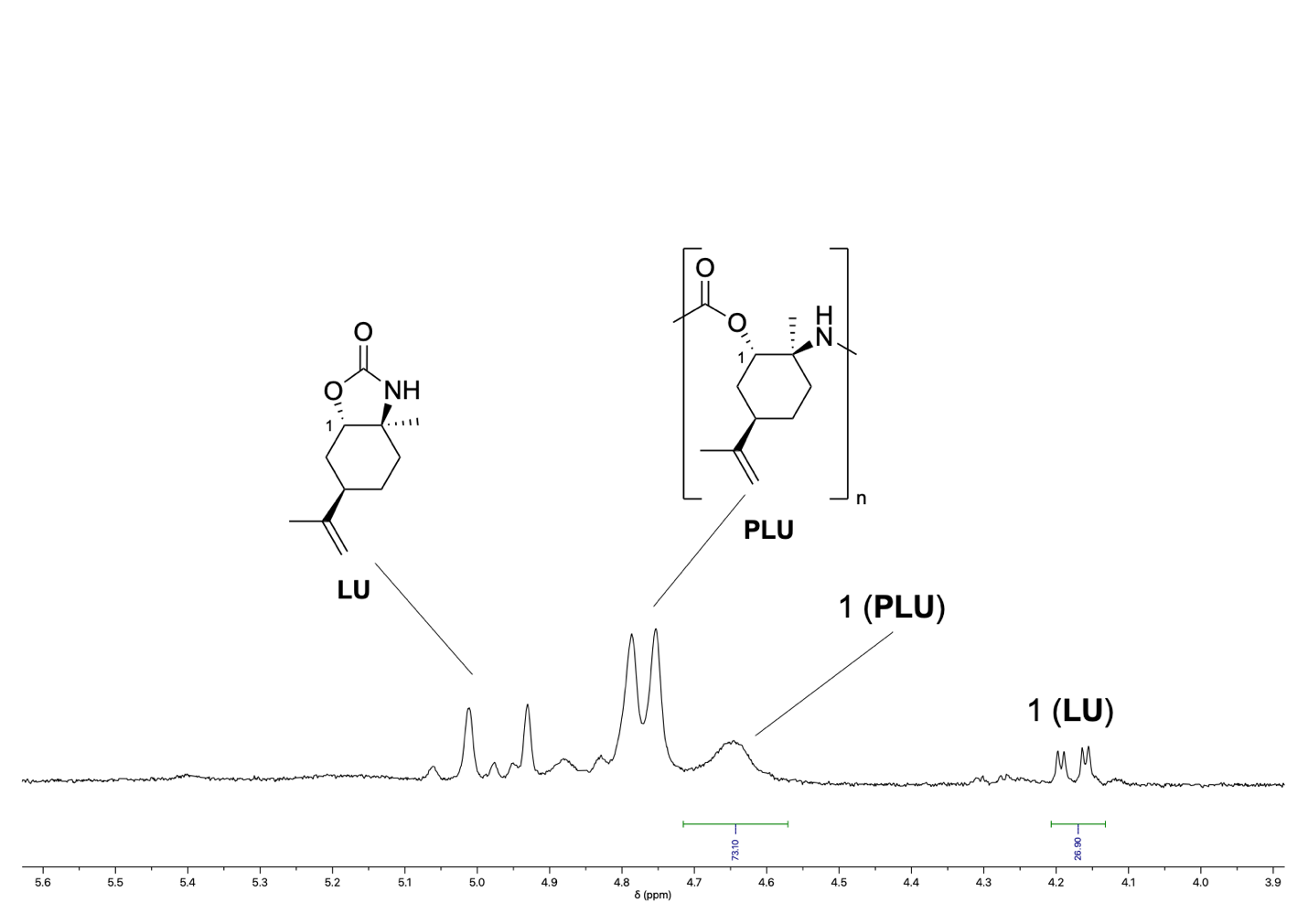
**Figure S46.** Exemplary ^1^H NMR analysis in a mixture of toluene and wet CDCl_3_ to determine the conversion of **LU** by integrating the C-1 unit of monomer **LU** and polymer **PLU** (see Table 1 entry 4).


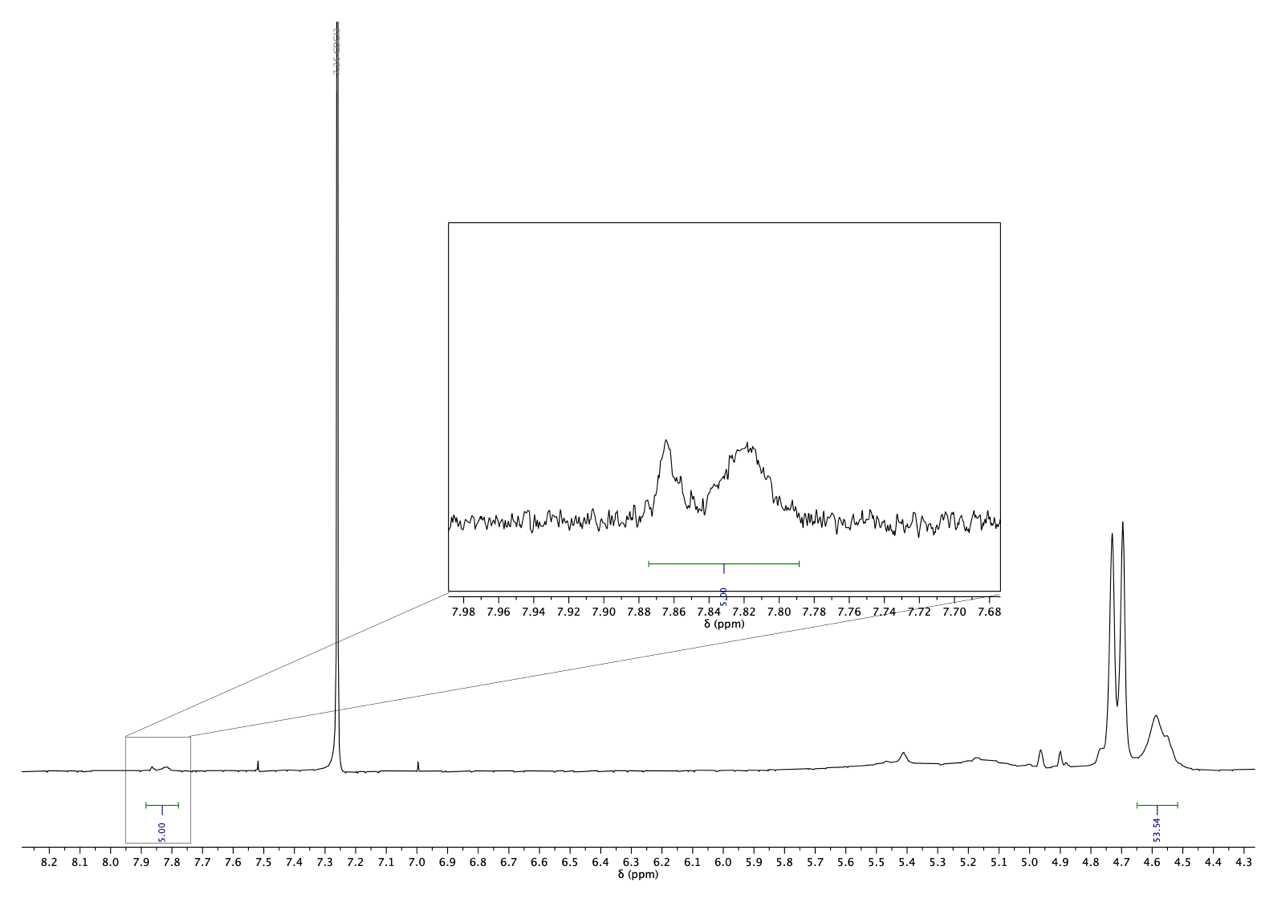


**Figure S47.** Excerpt from the ^1^H NMR spectrum of precipitated **PLU** in CDCl_3_ for calculating the degree of polymerization (DP) and the average molecular weight *M*_n,NMR_ *via* the benzoyl end group. Synthesized with a ratio of **LU**/**A1**/Sn(Oct)_2_ of 50:1:1 (Table 1 entry 5).

**Figure S48.** Exemplary comparison of **PLU** samples prepared with activator **A1** (orange curve, Table 1 entry 9) and without **A1** (blue curve, Table 1 entry 11). In the presence of **A1** narrower values for *Đ* are observed.

**Figure S49.** MALDI-MS spectra of precipitated **PLU** samples. (**A**) Sample prepared with activator **A1** (Table 1 entry 9). (**B**) Sample prepared without **A1** (Table 1 entry 11).

**
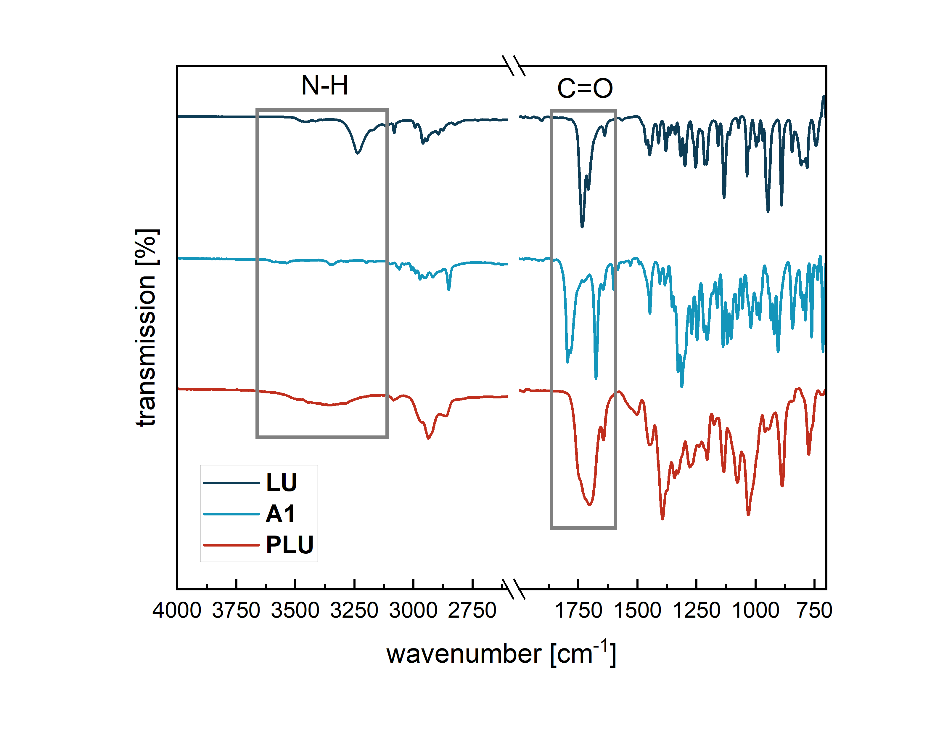
**

**Figure S50.** IR spectra of monomer **LU**, activator **A1**, and polymer **PLU**. The polymerization of **LU** broadens both the N-H and C=O bands.

**Figure S51.** (**A**) Plot of **LU** conversion against time for varying initial [**LU**]. Reaction conditions: [Sn(Oct)_2_] = 0.02 M, [**A1**] = 0.02 M, 100 °C in toluene. (**B**) Plot of ln(*v*_0_) against ln([**LU**]) to determine the reaction order of **LU**.

**Figure S52.** (**A**) Plot of [**LU**] against catalyst t[Sn(Oct)_2_]^n^, n = 0.4; the fit suggests a 0.4 order in catalyst concentration. Reaction conditions: [**LU**] = 1.00 M, [**A1**] = 0.02 M, 100 °C in toluene. (**B**) Plot of [**LU**] against activator t[**A1**]^p^, p = 0; the fit suggests a zeroth order in activator concentration. Reaction conditions: [**LU**] = 1.00 M, [Sn(Oct)_2_] = 0.02 M, 100 °C in toluene.

4****. NINHYDRIN END GROUP ANALYSIS****

**All stoichiometric reactions were carried out in a glove box under argon atmosphere. At room temperature LU (25.0 mg, 128 µmol, 1.00 eq.) was placed in a crimp vial equipped with a stirring bar and stock solutions of activator A1 and Sn(Oct)_2_ were added to reach the desired equivalents and a concentration of 1 M monomer in toluene (128 µL). The vial was closed and placed in a preheated aluminum block.** The reactions were stirred for 5 hours, quenched by addition of ninhydrin (2.00 eq.) dissolved in ethanol (20 mg/mL) and refluxed for 1 minute.

**Figure S53.** End group analysis of **PLU** by stoichiometric reactions. (**A**) The reaction of **LU** in the presence of Sn(Oct)_2_ (2:1) results in *Ruhemann’s* purple upon addition of ninhydrin, revealing the amine terminated polymer chain. (**B**) The reaction of **LU**/**A1**/Sn(Oct)_2_ (1:1:1) leads to no color change upon the addition of ninhydrin.

5****. Methylated LU****


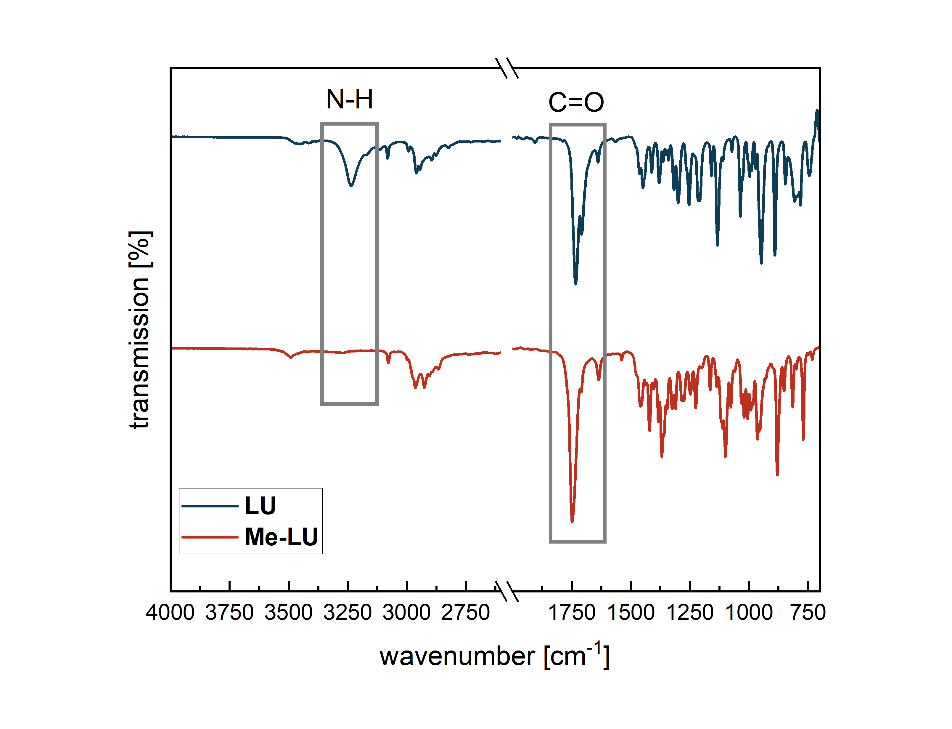


**Figure S54.** IR spectra comparison of monomer **LU** (blue spectrum) and the *N-H* protected **Me-LU** compound (red spectrum). The N-H band for **Me-LU** disappears due to methylation.


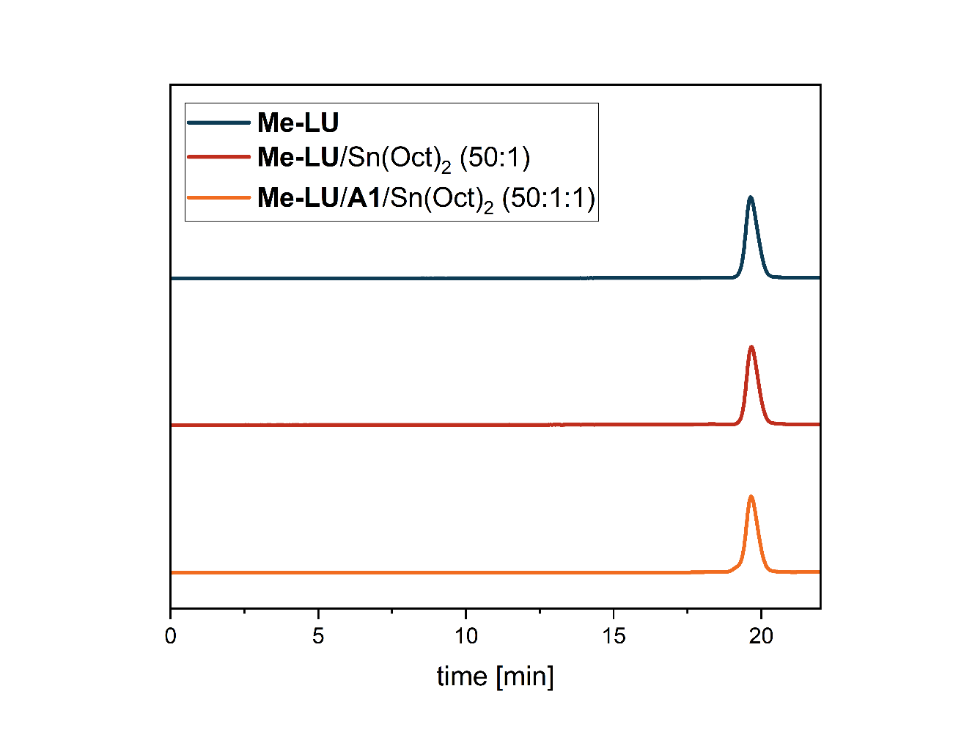


**Figure S55.** The GPC measurements of **Me-LU** (blue curve) and the two polymerizations thereof with Sn(Oct)_2_ (red and orange curve) show that no oligomers or polymers were obtained.

**Table S2.** Attempts to polymerize **Me-LU** under the same reaction conditions as applied for **LU**.

| *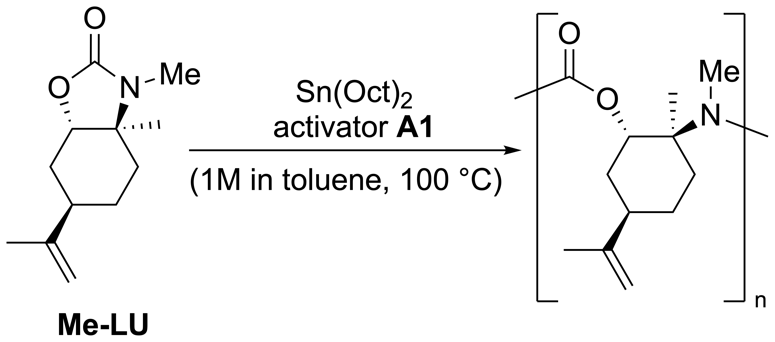* | | | | |
| --- | --- | --- | --- | --- |
| Entry | **Me-LU**/**A1**/Sn(Oct)_2_^[a]^ | *t*_Pol_ | *X***_Me-LU_**^[b]^ | *M*_n_^[c]^ |
|  |  | [h] | [%] | [kg/mol] |
| 1 | 50:0:1 | 20 | - | - |
| 2 | 50:1:1 | 20 | - | - |

^[a]^ Reaction conditions: [**Me-LU**] = 1 M in toluene at 100 °C. ^[b]^ Conversion of **Me-LU** (*X***_Me-LU_**) determined *via* ^1^H-NMR in CDCl_3_. ^[c]^ Number-average molar mass (*M*_n_), degree of polymerization (DP), and polydispersity index (*Đ* = *M*_w_/*M*_n_) determined *via* gel permeation chromatography (GPC) in DMF with 2.096 g/L LiBr added at 30 °C referenced to poly(methylmethacrylate) calibration standards.

6****. Chemical recyclability and hydrolytic degradation****

**Depolymerization in solution**: **PLU (50 mg) was suspended in mesitylene (2.56 mL, 0.1 M) and Sn(Oct)_2_ (10.4 mg, 10 mol% relative to monomer LU). The mixture was heated to 140 °C** for 2 hours and the crude product was analyzed *via* ^1^H NMR and GC-MS. GC-yield: 94%.

**Figure S56.** GC-MS traces of **LU** (blue chromatogram) and the crude product after **PLU** depolymerization in mesitylene at 140 °C using Sn(Oct)_2_ as catalyst (green chromatogram).

**Depolymerization in bulk**: **In a vacuum sublimation setup, PLU (100 mg) was dissolved in THF and suspended with ZnCl_2_ (6.98 mg, 10 mol% relative to monomer LU). After evaporation of the solvent, the mixture was heated to 200 °C at** 10^-5^ mbar for 2 hours to afford 24.8 mg of a product mixture, which was analyzed *via* ^1^H NMR and GC-MS. The yield of the **LU** fraction amounted to 15%.

**Figure S57.** Stacked NMR spectra of starting monomer **LU** (**A**) and the crude product mixture after depolymerization of **PLU** at 200 °C using ZnCl_2_ as catalyst (**B**).

**Figure S58.** GC-MS traces of **LU** (blue chromatogram) and the crude product mixture after depolymerization of **PLU** at 200 °C using ZnCl_2_ as catalyst (green chromatogram).

**Basic hydrolysis:** An aqueous 5.0 M potassium hydroxide solution (5 mL) was added to a screw cap vial loaded with **PLU** (50.0 mg). The closed reaction vial was continuously stirred at 70 °C for 7 days. Aliquots of the suspension were taken daily, dried, resuspended in chloroform, and filtered to remove potassium hydroxide. The dried samples were then analyzed using GPC. After 7 days, the remaining turbid reaction solution was neutralized with aqueous hydrochloric acid, filtered, and extracted with chloroform (3 × 2 mL). After evaporation of the solvent, the crude low molecular weight products were analyzed by GC-MS.

**Figure S59.** Plot of *M*_n_ change over 7 days due to hydrolytic degradation of **PLU** (*M*_n,0_ = 12.3 kg/mol) in 5 M aqueous potassium hydroxide solution at 70 °C.

**Figure S60.** GC-MS traces of **LU** (blue chromatogram) and the degradation products after hydrolytic degradation of **PLU** in 5.0 M potassium hydroxide solution at 70 °C for 7 days.

**7. REFERENCES**

(1) Doumeng, M.; Makhlouf, L.; Berthet, F.; Marsan, O.; Delbé, K.; Denape, J.; Chabert, F. A Comparative Study of the Crystallinity of Polyetheretherketone by Using Density, DSC, XRD, and Raman Spectroscopy Techniques. *Polym. Test.* **2021**, *93*, 106878.

(2) Hübschle, C. B.; Sheldrick, G. M.; Dittrich, B. ShelXle: A Qt Graphical User Interface for SHELXL. *J. Appl. Crystallogr.* **2011**, *44* (6), 1281–1284.

(3) Sheldrick, G. M. Crystal Structure Refinement with SHELXL. *Acta Crystallogr. Sect. C* **2015**, *71* (1), 3–8.

(4) Wilson, A. J. *International Tables for Crystallography*, Vol. C.; Kluwer Academic Publishers: Dordrecht, The Netherlands, 1992.

(5) Spek, A. L. Structure Validation in Chemical Crystallography. *Acta Crystallogr. D. Biol. Crystallogr.* **2009**, *65* (Pt 2), 148–155.

(6) Huang, Z.-Y.; Jiao, M.-R.; Gu, X.; Zhai, Z.-R.; Li, J.-Q.; Zhang, Q.-W. Asymmetric Synthesis of 1,2-Limonene Epoxides by Jacobsen Epoxidation. *Pharm. Front.* **2021**, *03* (03), e113–e118.

(7) Cimarelli, C.; Fratoni, D.; Palmieri, G. A Convenient Synthesis of New Diamine, Amino Alcohol and Aminophosphines Chiral Auxiliaries Based on Limonene Oxide. *Tetrahedron Asymmetry* **2009**, *20* (19), 2234–2239.

(8) Tamura, M.; Honda, M.; Noro, K.; Nakagawa, Y.; Tomishige, K. Heterogeneous CeO_2_-Catalyzed Selective Synthesis of Cyclic Carbamates from CO_2_ and Aminoalcohols in Acetonitrile Solvent. *J. Catal.* **2013**, *305*, 191–203.
